# Van der Waals Heterostructures for Photoelectric, Memory, and Neural Network Applications

**DOI:** 10.1002/smsc.202300213

**Published:** 2024-02-14

**Authors:** Hang Xu, Yue Xue, Zhenqi Liu, Qing Tang, Tianyi Wang, Xichan Gao, Yaping Qi, Yong P. Chen, Chunlan Ma, Yucheng Jiang

**Affiliations:** ^1^ Jiangsu Key Laboratory of Micro and Nano Heat Fluid Flow Technology and Energy Application School of Physical Science and Technology Suzhou University of Science and Technology Suzhou Jiangsu 215009 P. R. China; ^2^ Advanced Institute for Materials Research (WPI‐AIMR) Tohoku University Sendai 980‐8577 Japan; ^3^ School of Materials Science and Engineering Shanghai University Shanghai 200444 P. R. China; ^4^ Department of Engineering Science Faculty of Innovation Engineering Macau University of Science and Technology Av. Wai Long Macau SAR 999078 China; ^5^ Department of Physics and Astronomy and Elmore Family School of Electrical and Computer Engineering and Birck Nanotechnology Center and Purdue Quantum Science and Engineering Institute Purdue University West Lafayette IN 47907 USA; ^6^ Institute of Physics and Astronomy and Villum Center for Hybrid Quantum Materials and Devices Aarhus University 8000 Aarhus‐C Denmark

**Keywords:** neural networks, photoelectric devices, van der Waals heterostructures, 2D materials

## Abstract

A van der Waals (vdW) heterostructure is formed by combining multiple materials through vdW bonds. It can combine the advantages of electronic, optical, thermal, and magnetic properties of different 2D materials and has the potential to develop into the next generation of high‐performance functional devices. Herein, the current research advances of vdW heterostructures are reviewed. First, current fabrication methods and physical structures of vdW heterostructures are summarized. The 2D/nD (*n* = 0,1,2,3) mixed‐dimensional heterostructures are discussed in detail. Second, a new type of vdW heterostructure is introduced based on two‐dimensional electron gas with a nanoscale junction interface. Finally, the application prospects of vdW heterostructures in photoelectric and memory devices are further outlined by combing new applications in the neural networks. This review shows that vdW heterostructures have great advantages in high integration, energy harvesting, and logical operations, and it provides directions and suggestions for the future research and application of environmentally friendly, high‐performance, and smart functional devices.

## Graphical abstract

This article comprehensively summarizes the low‐dimensional material heterostructures from the aspects of production methods, physical properties, and applications and reviews the application of 2D/nD (*n* = 0, 1, 2, 3) heterostructures in photoelectricity, memory, and neural network. The discussion of the current problems and prospects can inspire more researchers to rationally explore and design high‐performance and multifunctional vdW heterostructures.

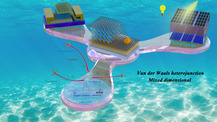

## 1 Introduction

The family of semiconductor heterostructures is an important basis of current electronic and photoelectronic devices. Their structures rely on atomic bonding between materials and usually depend on the lattice structure of the material. Usually, lattice mismatch leads to interfacial dislocations, so it is difficult for materials with large differences in lattice structures to form a good heterostructure interface. Two‐dimensional materials (2DMs) without surface dangling bonds may be a candidate to solve this issue. Graphene (Gr) is the first and most studied 2DM, and the numerous applications and achievements arising from the study of Gr have stimulated researchers’ great interest in 2DMs.^[^ ^1^ ^]^ So far, hundreds of 2DMs have been reported, which can be roughly divided into many different categories based on their chemical elements and structures. Some examples include 1) monoelemental layered 2DMs (X‐enes) (e.g., tellurene^[^ ^2^ ^]^); 2) X‐anes;^[^ ^3^, ^4^ ^]^ 3) fluoro‐X‐enes;^[^ ^5^ ^]^ 4) transition metal dichalcogenides (TMDCs);^[^ ^6^ ^]^ 5) semimetal chalcogenides (SMCs);^[^ ^5^ ^]^ 6) metal nitrides/carbides (MXenes);^[^ ^7^, ^8^ ^]^ 7) layered metal oxides;^[^ ^9^ ^]^ 8) layered double hydroxides;^[^ ^9^ ^]^ 9) and graphitic carbon nitride.^[^ ^10^ ^]^ In **Table** **1**, we discuss in detail the different categories of 2DMs in terms of composition, structure, and properties.

**Table 1:** The summary of categories of 2D materials

Categories	Chemical composition	Materials	Structure and properties
Monoelemental Layered 2D materials	Elemental 2D materials, often referred to as Xenes, are monoelemental materials with layered or sheet‐like structures	Gr	Graphene is a single‐layer 2D crystal structure composed of carbon atoms, possessing several distinctive structural and physical characteristics. Carbon atoms in graphene are arranged in a hexagonal lattice, forming a structure resembling a honeycomb. Graphene exhibits extremely high electrical conductivity, ranking among the best conductors known. Graphene possesses remarkable thermal conductivity, making it potentially significant in thermal management applications. Graphene has exceptional strength, being 200 times stronger than steel while remaining extremely lightweight. Despite its high strength, graphene is flexible and can be bent and stretched without breaking. Graphene demonstrates good transparency to visible light, holding potential for optical applications. At extremely low temperatures, graphene exhibits the quantum Hall effect, demonstrating unique electronic behavior.
		BP	BP is a layered material in which individual atomic layers are stacked together by vdW interactions, much like bulk graphite. Inside a single layer, each phosphorus atom is covalently bonded with three adjacent phosphorus atoms to form a puckered honeycomb structure. The three bonds take up all three valence electrons of phosphorus, so, unlike graphene, monolayer black phosphorus is a semiconductor with a predicted direct bandgap of 2 eV at the G point of the first Brillouin zone. For few‐layer phosphorene, interlayer interactions reduce the bandgap for each layer added, and eventually reach 0.3 eV for bulk BP. The direct gap also moves to the Z point as a consequence.
		*α*‐tellurene	Group VI tellurium has a unique chiral‐chain crystal lattice in which individual helical chains of Te atoms are stacked together by vdW type bonds and spiral around axes parallel to the^[^ ^1^ ^]^ direction at the center and corners of the hexagonal elementary cell. Each tellurium atom is covalently bonded with its two nearest neighbors on the same chain
X‐anes	X‐anes, usually composed of lattice atoms in X‐enes connected to hydrogen atoms. In 2007, graphane was theoretically demonstrated to exist and experimentally confirmed in 2009	graphane	Graphane comes from the hydrogenation of graphene, where a hydrogen atom is introduced next to each carbon atom; graphane, similar to graphene, is a 2D alkane, and its name is also based on the nomenclature of organic chemistry, meaning saturated hydrocarbons. Compared to graphene, which is metal‐like, graphene itself is an insulator, so controlling the state of hydrogenation on graphene can exhibit semiconductor characteristics, and thus promises great applications in the field of electronic devices and transistors. When hydrogenation on graphene is incomplete, it is called hydrogenated graphene. Hydrogenated graphene can exhibit certain ferromagnetism and an energy band structure that can be modulated according to the degree of hydrogenation. In addition, it is also considered a promising material for hydrogen storage because of its reversible hydrogenation and dehydrogenation.
Fluoro‐X‐enes	which can be formed by converting hydrogen atoms in X‐anes to fluorine atoms	FGr	Fluorinated Graphene is a single atomic layer of graphene on which a fluorine is introduced next to each carbon atom, constituting a *sp3* hybridization to form a σ‐bond, and at the same time the carbon and adjacent carbon atoms are also *sp3* hybridized to form a σ‐bond. Like graphene, fluorinated graphene has excellent mechanical strength, with a tensile strength of 100 Nm^−1^. Unlike graphene, this material has a wide energy gap of 3 eV, making it transparent to visible light, and is a single‐atom‐layer‐thick insulator. In addition, the material can be stabilized in air and at a high temperature of 400 °C, similar to Teflon, which can be called “2D Teflon”.
TMDCs	which are semiconductors of the type MX_2_, where M is a transition metal atom (such as Mo or W) and X is a chalcogen atom (such as S, Se or Te)	WSe_2_	WSe_2_ has crystal structures composed of weakly bonded sandwich‐layered structures Se‐W‐Se, where a W atomic layer is embedded inside two Se layers. The WSe_2_ bulk material is a semiconductor with an indirect bandgap (1.25 eV), and the direct bandgap width of monolayer WSe_2_ is around 1.65 eV, and monolayer WSe_2_ is also a rare semiconductor material for TMDCs that possesses both n‐ and *p*‐type semiconducting properties.
		MoS_2_	Each MoS_2_ layer is 6‐7 Å thick and forms a “sandwich” structure consisting of two layers of sulfur atoms sandwiched by a hexagonally filled layer of metal atoms. In essence, the Mo‐S bonds within the layers are mainly covalent, while the sandwich layer is supported by weak vdW forces, which makes the surface layer of the crystal susceptible to exfoliation. 2 H‐MoS_2_ is a semiconducting phase with a band gap that varies with the number of layers in the range of 1.2‐1.9 eV, and 2 H‐MoS_2_ with fewer layers has distinct optical properties. The other semiconductor phase, 3 R‐MoS_2_, has physical and chemical properties similar to 2 H and only minor differences in the quantitative values of each property occur.1 T‐MoS_2_.
SMCs	which are semiconductors of the type MX, where M is a semimetal atom (such as Ga or In) and X is a chalcogen atom (such as S, Se or Te)	InSe	2D InSe consists of one or multiple numbers of Se‐In‐In‐Se atomic monolayers, which are bonded via vdW forces. Each Se atom is bonded with three neighboring In atoms via *sp3* hybridization, while having one remaining *sp3* lone pair of electrons. The lone pairs are aligned perpendicularly to the InSe plane due to the Pauli exclusion principle, and they are the strongest Lewis base in InSe, being the most reactive to Lewis acid, particularly on the surface of InSe
Mxenes	Mxenes are usually obtained by selective etching of parent MAX phases, where M is an early transition metal (such as Sc, Ti, V), A is an element of the A group, mostly group 13 or 14 of the periodic table (in particular, Al, Si, and Ga), and X stands for carbon and/or nitrogen. Mxenes have a formula of M_*n*+1_X_*n*_T_*x*_, where M and X are shown above, T_*x*_ represents surface terminations (such as O, OH, F). The *n* value can vary from 1 to 4.	Ti_3_C_2_T_*x*_	The MAX phase Ti_3_AlC_2_ structure is composed of individual Ti_3_C_2_ layers separated by Al atoms. Al is removed by treatment with HF aqueous solution, and ligands such as OH, F, etc. are attached to Ti atoms to form T_*x*_ surface terminations. Both, precursor synthesis and etching conditions, affect the Mxene composition and, therefore, properties. For example, simulations show that different surface terminations of Mxene can shift the Fermi level, as well as open or alter the band gap of the material. Properties are affected by intercalated species as well.
Layered metal oxide	That is, metal oxides with a layered structure have potential uses in energy storage and catalysis.	h‐ MoO_3_	This phase is built up by zigzag chains of MoO_6_ octahedra linked to each other by corner sharing along the c axis. The phase is generally formulated as (A_2_O)_*x*_·MoO_3_·(H_2_O)_*y*_, where A is an alkali‐metal ion or ammonium ion. The exact values of *x* and *y* depend on the details of the preparation and subsequent treatment. Here a different connectivity between chains (connecting through adjacent rather than opposing oxygens) gives rise to the hexagonal symmetry. This results in the formation of hexagonal tunnels (radius of sphere ≈1.5–1.6 Å) containing the alkali metal ions.
Layered double hydroxides	[$M_{1-x}^{2+}M_{x}^{3+}$(OH)_2_]^*x+*^($A_{x/n}^{n-}$).yH_2_O is general formula, in which M^II^ is a divalent metal ion (e.g., Mg^2+^, Ni^2+^, Co^2+^, etc.), M^III^ is a trivalent metal ion (e.g., Al^3+^, Cr^3+^, Fe^3+^, etc.), A^n−^ is an anion, and charge density of LDH layers (e.g., ${\text{CO}}_{3}^{2-}$, Cl^−^, ${\text{NO}}_{3}^{-}$, ${\text{SO}}_{4}^{2-}$), × = M^III^/M^II^ + M^III^ whose value lies between 0.2 and 0.33 for pure LDH phase. It is a candidate material in water treatment catalysis	Ru_1_/D‐NiFe LDH	Atomically dispersed Ru is immobilized on D‐NiFe LDH nanosheets by coordinating with O atoms. Ru_1_/D‐NiFe LDH shows excellent water splitting performance as an ideal model, and is a promising electrocatalyst worthy of development
Graphitic carbon nitride	Graphitic carbon nitride is not only the most stable allotrope of carbon nitrides at ambient atmosphere, but it also has rich surface properties that are attractive for many applications, including catalysis, due to the presence of basic surface sites.	g‐C_3_N_4_	g‐C_3_N_4_ is a planar two‐dimensional lamellar structure approximating graphene with two basic units, a triazine ring (C_3_N_3_) and a 3‐s‐triazine ring (C_6_N_7_) as the basic structural unit extending infinitely to form a mesh structure, and the 2D nanosheets are bonded to each other by vdW forces. Density‐functional theory calculations show that the 3‐s‐triazine ring structure is more stable than the g‐C_3_N_4_ connected by the triazine ring structure.
Hexagonal boron nitride	Hexagonal boron nitride is the only boron nitride phase that exists in nature, belonging to the hexagonal crystal system, white in color, with layer structure features and lattice parameters similar to graphene. It has excellent high‐temperature resistance, thermal shock resistance, high‐temperature thermal stability, corrosion resistance, and high dielectric constant.	h‐BN	h‐BN, like graphene, is a hexagonal crystal system. The lattice constant *a* = 0.250 nm in the in‐plane direction, the thickness of the monolayer is 0.33 nm, and the hybridization between the B and N atoms by means of *sp2* has a B‐N covalent bond length of 0.1446 nm.

Phosphorus, as a 2DM, is mainly represented by black phosphorus (BP), comprising layers of phosphorus atoms; it is one of the most studied and understood 2D phosphorus materials.^[^ ^11^ ^]^ Additionally, emerging in research are derivatives and functionalized 2D phosphorus materials, such as blue phosphorus^[^ ^12^ ^]^ and phosphorus compounds.^[^ ^13^ ^]^ In terms of functionalization, scientists create various properties and applications of 2D phosphorus materials by introducing different functional groups or combining phosphorus with other materials. These derivatives include nitrogen‐doped phosphorene and phosphorus‐based heterostructures.^[^ ^14^, ^15^ ^]^ As this field continues to evolve, additional 2D phosphorus materials and derivatives may emerge.

The massive emergence of 2DMs has brought unprecedented degrees of freedom to material design.^[^ ^16^ ^]^ Compared with their corresponding 3D configurations, 2DMs have larger surface area, and electrons in the materials are confined in the plane.^[^ ^17^, ^18^ ^]^ Therefore, the electronic, optical, and magnetic properties of these 2DMs are significantly distinct from their corresponding 3D materials. Dimensional reduction alters Coulomb interactions between charge carriers, which would lead to dielectric disorder in 2DMs.^[^ ^19^ ^]^ As a result of the distinctive properties of 2DMs, they are ideal materials for many applications in photoelectronic devices. The tunable bandgaps of 2DMs make them applicable to light waves at a wide range of frequencies.^[^ ^20^, ^21^, ^22^ ^]^ Unique carrier transport behavior plays an important role in energy, memory, and sensing applications.^[^ ^23^, ^24^, ^25^ ^]^ In addition, the light transmittance (generally conductive materials are opaque) and flexibility of 2DMs also provide greater freedom in device design.

At present, many studies have reported the achievements of 2D van der Waals (vdW) heterostructures as potential functional devices.^[^ ^26^, ^27^ ^]^ A number of intriguing physical properties are found in vdW heterostructures, such as multiferroicity in ferromagnetic Cr_2_Ge_2_Te_6_ and ferroelectric In_2_Se_3_ 2D heterostructures,^[^ ^28^ ^]^ the intrinsic and extrinsic disorder in TMDCs and hexagonal boron nitride (h‐BN) heterostructures,^[^ ^29^ ^]^ unique excited‐state dynamics in TMDCs heterostructures,^[^ ^30^ ^]^ giant bipolar unidirectional photomagnetoresistance (GBU‐PhMR) in WSe_2_/2DEG heterostructures,^[^ ^31^ ^]^ etc. Based on the excellent properties of vdW heterostructures, a considerable number of applications have been advanced. In the field of infrared detection, vdW heterostructures provide an infrared detector solution with high performance.^[^ ^32^ ^]^ Infrared detectors of different structures and band arrangements, including 2D/nD (*n* = 0, 1, 2, 3) vdW heterostructures, have been extensively studied. In computing, vdW heterostructures also show great advantage.^[^ ^33^ ^]^ According to Moore's law,^[^ ^34^ ^]^ the quantity of transistors on an integrated circuit undergoes a twofold increase every 18 months. This law has affected the development of integrated circuits for more than 40 years, and practitioners predict that this law will gradually fail. However, the emergence of 2DMs may continue this scaling, and 2DMs‐based transistors could become a potential replacement for silicon‐based transistors.^[^ ^35^, ^36^ ^]^ In the transverse dimension, the reduction in transistor size must be accompanied by a corresponding reduction in the thickness and length of the channel and gate dielectric. When the gate dielectric thickness is greater than the channel thickness, the drain fringe field through the gate dielectric severely affects the performance of the transistor, and when the gate length is reduced, the short‐channel effect (SCE) arises along with it.^[^ ^37^ ^]^ Meanwhile, as the thickness of the bulk semiconductor decreases, the carrier mobility in 3D bulk semiconductors experiences a rapid decline, attributed to the heightened surface scattering. In contrast, 2DMs with flawless surface structures present a distinctive prospect, given their inherent atomic thinness and smooth layers, all the while retaining elevated carrier mobility. 2D semiconductor channels are able to be thinned down to atomic‐level thickness while exhibiting excellent electrostatic control, which can effectively reduce operating voltage/current as well as energy consumption. 2DMs are naturally immune to SCE, and the clean surfaces with no dangling bonds effectively minimize carrier mobility degradation due to scattering. The rich band structure also facilitates the flexible design of diverse new logic and memory devices, with the potential to develop low‐power and high‐performance integrated circuits.^[^ ^38^, ^39^ ^]^

In this article, we review the fabrication methods and applications of 2DMs‐based heterostructures including but not limited to vdW heterostructures. First, the main fabrication methods for both 2DMs and vdW heterostructures have been discussed, as well as the advantages and disadvantages associated with each method. Some 2DMs‐based heterostructures, such as mixed‐dimensional heterostructures and 2DMs/ quasi‐2D electron gas (2DMs/Q2DEG) heterostructures, are also considered carefully. Second, we elucidate various photoelectronic and magnetic applications facilitated by vdW heterostructures, including photoelectronic memory, solar cells, and electroluminescent devices. Moreover, underlying principles and structural attributes of vdW heterostructures are discussed, such as the ferroelectric tunnel junction (FTJs), ferroelectric field‐effect transistor (FeFET), and memristor as well as. Their applications in artificial intelligence and neural networks are also introduced in term of principles, vision, and computation examples. In **Table** **2**, we summarize the heterostructures mentioned in the text. And in **Table** **3**, **4**, **5**, we compare the advantages of vdW heterostructures for single materials.

**Table 2:** 2D heterostructure‐based device applications

Structure	Application	Type	Device performance or highlight	References
MoS_2_/In_0.53_Ga_0.47_ As	Photodetector	2D/3D	Specific detectivity: >10^10^ Jones	[21]
			Broadband detection from 638 to 1310 nm	
Gr/Ti_2_O_3_	Photodetector	2D/3D	Photoresponsivity: ≈300 A W^−1^	[22]
			Specific detectivity: ≈7 × 10^8^ Jones	
GaTe/MoS_2_	Photodetector	2D/2D	Rectification ratio: 4 × 10^5^	[47]
			EQE: 61.68%	
			Photoresponsivity: 21.83 A W^−1^	
			Specific detectivity: 8.4 × 10^13^ Jones	
BP QDs/MoS_2_	Photodetector	0D/2D	Photoresponsivity: 15.7 μA W^−1^	[116]
			Specific detectivity: 3.7 × 10^−7^ Jones	
Gr/Bi_2_Te_3_ NWs	Photodetector	1D/2D	Photoresponsivity: ≈10^6^ A W^−1^.	[25]
			Noise equivalent power: ≈10^−18^ W Hz^−1/2^	
			Specific detectivity: ≈10^11^ Jones.	
MoS_2_/WS_2_	Photodetector	2D/2D	Responsivity: 2.51 × 10^5^ A W^−1^	[78]
			Specific detectivity: 4.20 × 10^14^ Jones	
			Current on/off ratio: 1.05 × 10^5^	
CsPbI_3−*x*_Br_*x*_ QDs/MoS_2_	Photodetector	0D/2D	Photogain: >10^5^	[82]
			Photoresponsivity: 5.5 × 10^4 ^A W^−1^	
			Specific detectivity: 1.95 × 10^12^ Jones	
Bi QDs/g‐C_3_N_4_	Photodetector	0D/2D	Photocurrent: 5.02 μA cm^− 2^	[84]
			Photoresponsivity: 2843.14 μA W^− 1^	
			Specific detectivity: 2.25 × 10^11^ Jones	
			Self‐powered capability	
Gr/MoTe_2_/Gr	Photodetector	2D/2D/2D	Photoresponsivity: (≈110 mA W^−1^ at 1064 nm and	[117]
			205 mA W^−1^ at 473 nm),	
			EQE: ≈12.9% at 1064 nm and ≈53.8% at 473 nm	
			Rapid response and recovery processes (a rise time of 24 μs and a fall time of 46 μs at 1064 nm)	
PdSe_2_ (2D)/CdTe (3D)	Photodetector	2D/3D	Response speed: 70 ns	[118]
			Specific detectivity: 3.3 × 10^12^ Jones	
			Photoresponsivity: 324.7 mA W^−1^	
			Polarization sensitivity of 4.4 to polarized infrared light signals	
WS_2_/ h‐BN/ PdSe_2_	Photodetector	2D/2D/2D	Dark current 15 pA; Photocurrent 20 μA	[20]
			Light on‐off ratio: 10^6^	
			Specific detectivity: 2.7 × 10^12^ Jones	
BP/ MoS_2_/Gr	Photodetector	2D/2D/2D	Specific detectivity: 2.3 × 10^10^ Jones	[20]
			Dichroic ratio: 4.9	
			Response time: 23 μs	
InSb/Si	Photodetector	2D/3D	Specific detectivity: 1.9 × 10^12^ Jones	[111]
			Photoresponsivity: 132 mA W^−1^	
			On/off ratio: 1 × 10^5^	
			Rise time: 2 μs	
			3 dB cut‐off frequency of 172 kHz	
			Response wavelength covering 635 nm, 1.55 and 2.7 μm	
			Graphene as transparent electrode	
PtS_2_/h‐BN/Gr	Photoelectric memory	2D/2D/2D	Storage time: 10^3^ s	[121]
			Memory states: 74 states (>6 bits)	
MoS_2_/SWCNTs	Photoelectric memory	1D/2D	Program/erase speed: ≈ 32/0.4 ms,	[98]
			High program/erase ratio ≈ 10^6^	
			Storage time ≈ 10^3^ s	
			Photoresponsivity ≈ 10^16^ Jones	
MoS_2_/BP/MoS_2_	Photoelectric memory	2D/2D/2D	Photoresponsivity: 1.3 × 10^6^ A W^−1^	[104]
			Specific detectivity: 1.2 × 10^16^ Jones.	
			Storage time >6 × 10^4^ s	
			Light‐to‐dark switching ratio: 1.5 × 10^7^	
			Multi‐bit storage capacity (11 storage states).	
MoS_2_/h‐BN/Gr	Photoelectric memory	2D/2D	Off‐current ≈10^−14^ A	[122]
			Optical switching on/off current ratio ≈10^6^ Memory states: 18 states (>4 bits)	
			Extended endurance ≈10^4^ program/erase cycles	
			Retention time >3.6 × 10^4^ s	
			Programming voltage: −10 V	
Gr/SiO_2_/Si_3_N_4_/SiO_2_	Photoelectric memory	2D/3D	Memory states: 100 states	[126]
			Retention time: several minutes	
BP/MoSe_2_	Solar cell	2D/2D	Calculated PCE: 23.04%	[133]
BP/MoS_2_	Solar cell	2D/2D	Calculated PCE: 14.41%	[133]
Cs_0.05_MA_0.05_FA_0.9_PbI_2.7_Br_0.3_/WS_2_	Solar cell	3D/2D	*V*_OC_: 1.15 V	[134]
			*J*_SC_: 22.75 mA cm^−2^	
			FF: 80.6%	
			PCE: 21.1%	
CH_3_NH_3_PbI_3_/HfS_2_	Solar cell	3D/2D	PCE: 28.45%	[135]
			First‐principles calculations	
MoS_2_/WSe_2_	Solar cell	2D/2D	Present a device model that is able to fully reproduce the current–voltage characteristics of type‐ii vdW heterojunctions under optical illumination	[137]
GaS/SnS_2_	Solar cell	2D/2D	*V*_OC_: 1.03 V	[138]
			PCE: 16.47%	
			Heteroatom doping strategy to boost PCE	
MoS_2_/ ZnS/CIGSe	Solar cell	2D/2D/3D	PCE: 0.76%	[139]
			*V*_OC_: 0.43 V	
			*J*_SC_: 7.54 mA cm^−2^	
			FF: 23.13%	
			Using interface passivation enhance PV behavior	
BP/MoS_2_	EL	2D/2D	Mid‐infrared light‐emitting	[144]
Gr/h‐BN/Gr	EL	2D/2D//2D	Deep‐ultraviolet light‐emitting	[97]
Gr/MoS_2_	EL	2D/2D	Femtosecond solid‐state laser	[145]
Gr/h‐BN/WS_2_/h‐BN/Gr	EL	2D/2D/2D/2D/2D	Red light‐emitting	[146]
			Continuous operation time >2 h	
MoS_2_/InSe	EL	2D/2D	Exhibited strong luminescence at a wavelength of 1020 nm with a turn‐on voltage of 0.8 V	[129]
Gr/BiP	FTJ	2D/2D	TER effect: 623%	[159]
Gr/*α*‐In_2_Se_3_	FTJ	2D/2D	TER effect: 1 × 10^8^ %	[155]
In:SnSe/SnSe/Sb:SnSe	FTJ	2D/2D/2D	TER effect: 1460%	[164]
MoS_2_/h‐BN/Gr/CuInP_2_S_6_	FeFETs	2D/2D/2D/3D	Program/erase ratio >10^7^	[167]
			Endurance >10^4^ cycles	
			Storage time: 10 years	
			Program/erase speed < 5 μs	
MoS_2_/CuInP_2_S_6_	FeFETs	2D/3D	Subthreshold swing: 28 mV dec^−1^	[169]
MoS_2_/Hf_1−*x*_Zr_*x*_O_2_	FeFETs	2D/3D	On/off ratio >10^7^	[173]
			Memory window: 0.1 V	
			Program/erase voltage: 3 V	
			Endurance: 10^3^ cycles	
			Retention: 10^4^ s	
Al_2_O_3_/*α*‐In_2_Se_3_	FeS‐FETs	3D/2D	On/off ratio >10^8^	[165]
			Maximum on current: 862 μA μm^−1^	
WS_2_/MoS_2_	Memristors	2D/2D	Switching ratio: 10^4^	[188]
			Endurance: 120 cycles	
Au/h‐BN/Au	Memristors	2D/3D	On/off ratio: 10^7^	[184]
			Set/reset: 2.7/1.3 V	
			R_HRS_: 10^9 ^Ω	
MoS_2_/Gr/h‐BN	Memristor	2D/2D/2D	On/off ratio ≈ 10^4^	[234]
			Standby power ≈ 16 pW	
h‐BN/Gr/h‐BN	Memristors	2D/2D/2D	Self‐selectivity: 10^10^	[192]
			On/off ratio >10^3^	
CsPbBr_3_ QDs/MoS_2_	ANN	0D/2D	Achieving photoelectrically modulated Boolean logic operation, dendritic integrations in both spatial and temporal modes, and Hebbian learning rules	[198]
WSe_2_/BN	Photoelectronic memory ANN	2D/2D	Switching ratio: 1.1 × 10^6^	[201]
			Memory states: 128 (7 bit)	
			Retention time >4.5 × 10^4^ s	
			Captured a specific image	
			Recording the three primary colors (red, green, and blue)	
MoS_2_/h‐BN/Gr	ANN	2D/2D/2D	The device demonstrates various features of a biological synapse, including pulsed potentiation and relaxation of channel conductance, as well as spike time dependent plasticity	[202]
WSe_2_/h‐BN/Al_2_O_3_	ANN	2D/2D/3D	Realize the transformation of light and electrical signals	[222]
h‐BN/WSe_2_	ANN	2D/2D	Realizing the functions of both the optical sensor and synapse	[225]
graphdiyne /Gr	ANN	2D/2D	Realize some typical synaptic behaviors such as associative learning by association, inhibitory postsynaptic current and excitatory postsynaptic current,	[226]
Gr/PbS QDs	ANN	2D/0D	High‐resolution, broadband image sensor	[228]
			Sensitive to ultraviolet, visible and infrared light (300 ‐ 2,000 nm)	
Pyrenyl graphdiyne/Gr/PbS QDs	ANN	2D/2D/0D	Fully light‐modulated synapses	[229]
			Logic functions and associative Learning capabilities	
BP/ReS_2_	ANN	2D/2D	Multi‐valued logic application	[232]
			Ternary inverter	
BP/ReS_2_	ANN	2D/2D	Mimic trilingual synaptic response	[233]
			Handwritten digits recognition	
			Recognition accuracy: 90%	
h‐BN/TpPa‐1‐COF	Photocatalysis	2D/2D	The H_2_ evolution rate can reach up to 3.15 mmol g^−1^ h^−1^	[18]
			Metal‐free	
Vanadate QDs/g‐C_3_N_4_	Photocatalysis	0D/2D	Visible‐light‐driven photocatalysis	[83]
MoS_2_/CdS NWs	Photocatalysis	1D/2D	Hydrogen evolution rate: 1914 μmol h^−1^ (20 mg catalyst)	[87]
			Apparent quantum yield: 46.9%	
WSe_2_/Q2DEG	–	2D/2DEG	Coexistence of photoelectric conversion and storage in vdW heterostructures	[23]
			GBU‐PhMR	[31]
BP/WS_2_	–	2D/2D	EQE: 103%	[15]
			Photoresponsivity: 500 mA W^−1^	
MoS_2_/PbS	–	2D/2D	PbS nanosheets were in situ grown on ultrathin MoS_2_	[77]
			Layers via solvothermal process to form 2D/2D heterostructures	
Bi_2_S_3_ NWs/MoS_2_	–	1D/2D	One‐step growth of the 1D/2D heterostructure from a Bi_2_S_3_ NW and a MoS_2_ monolayer by the CVD method	[85]
Sb_2_Se_3_ NWs/WS_2_	–	1D/2D	Demonstrate for the first time the direct synthesis of the 1D/2D mixed dimensional heterostructures by sequential vapor‐phase growth of Sb_2_Se_3_ NWs on WS_2_ monolayers	[86]
CsGeBr_3_/ blue phosphorus	–	3D/2D	Carrier mobility: 7.364 × 10^3 ^cm^2^ v^−1^ s^−1^	[103]
			First‐principles study	
CsSnBr_3_/ blue phosphorus	–	3D/2D	Carrier mobility: 7.815 × 10^3^ cm^2^ v^−1^ s^−1^	[103]
			First‐principles study	
Gr/WS_2_	–	2D/2D	Optoelectronic responsivity: 10^10^ V W^−1^	[120]

**Table 3:** Single‐Material systems versus vdW heterostructures for photodetection applications

Structure	Wavelength [nm]	*R* [A W^−1^]	D* [Jones]	References
MoTe_2_	700	2.6 × 10^−1^	2 × 10^13^	[252]
MoTe_2_	405	1.5‐5.3 × 10^2^	9.2‐42 × 10^8^	[253]
MoTe_2_/CsPdBr_3_	405	3.34 × 10^2^	6.2 × 10^10^	[254]
Gr/ MoTe_2_/Gr	473	87	10^12^	[255]

**Table 4:** Single‐Material systems versus vdW heterostructures for memory applications

Structure	P/E ratio	P/E speed	Endurance	Retention	References
HZO	10^6^	100 μs	10^7^	10 years	[256]
Si:HfO_2_	>100	1‐10 μs	10^5^	72 h	[257]
PZT/ZnO	10^5^	150 ms	/	10 years	[258]
P(VDF‐TrFE)/MoSe_2_	>10^5^	50 μs/2 ms	10^4^	>2 × 10^3^ s	[259]
CIPS/MoS_2_	>10^7^	5 μs	>10^4^	10 years	[167]

**Table 5:** Bandgap of 2D materials and their vdWs heterostructures

Material	*E*_g_	Material	*E*_g_
MoSe_2_	1.44	GaS	2.59
MoS_2_	1.61	GaSe	2.03
BP	0.76	SnS_2_	1.57
BP/MoSe_2_	0.85	GaS/SnS_2_	0.61
BP/MoS_2_	0.05	GaSe/SnS_2_	0.60

While vdW heterostructures offer numerous advantages, challenges persist in their preparation, structure, and application. Achieving precise control and scalability in the fabrication of vdW heterostructures remains a challenge. Ensuring reproducibility of these structures on a large scale is crucial for practical applications. During the preparation of vdW heterostructures, environmental factors or impurities in the materials may impact the quality of the structures. Controlling and reducing defects is essential for reliable device performance. The transfer of single layers of 2DMs for assembling heterostructures may result in wrinkles, tears, or misalignment, affecting the overall structure's quality. Accurate control of interfaces between different layers is crucial for tuning electronic properties.

Structure challenges include managing interlayer interactions, avoiding interface defects, and ensuring optimal charge transfer. Strain arising from lattice mismatches between different materials can affect electronic properties. Controlling and minimizing strain‐related effects are critical for device stability and performance. Achieving uniform thickness in large‐area vdW heterostructures is challenging. Thickness variations may lead to differences in electronic properties and device performance. Integrating vdW heterostructures into existing semiconductor technologies poses challenges. Ensuring compatibility with traditional processing methods and established device structures is crucial for practical applications. Variability in material properties and heterostructure quality may result in inconsistent device performance and reduced reliability. Achieving consistency and reliability is paramount for large‐scale applications.

This article is intended to help the reader understand the applications of vdW heterostructures, providing a foundation for exploring various potential uses.

## 2 Methods of Fabrication

**Figure 1:**
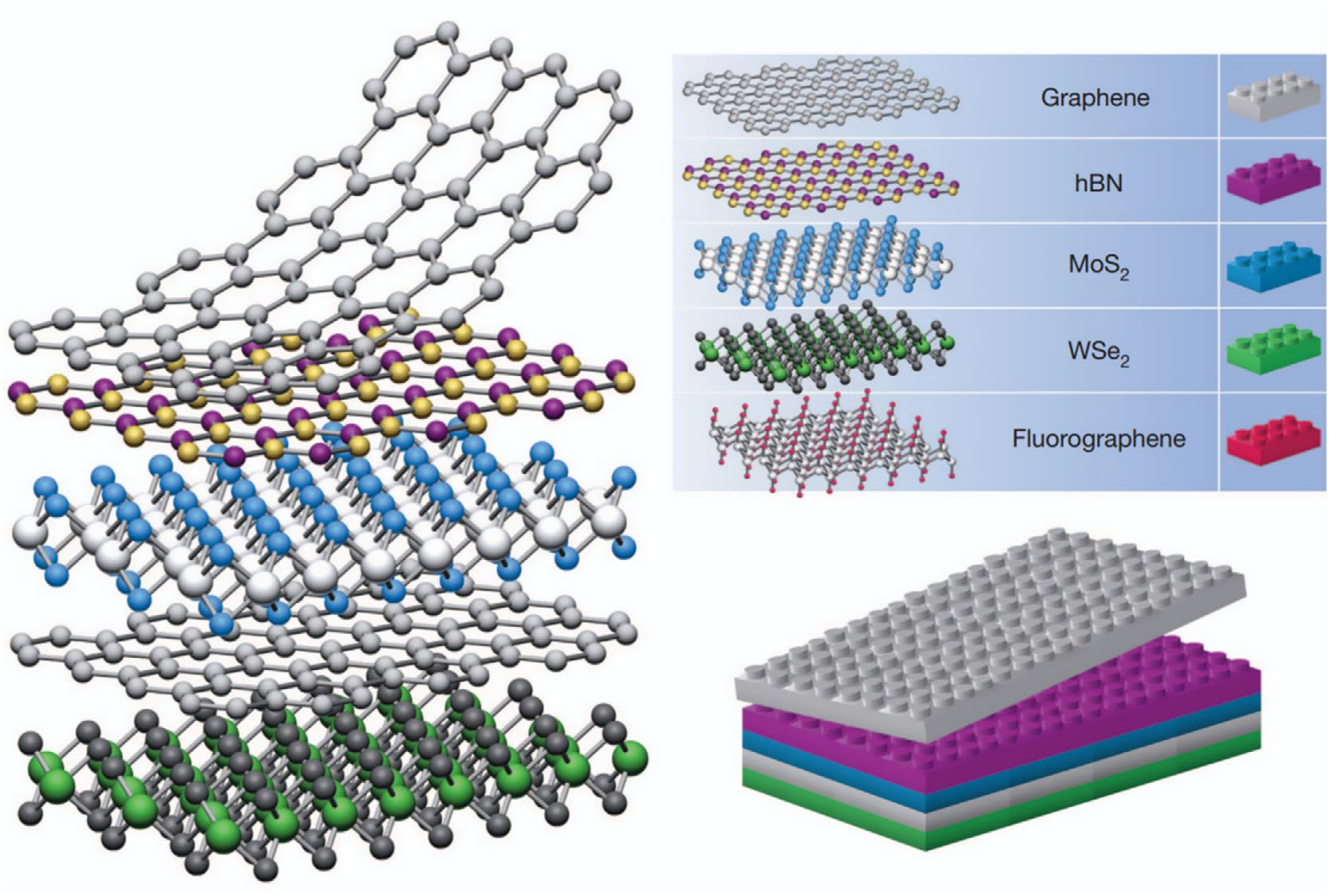
Constructing vdW heterostructures. Viewing 2D crystals as similar to Lego blocks (right panel) enables the creation of a vast of layered structures. This atomic‐scale Lego concept bears resemblances to molecular beam epitaxy (MBE) but utilizes unique “construction” guidelines and distinct materials set. Reproduced with permission.^[^ ^46^ ^]^ Copyright 2013, Nature Publishing Group, a division of Macmillan Publishers Limited. All Rights Reserved.

Since the initial preparation of Gr in 2004,^[^ ^1^ ^]^ 2DMs has grown rapidly,^[^ ^40^ ^]^ and more 2DMs subsequently flourished, such as 2D metals (NbSe_2_),^[^ ^41^ ^]^ 2D semiconductors (WSe_2_ [,^[^ ^42^ ^]^ MoS_2_^[^ ^43^ ^]^), 2D insulators (h‐BN),^[^ ^44^ ^]^ and 2D superconductors (NbS_2_).^[^ ^45^ ^]^ A. K. Geim et al. proposed that^[^ ^46^ ^]^ several 2DMs layers are combined with vdW bonds (**Figure** **1**) like Lego blocks in nature; different 2DMs are piled one on top of the other by weak vdW forces to form “vdW heterostructures.” Inspired by this assumption, the vdW heterostructure has received attention. For example, He Jun et al. successfully built an ultrathin gallium telluride/molybdenum disulfide (GaTe/MoS_2_) vdW p–n heterostructure;^[^ ^47^ ^]^ Manish Chhowalla et al. successfully built a variety of 2D semiconductors and metals between electrodes, such as monolayer MoS_2_ ultrapure vdW heterointerfaces,^[^ ^48^ ^]^ etc.

**Figure 2:**
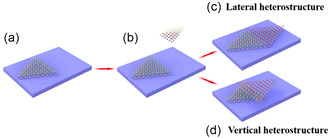
Schematic depiction of the creation of lateral and vertical heterostructures. a) Initial TMDC layer placed on the substrate. b) A second TMDC layer is assembled with the first TMDC layer. c) Two kinds of TMDCs are arranged to form a lateral heterostructure. d) Two kinds of TMDCs are arranged to form a vertical heterostructure. Reproduced under terms of the CC‐BY 4.0 license.^[^ ^49^ ^]^ Copyright 2018, The Authors. Published by Multidisciplinary Digital Publishing Institute.

VdW heterostructures can generally be obtained in two ways.^[^ ^49^ ^]^ One is to splice various layered materials in a plane to generate a lateral heterostructure (**Figure** **2**c), and the other is the vertical stacking of different layered materials layer by layer (Figure 2d) to form a vertical heterogeneous structure. It is typically synthesized by first placing a layer of 2DMs on a substrate (Figure 2a) and by the growth or transfer of another layer of material to the first layer (Figure 2b), if both layers are joined in the same level (Figure 2c), a transverse heterostructure is formed; if one layer is superimposed on the other, a vertical heterostructure will be formed (Figure 2d).

### 2.1 Fabrication of 2D Materials

**Figure 3:**
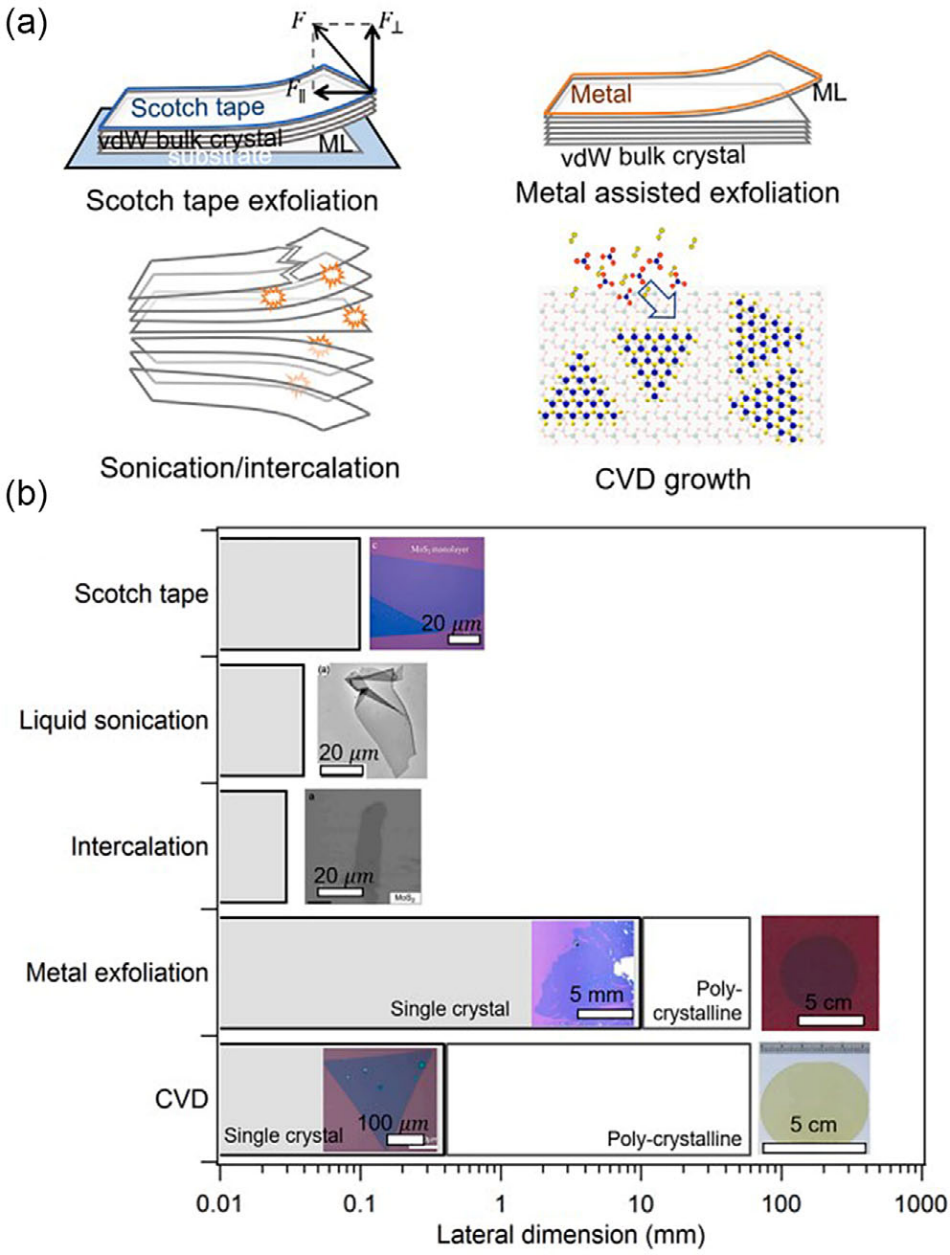
a) Illustrations of various strategies for preparing 2D monolayers, encompassing top‐down methods like mechanical exfoliation method, sonication in liquids, compound intercalation treatment, as well as the bottom‐up approach of CVD growth. b) Maximum lateral sizes achieved for monolayers of TMDCs through various methods, accompanied by corresponding visuals of the reported extensive monolayers. The example images include monolayer MoS_2_ from mechanical exfoliation,^[^ ^244^ ^]^ sonication in a solution containing organic salts in an aqueous medium,^[^ ^245^ ^]^ sodium naphthalenide‐assisted intercalation,^[^ ^246^ ^]^ 2D monolayer MoS_2_^[^ ^247^ ^]^ and 2D monolayer WS_2_ film^[^ ^248^ ^]^ from metal exfoliation, and MoS_2_ monolayers grown via CVD, including both single‐crystal^[^ ^249^ ^]^ and polycrystalline^[^ ^250^ ^]^ structures. Images adapted and reprinted with permission.^[^ ^63^, ^64^, ^65^, ^66^, ^244^, ^245^, ^246^ ^]^ Copyright, 2016 and 2017, American Chemical Society. Copyright 2018 and 2020, American Association for the Advancement of Science; Copyright 2014, 2016, and 2017, Springer Nature. (b) Reproduced with permission,^[^ ^51^ ^]^ Copyright 2021, Elsevier Ltd.

There are numerous 2DMs’ preparation techniques, including mechanical exfoliation, MBE, chemical vapor deposition (CVD), etc. Notably, synthesis methods determine the physicochemical properties of products, such as size, structure, shape as interfacial interactions, interfacial corrosion, electronic energy‐level structure, etc.^[^ ^50^ ^]^ The common routes to produce 2D single‐molecule films can be separated into two main groups:^[^ ^51^ ^]^ 1) top‐down methods to exfoliate monolayers from vdW bulk crystals, such as tape exfoliation,^[^ ^52^ ^]^ metal‐assisted exfoliation,^[^ ^53^ ^]^ liquid‐phase exfoliation,^[^ ^54^ ^]^ etc, and 2) bottom‐up methods for forming 2D crystal networks using molecular precursors, such as CVD.^[^ ^55^ ^]^ Several representative monolayer manufacturing techniques are summarized in **Figure** **3**.

Mechanical exfoliation is currently one of the most used approaches for creating 2DMs. Different forms of 2DMs can be developed on various substrates and attain a monolayer thickness by overcoming the weak vdW interactions in bulk layered crystals. Then, layer‐by‐layer stacking can be used to create vertical heterostructures.^[^ ^56^ ^]^ Tape exfoliation and metal‐assisted exfoliation are the two most prevalent mechanical exfoliation procedures. For producing high‐performance single‐crystal films with sufficiently large size and high yield, the optimal method is to exfoliate the monolayer layer by layer without tearing. For preparing large‐area single‐layer and few‐layer 2DMs, Yuan et al. proposed an improved exfoliation approach based on a home‐designed exfoliation machine.^[^ ^57^ ^]^ By mechanical exfoliation, Dastgeer et al. created stable BP/WS_2_ (BP/WS_2_) vdW heterostructure p–n diodes on Si/SiO_2._^[^ ^15^ ^]^ Huang et al. devised a generic one‐step gold‐assisted exfoliation approach^[^ ^58^ ^]^ and validated the efficiency of this technique by isolating 40 monolayer single crystals, including 2D single crystals, metal dichalcogenides, magnets, and superconductors. K. Novoselov et al. utilized a more concise method,^[^ ^59^ ^]^ which resulted in cleaner interfaces and increased electron mobility across wide portions of the material. However, tape exfoliation typically causes air bubbles amid in the bilayer stack, which reduces the effective interfacial area and causes nonuniform morphology. Polymer residues in thermal release tapes are a key source of contamination in metal‐assisted exfoliation, and they must be cleaned to reduce this influence.

A technique known as CVD involves synthesizing coatings or nanomaterials by reacting chemical gases or vapors on the surface of a substrate. The chemical reaction between different kinds of gaseous raw materials in a reaction chamber results in the formation and deposition of new material onto the wafer surface. It is the most frequently used method for depositing various materials in the semiconductor industry, including a variety of insulating materials, metals, and metal alloys. This technology allows for the production of high‐quality nonlayered 2D transition metal nitrides and transition metal carbides crystals with a variety of structures.^[^ ^60^, ^61^ ^]^ Tang et al. used vertical CVD and gaseous precursors to build monolayers of 2D TMDCs with exceptional centimeter‐scale uniformity,^[^ ^62^ ^]^ overcoming the limitations of standard solid precursors and delivering consistent mass fluxes to the growth chamber. Hong et al. used the CVD technique to make 2D MoN and MoSi_2_N_4_ with a Cu/Mo bilayer as the matrix and NH_3_ as the nitrogen supply.^[^ ^63^ ^]^ Yang et al. employed a simple CVD approach to effectively build vertical 2D MoS_2_ nanosheets/*p*‐GaN nanorod heterostructure‐based photodetector devices,^[^ ^64^ ^]^ which can be used in the visible light range and has high performance. The primary drawbacks of the CVD process are the high reaction temperature, low deposition rate, difficulty in localized deposition, and thin plating; the plated metal cannot be reground and processed, which also limits the application on steel materials and is mostly used for hard alloys.

MBE is also used to develop different heterostructures, where ultrahigh‐purity elements are heated in an electron beam evaporator until they start slowly sublime. Following their interaction, the gaseous components condense on the substrate. The deposition rate is the most important component in MBE, as the slower deposition rate promotes the epitaxial growth of 2DMs. Reflection high‐energy electron diffraction is a critical component of MBE equipment for monitoring the development of material layers in situ. Sergio Fernández‐Garrido et al. used plasma‐assisted MBE to establish a complete epitaxy and scalable growth process for fabricating single‐crystal GaN nanowires (NWs) on Gr.^[^ ^65^ ^]^ Yuan et al. also succeeded in exfoliating 10–20 nm 2D (Ga, Mn) of hundreds of micrometers from MBE‐grown substrates using the vdW heterostructure assembly technique.^[^ ^66^ ^]^ They developed a deterministic dry transfer method and fabricated h‐BN/(Ga, Mn) as top‐gate Hall devices and *p*‐(Ga, Mn)As/n‐MoS_2_ heterostructure diodes. The results helped to extend the 2D ferromagnetic material system and the related vdW heterostructures. Additionally, they helped to explore new physical properties and multiple device functions under environmental conditions. However, MBE has a long processing time, low mass productivity, and extremely high vacuum requirements.

### 2.2 Fabrication of vdW Heterostructures

#### 2.2.1 Dry Transfer

**Figure 4:**
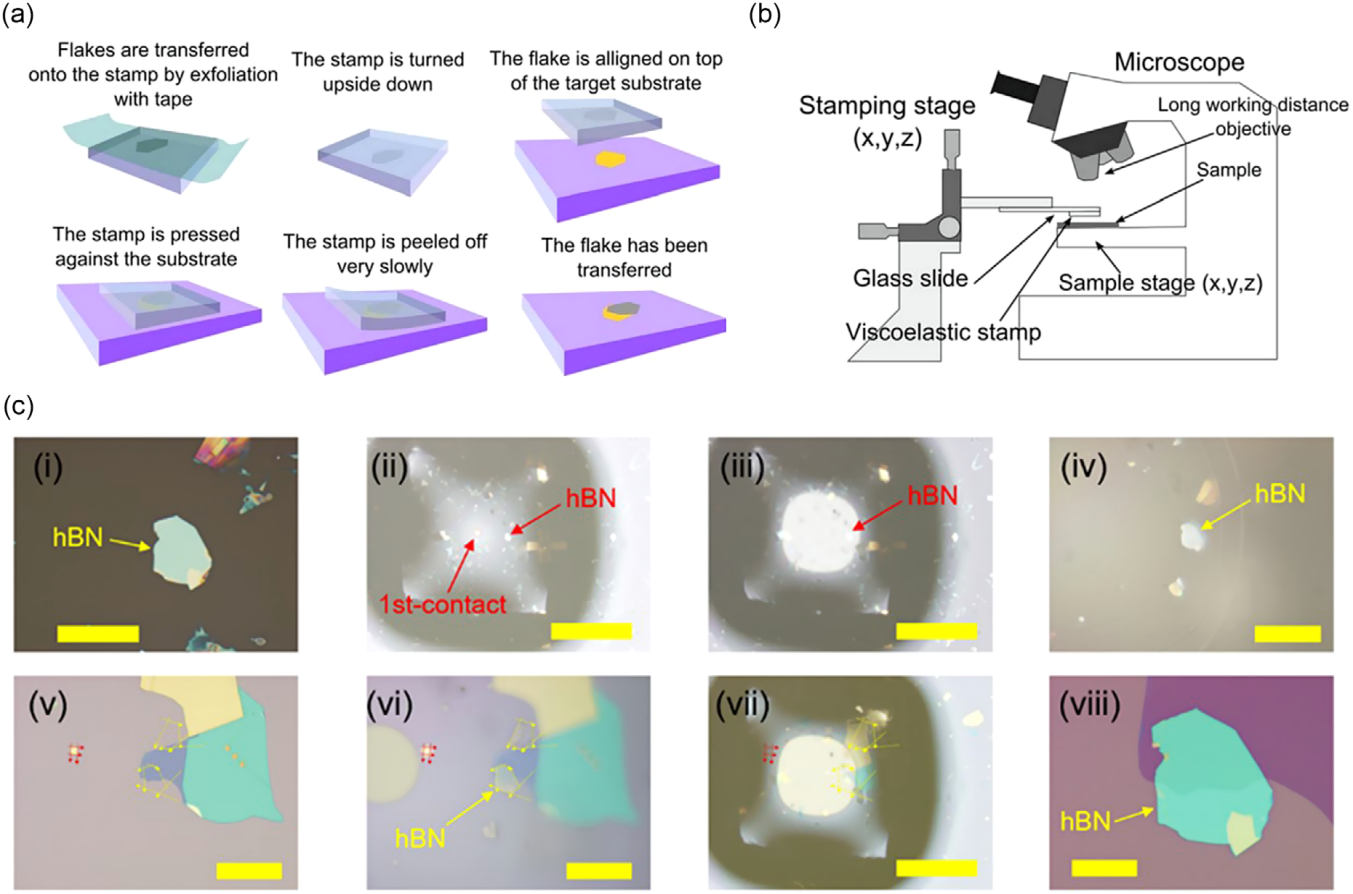
Setup and process of deterministic transfer. a) Demonstration of the steps in generating the viscoelastic stamp and the controlled transfer of an atomically thin flake to a designated position, such as another atomically thin flake. b) Conceptual representation of the experimental setup employed in the all‐dry transfer procedure. Reproduced with permission.^[^ ^68^ ^]^ Copyright 2014, IOP Publishing Ltd. c) Optical representation of the process flow during the transfer procedure. i) Initial state with h‐BN flake on a SiO_2_/Si substrate. ii) The stamp's center initiates contact with the substrate. iii) Formation of a circular contact area around the h‐BN flake. iv) The elevated h‐BN flake on the stamp surface. v) The presence of a Gr flake on the substrate. Yellow and red arrows indicate alignment markers. vi) The stamp, carrying the h‐BN flake, begins contacting the substrate with the Gr flake. vii) A circular contact area encompasses the Gr flake, creating an overlap between the h‐BN flake and the Gr flake. viii) Formation of the h‐BN/Gr stack. Scale bars: 20 μm in (viii), 50 μm in (i), 100 μm in (iv–vi), and 500 μm in (ii–vii), respectively. Reproduced with permission.^[^ ^71^ ^]^ Copyright 2020, American Chemical Society.

In 2006, the dry transfer is first devised to transfer monolayer 2D systems directly into heterostructures,^[^ ^67^ ^]^ which is a clean and fast procedure to transfer a few layers of 2DMs from one substrate to another. For some soft, flexible, and sticky organic polymers (called stamps), adhesion forces exist after contact with some solids on the substrate. There is a link between the separation rate and the adhesion between the substrate and the organic film when an external force is employed to separate them, and the exfoliation rate affects the adhesive strength and hence the transfer direction. The stacking of heterostructures may be completed,^[^ ^68^ ^]^ as shown in **Figure** **4**a, using this notion of moving samples across various substrates using a micromanipulation device (as shown in Figure 4b), which is the simplest idea of preparing heterostructures by an all‐dry method. Other approaches can be built on this foundation. One option is to utilize polycarbonate (PC) glue as the transfer medium.^[^ ^69^ ^]^ PC is more difficult to spin coat than other organic films, and only a second carrier may be used; only a second slide can be placed on the first PC to let the PC expand evenly and become flat, followed by quickly separating the two slides. There is also a method of preparing heterostructures using spin‐coated organic films.^[^ ^70^ ^]^ Takuya Iwasaki et al. proposed a bubble‐free transfer method for vdW heterostructures;^[^ ^71^ ^]^ it makes it simple to prepare viscoelastic imprints for the transfer process while preventing bubble formation by introducing contact angles and slow contact velocities between the sheets (Figure 4c).

#### 2.2.2 Wet Transfer

**Figure 5:**
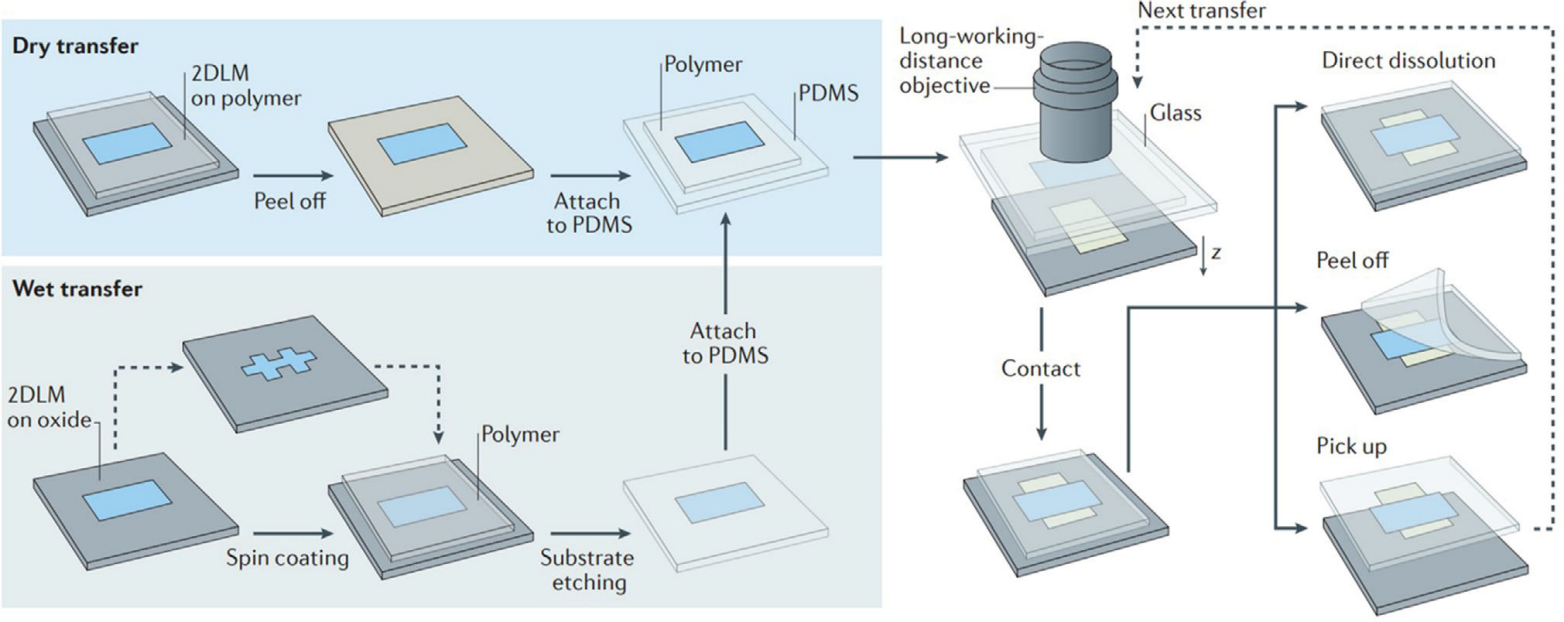
Conceptual depiction of the most recent alignment transfer techniques for integrating vdW heterostructures. Both wet and dry transfer methodologies are employed to attach the target sheet onto the stamp material. Subsequently, the stamp is affixed to a glass slide and situated within a transfer microscope. Micromanipulators enable precise alignment of sheets with the assistance of a long‐working‐distance objective lens. The polymer transfer stamp can be dissolved chemically, removed mechanically, or used to raise the entire stack for subsequent transfer steps. Reproduced by permission.^[^ ^75^ ^]^ Copyright 2016, Macmillan Publishers Limited.

Based on exfoliation method and media layer types, the wet transfer can be divided into polyvinyl alcohol (PVA) adsorption transfer method,^[^ ^72^ ^]^ polymethyl methacrylate (PMMA)‐assisted transfer method,^[^ ^73^ ^]^ and poly‐l‐lactic acid (PLLA) fast transfer method.^[^ ^74^ ^]^ Compared with dry transfer described above, the liquid must be injected into the exfoliation and transfer process (see **Figure** **5**).^[^ ^75^ ^]^ After transfer, the procedure of heterostructure stacking is similar to that of dry transfer.

#### 2.2.3 In Situ Growth Method

**Figure 6:**
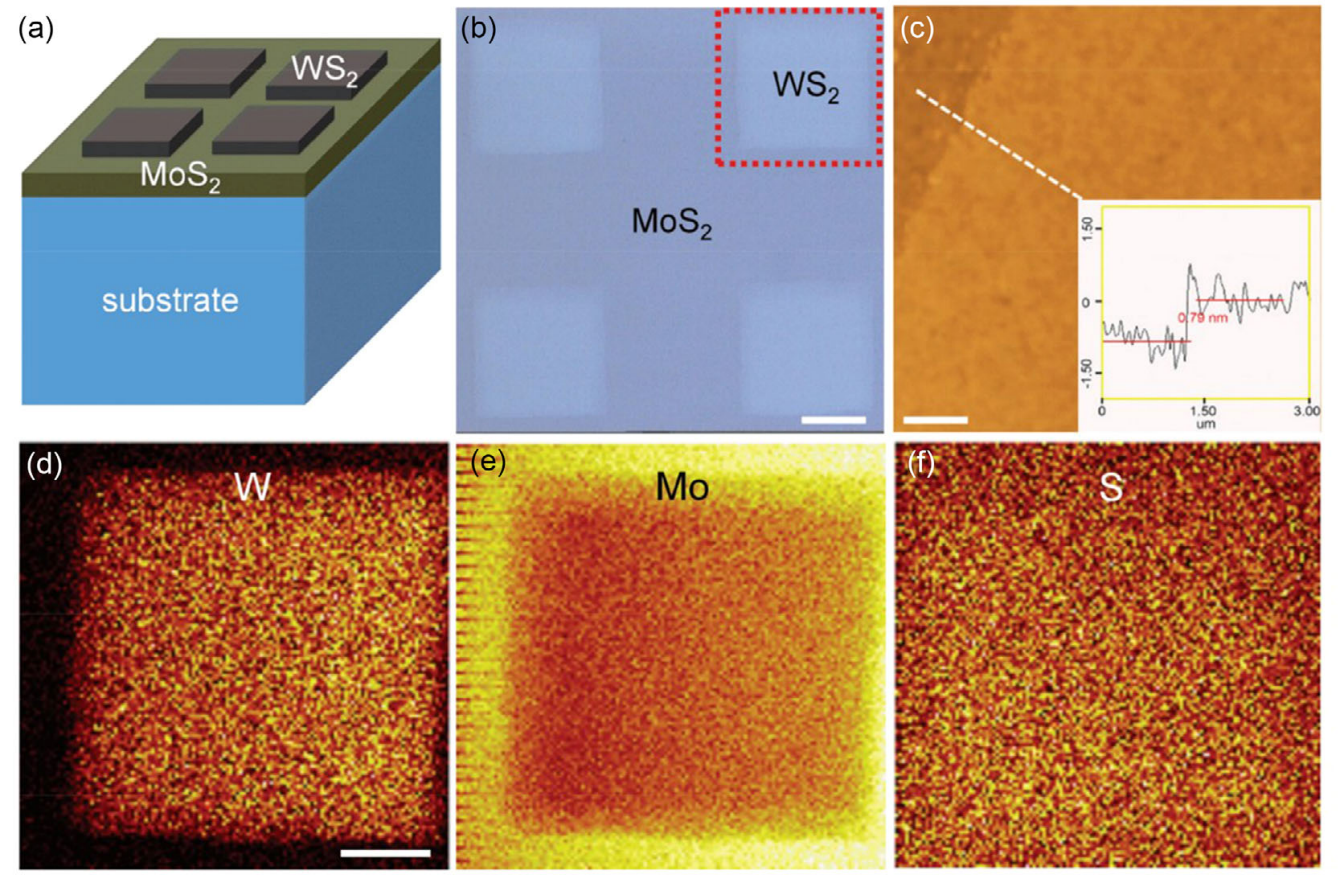
Patterned WS_2_/MoS_2_ monolayer heterostructure. a) Conceptual representation of layered WS_2_/MoS_2_ structures on a SiO_2_/Si substrate, emphasizing the patterned WS_2_ layer on the surface. b) Microscopic view of the layered WS_2_/MoS_2_ structures with patterning. Scale bar: 200 μm. c) AFM visualization of the heterostructure, including a height profile indicating the monolayer WS_2_'s thickness. Scale bar: 1 μm. d–f) Time of flight secondary ion mass spectrometry (TOF‐SIMS) elemental maps derived from the region outlined in (b), depicting the spatial distribution of W, Mo, and S in the WS_2_/MoS_2_ heterostructure. Scale bar: 100 μm. Reproduced with permission.^[^ ^79^ ^]^ Copyright 2018, Royal Society of ChemistryRoyal Society of Chemistry.

Su et al. reported an “in situ growth” synthesis technique for 2D/2D heterostructures.^[^ ^76^ ^]^ The production of 2D layered materials on the surface of another 2D layered material by one‐step growth or multistep transformation processes is referred to as the “in situ growth method.” Such distinct synthesis processes can provide different heterostructure interfaces and application potentials depending on certain principles. Li et al. created MoS_2_/PbS heterostructures by in situ growth to make tighter contact between MoS_2_ and PbS.^[^ ^77^ ^]^ The composites have greater light‐trapping ability to absorb light and separate electrons from holes, increasing the photocatalytic activity. In another work, Sneha Sinha et al. effectively produced large‐area MoS_2_/WS_2_ vdW heterostructures in situ and established those photodetectors with such structures that had outstanding photocurrent detection capability.^[^ ^78^ ^]^ Furthermore, Yang et al. used one‐step reactive sputtering to demonstrate this “in situ growth” strategy for large‐area 2D WS_2_/MoS_2_ heterostructures (as shown in **Figure** **6**),^[^ ^79^ ^]^ finding that interlayer vdW forces between vertical layers may be overcome by kinetically blasted atoms, resulting in a huge number of randomly oriented stacks with different twist angles.

#### 2.2.4 Ar^+^‐Ion‐Bombardment Assistant

**Figure 7:**
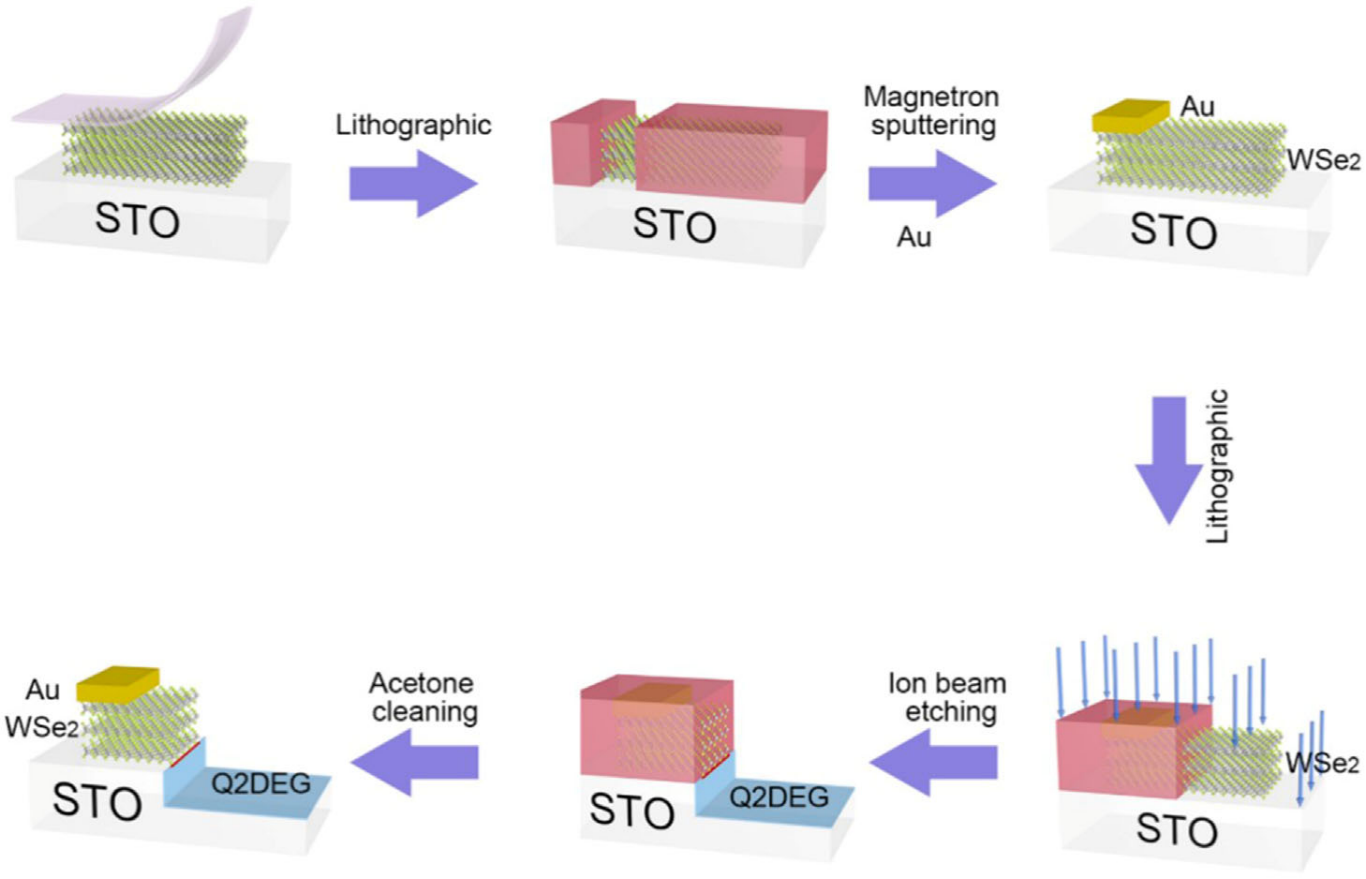
Diagram of the fabrication process for WSe_2_/Q2DEG heterostructures. Initially, few‐layered WSe2 is exfoliated onto the STO substrate using tape. The specimen is then covered with positive photoresist within a square area etched on one side of the WSe_2_ flake. Subsequently, an Au electrode is deposited onto the square area via magnetron sputtering, and the photoresist is eliminated using acetone. Afterward, the Au electrode is shielded by the photoresist, exposing half of the WSe_2_ flake to air. Ultimately, an Ar^+^‐ion beam is used to etch the sample's surface. Following a cleaning process with acetone, the WSe_2_/Q2DEG heterostructure is effectively fabricated. Reproduced figure with permission.^[^ ^23^ ^]^ Copyright 2021, American Physical Society.

Jiang et al. developed a practical and versatile Ar^+^‐ion‐bombardment assistant technique (AIBA) that establishes a lateral vdW p–n heterostructure between few‐layer WSe_2_ and a Q2DEG on the SrTiO_3_ (STO).^[^ ^23^ ^]^ One of the key applications of this technology is the creation of Q2DEG vdW heterostructures and 2DMs. **Figure** **7** depicts a schematic representation of the WSe_2_/Q2DEG heterostructure production process. From the bulk single crystal, a number of WSe_2_ layers were manually exfoliated and adhered to the STO substrate. The sample is coated with a positive photoresist and a square region etched on one side of the WSe_2_ sheet by photolithography. The gold electrodes were then magnetron sputtered into square regions and the photoresist was removed with acetone. The photoresist then covers the gold electrode, leaving half of the WSe_2_ sheet exposed to the air. The samples were then bombarded for 5–15 min with an Ar^+^‐ion beam at 200 V, 5 mA. During the etching procedure, a water‐cooled sample holder was used. Finally, the surface of the specimen was etched with Ar^+^ ion. WSe_2_/Q2DEG vdW heterostructures were successfully produced after acetone washing.

#### 2.2.5 Surface Planarization of VdW Heterostructures

The major issue with artificially stacked heterostructures is that bubbles and wrinkles occur on the surface due to the unevenness of the 2DMs, inadequate contact, or dirt on the surface. As a result, Rosenberger et al. pressed the heterostructure using the contact mode approach of AFM,^[^ ^80^ ^]^ by regulating the amplitude of the pressure at the needle tip when it makes contact with the sample.

**Figure 8:**
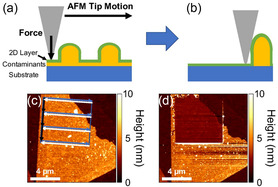
a,b) Schematic representations illustrating the fundamental concept of AFM flattening. Following mechanical transfer, contaminants, typically comprising a uniform layer and bubbles, accumulate between the 2D layer and the substrate, as shown in (a). The application of a normal load with the AFM tip ejects the contaminants from beneath the tip, establishing direct contact between the 2D layer and the substrate. Scanning the tip across the surface while maintaining a normal load results in the accumulation of contaminants into a pocket, creating a region free of contaminants, as depicted in (b). c) AFM topography image depicting CVD WSe_2_ mechanically transferred onto h‐BN. Arrows indicate the general scanning pattern used for flattening an area. Starting from the top left corner, the AFM tip scans right (blue arrows), left (white arrows), and then moves down between each scan line (black arrows). d) AFM topography after flattening a 6 μm × 6 μm section of the WSe_2_ sample. Reproduced with permission.^[^ ^80^ ^]^ Copyright 2018, American Chemical Society.

There are bubbles, wrinkles, and other irregularities between the sample and the substrate before the atomic force microscope (AFM) contact, as illustrated in **Figure** **8**a, and the contact between the sample and the substrate is incomplete and uneven. Scanning using the contact mode of AFM may eliminate air bubbles and wrinkles while also forcing the sample to contact the substrate, causing impurities to travel to the sample's edge (b). The AFM surface topography pictures of the material before and after scanning are shown in Figure 8c,d. The surface of the sample becomes flat after scanning, and the height of the scanning region is significantly lowered.

### 2.3 Mixed‐Dimensional Heterostructures

#### 2.3.1 0D/2D

**Figure 9:**
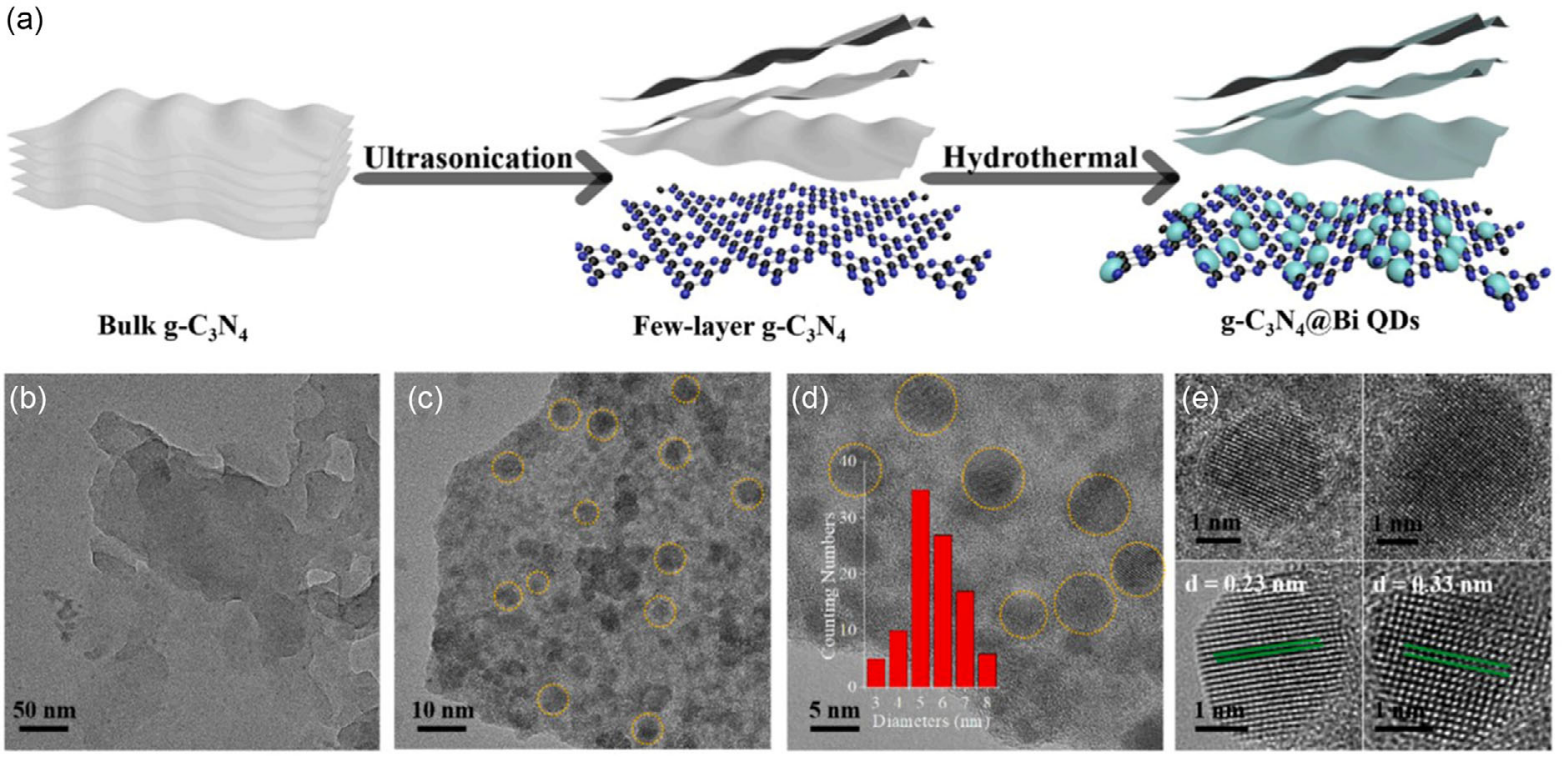
a) Schematic representation of the synthesis process for ultrathin g‐C_3_N_4_ NSs and g‐C_3_N_4_@Bi. b) Transmission electron microscopy (TEM) visualization of ultrathin g‐C_3_N_4_ NSs. c,d) TEM images of g‐C_3_N_4_@Bi at varying magnifications, with an inset in d) showing the diameter distribution of Bi QDs. e) HRTEM images of encapsulated Bi QDs. Reproduced with permission.^[^ ^84^ ^]^ Copyright 2021, Elsevier B.V.

By combining dimensionally adjustable semiconductor 0D quantum dots (QDs) with 2D layered materials, one form of vdW heterostructures has been created.^[^ ^81^ ^]^ This type of 0D–2D mixed‐dimensional vdW heterostructures (MVDWHs) has been found to offer various benefits, including lower Coulomb interactions, ease of manufacture, and fewer interface‐state limitations. The visual features of 2D layered materials, paired with their peculiar physical properties, can produce intriguing interfacial charge behaviors and drive device performance. Wu et al. constructed typical type I and type II electrical structures,^[^ ^82^ ^]^ respectively, using the band alignment procedure, and accomplished efficient band structure engineering of 0D–2D MVDWH, with the designed type II band alignment MvdWH‐based phototransistor (PFET‐II) device being more efficient than the type I band alignment MvdWH‐based phototransistor (PFET‐I) device. The device's performance is significantly improved, with a big optical gain and strong optical responsivity. Using in situ growth technique, Ye et al. created a new 0D/2D heterostructure of vanadate QDs/g‐C_3_N_4_ nanosheets (NSs) with good visible light catalytic activity,^[^ ^83^ ^]^ and this study provided a new strategy for producing functional materials from nanostructures of various sizes. As illustrated in **Figure** **9**, Zhang et al. developed 0D bismuth QDs (Bi QDs)/2D g‐C_3_N_4_ (g‐C_3_N_4_@Bi) heterostructures,^[^ ^84^ ^]^ which were synthesized and spun on an ITO‐coated PET substrate for use as a photodetector. Response and recovery times are quick, photocarrier pair absorption and transmission periods are shortened, and performance is improved.

#### 2.3.2 1D/2D

**Figure 10:**
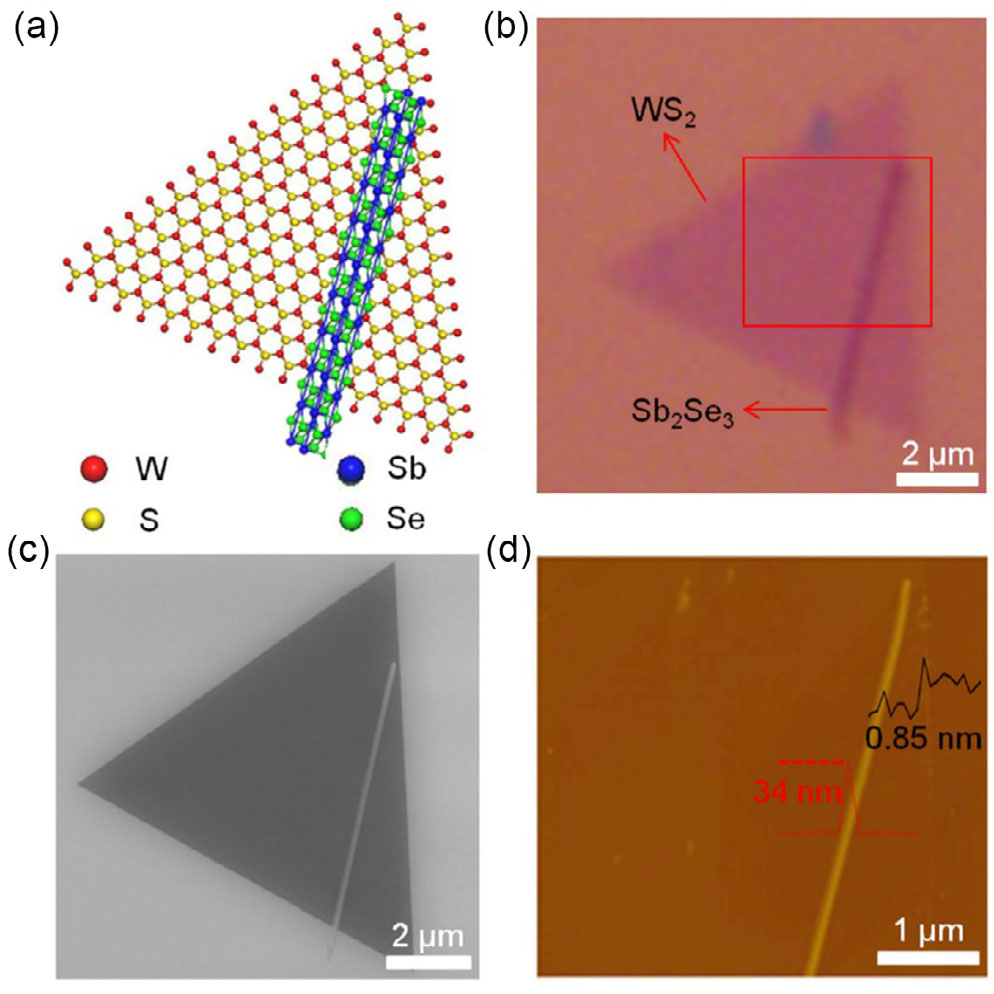
Illustration and morphological examination of the Sb_2_Se_3_/WS_2_ p–n heterostructure. a) Diagrammatic representation of the developed heterostructure, with W, S, Sb, and Se atoms represented by red, yellow, blue, and green spheres, respectively. b) Optical image of the Sb_2_Se_3_/WS_2_ p–n heterostructure. c) Scanning electron microscopy (SEM) visualization of the Sb_2_Se_3_/WS_2_ p–n heterostructure. d) AFM image of the region outlined by the red rectangle in (b). Reproduced by permission.^[^ ^86^ ^]^ Copyright 2019, Tsinghua University Press and Springer‐Verlag GmbH Germany, part of Springer Nature.

The use of a mix of 1D and 2D nanomaterials is a good method to improve performance, and the development of 1D/2D heterostructures can optimize the synergistic and multidimensional effects of 1D and 2DMs while also broadening their applications. Li et al. reported the one‐step growth of Bi_2_S_3_/MoS_2_ heterostructures and comprehensively explored the defect‐induced conucleus growth mechanism in 1D/2D heterostructures.^[^ ^85^ ^]^ The combined powder MoO_3_ and Bi_2_O_3_ is reacted at a high temperature of sulfur vapor (650 °C) in a CVD system with a SiO_2_/Si substrate to generate a 1D Bi_2_O_3_/2D MoS_2_ heterostructure. This work shows the conductivity is greatly improved, indicating that the produced 1D/2D heterostructures have good optoelectronic capabilities. Apart from the one‐step growth method, Sun et al. used a two‐step vapor‐phase growth process to create 1D Sb_2_Se_3_ NWs/2D WS_2_ nanosheet p–n heterostructures in addition to the one‐step method.^[^ ^86^ ^]^ A WS_2_ monolayer was first produced on a SiO_2_/Si substrate utilizing a special vapor deposition method. Following that, the generated WS_2_/SiO_2_/Si substrate was subsequently moved to a different quartz tube furnace to develop Sb_2_Se_3_ NWs, resulting in a 1D/2D heterostructure, as illustrated in **Figure** **10**. The two‐step vdW epitaxy process has three benefits: 1) it successfully avoids the fabrication of two‐material alloys, 2) it makes it easier to create a suitable contact interface, and 3) it allows for the growth of large‐area, high‐yield products. Moreover, He et al. created 1D/2D CdS/MoS_2_ composites for photocatalytic hydrogen generation by coating MoS_2_ nanosheets on CdS NWs.^[^ ^87^ ^]^

#### 2.3.3 2D/3D

2D/3D heterostructures offer unique advantages in photoelectronic, ferroelectricity, and thermoelectricity, where the photoelectronic properties of a material can be enhanced or altered by laminating a layer of 2DMs on top of a conventional 3D material.^[^ ^88^, ^89^, ^90^ ^]^ Such structures are commonly used in the design of FeFETs, where the 3D ferroelectric material affects the planar electrical transport properties of the 2DMs, which tend to result in ferroelectric behaviors such as hysteresis loops, piezoelectric response, and so on, in 2DMs that are not ferroelectric. Interestingly, Li et al. studied the electrical transport behavior of Gr/BaTiO_3,_^[^ ^90^ ^]^ and unlike the general out‐of‐plane electrical transport, they chose to study the vertical electrical transport behavior of Gr/BaTiO_3_ heterostructures. The *I–V* curves of the Gr/BaTiO_3_ heterostructures exhibit hysteresis behavior due to the effect of the iron electrode polarization and show different *I–V* behaviors at different polarization states of BFO. The *I–V* characteristics exhibit diode‐like behavior in the positive polarization state and reverse diode behavior in the negative polarization state. Additionally, the application of 2D/3D heterostructures demonstrates significant potential for photovoltaic (PV) applications.^[^ ^91^ ^]^ Constructing metal/semiconductor heterostructures provides a straightforward approach for creating PV devices. The interaction between a metal and a semiconductor featuring distinct work functions results in the establishment of a built‐in field. Opting for materials with significant variations in work functions enables the generation of a substantial PV voltage. Nonetheless, the formation of metal/semiconductor junctions tends to introduce interfacial states at the junction interface, potentially diminishing the photovoltage. Furthermore, the opaqueness of the metal cover layer hinders effective light absorption in the semiconductor, leading to suboptimal sunlight utilization and reduced conversion efficiency. To address these challenges, the incorporation of 2DMs with layered structures, such as Gr or TMDCs, onto 3D semiconductors emerges as an effective solution.^[^ ^92^, ^93^ ^]^

### 2.4 2DMs/Q2DEG Heterostructures

As 2DEG is generally easy to obtain vdW heterostructures, Jiang et al. employed the AIBA method to produce 2DMs/2DEG layers.^[^ ^94^ ^]^ All of the generated samples showed distinct heterointerfaces and excellent rectification performance, confirming the creation of p–n junction and Schottky junction. Furthermore, optoelectronic studies reveal that these 2DEG‐based heterostructures have exceptional photoconductivity at room temperature. Similarly, they used the AIBA method to create a high carrier density Q2DEG on a KTO substrate, whose resistance exhibits a linear relationship with time and remains constant for a long period in a stable dark environment,^[^ ^95^ ^]^ indicating its potential use as a timer. Apart from this, they used the AIBA method to produce vdW WSe_2_/2DEG heterostructures on KTO substrates.^[^ ^96^ ^]^ At room‐temperature bias, a high‐performance PV response was discovered when exposed to visible light. The current abruptly shifted by more than four orders of magnitude when the light came on. Because of the PV effect, the device provides a self‐powered photocurrent even without a bias voltage.

### 2.5 Summary

In the earlier article, we discussed the synthesis of 2DMs, the fabrication of vdW heterostructures, and vdW heterostructures with different dimensions. We summarized the advantages and drawbacks of these strategies in **Table** **6** and **7**. The characteristics of the various methods and structures were discussed in detail; however, there are still some problems. Mechanical exfoliation is one of the most classical strategies. The world was shocked when Gr was discovered on tape, and it seemed that we were just a piece of tape away from being the most advanced scientists. Although extremely simple, this strategy is extremely versatile, and to date more than 100 materials have been shown to be accessible by mechanical exfoliation. This minimalist strategy offers great opportunities for researchers to obtain high‐quality layered materials without the need for expensive equipment and raw materials; a roll of tape can help them. However, this strategy faces an inescapable problem: the yield of mechanical exfoliation is too low, and the resulting layer materials are too small in size, often on the order of micrometers. Although metal‐assisted exfoliation methods have been proposed, the yields and pass rates of this method are strongly dependent on the operator's skill and the quality of the raw material, and it is not possible to consistently produce large‐area, high‐quality 2DMs. Small size is not a problem for researchers, but it is clearly unsuitable for large‐scale industrial production, so mechanical exfoliation can only be used in the laboratory and be applied for state‐of‐the‐art exploratory work.

**Table 6:** Advantages and drawbacks of different methods of fabrication of vdW heterostructures

Method	Advantages	Drawbacks
Dry transfer	a) Reduced contamination risk b) Enhanced precision c) Simplified processing	a) Limited compatibility b) Mechanical stress: c) Reduced adhesion: d) Scale‐up challenges
Wet transfer	a) High material compatibility b) Superior adhesion c) Mitigation of mechanical stress d) Applicability to thin‐film materials	a) Potential introduction of contaminants b) Complex processing procedures c) Limitations on applicability to specialized materials d) Impact on thin‐film quality
In situ growth method	a) Superior crystal quality b) Control over interface quality c) Consistent manufacturing process d) Energy efficiency	a) Material selection constraints b) Growth condition limitations c) Complexity of the process d) Surface treatment challenges
Ar^+^‐ion‐bombardment assistant	a) Surface cleaning and activation b) Improved quality of heterostructures: c) Control over heterostructure morphology d) Enhanced adhesion of heterostructures	a) Potential introduction of argon: b) Potential introduction of mechanical stress: c) Equipment complexity: d) Not applicable to all materials

**Table 7:** Advantages and drawbacks of different methods of fabrication of 2DMs

Method	Advantages	Drawbacks
Mechanical exfoliation	a) Monolayer preparation b) High‐quality crystals c) Simple equipment d) Wide applicability	a) Low yield b) Introduction of impurities c) Damaging exfoliation d) Uncontrollable layering
CVD	a) High yield b) Uniformity c) Controllability d) Wide applicability	a) High‐temperature requirements b) Equipment complexity c) Emission treatment d) Uneven coating quality
MBE	a) High purity: b) Atomic layer control: c) Precise composition control: d) No need for seed crystals:	a) Slow growth rate b) Equipment complexity c) Limitations d) Energy consumption

The CVD method is a promising approach for obtaining large‐area 2DMs and can also be used for the growth of transverse vdW heterostructures. Compared to the simplicity of mechanical exfoliation, 2DMs prepared through CVD methods often exhibit an increased presence of defects, thereby influencing their electrical, optical, mechanical, and chemical properties. These defects contribute to a diminished electrical conductivity and impact the carrier mobility of the material. They induce alterations in the energy band structure, introducing additional electronic defect energy levels and consequently affecting optical absorption and emission behavior. Additionally, defects lead to lattice distortions and alterations in elastic constants, influencing mechanical properties such as elasticity modulus and hardness of the 2DMs. Certain defects may render the material more susceptible to environmental factors like oxygen, water, or other chemicals, thereby impacting its chemical stability. Meanwhile, CVD growth of high‐quality 2DMs requires more considerations such as precursors, growth temperature, catalysts, and substrates. Although CVD has been shown to be useful for the growth of a wide variety of 2DMs, the mechanisms involved in the CVD growth process are not fully understood due to multiple influencing factors. Precursors are derived from the evaporation of powders at elevated temperatures, which facilitates spontaneous nucleation during temperature ramps preceding and following the growth process, and while the introduction of catalysts increases the overall reaction rate and facilitates the production of large‐area 2DMs, it is important to consider whether residual catalysts and possible doping may affect the stability and pristine properties of the products. The products of CVD growth often involve strong coupling to the substrate, which may affect the physical properties. This complicates further processing of the material. MBE methods appear to be more delicate and advanced than mechanical exfoliation and CVD methods, which have extremely high vacuum requirements. This also leads to high maintenance and equipment costs, as well as high‐temperature control requirements, with low temperatures leading to low quality and high temperatures leading to reevaporation of the product. Even with the high investment, the yield of MBE method is still too low to be a generalized strategy.

Overall, the methods for making vdW heterostructures can be categorized into two approaches: Top‐down deterministic assembly and bottom‐up synthesis. The top‐down approach involves the mechanical stacking of 2DMs, which are usually separated from large pieces of layered material by peeling. Bottom‐up synthesis is based on a crystal growth process in which successive assemblies are added by providing a suitable gas‐phase or gas‐phase precursor at elevated temperatures. Wet transfer and dry transfer mentioned above are top‐down methods where most of the material transfer is done in air or liquid and hence, the inevitability of adsorbed molecules on the surface. The presence of molecules caught between the layered crystal and the substrate leads to enhanced separation and diminished dipole interactions. During dry transfer, mechanical deformation of the material is unavoidable due to direct contact and pressure between the PDMS and the material being transferred. h‐BN has been used as an intermediate to minimize the direct contact between the material and the PDMS in recent studies. The in situ growth method is a bottom‐up synthesis, where 2DMs can be directly grown on another 2DM by CVD method; this method produces heterostructures also with higher quality and cleaner interfaces compared to mechanical transfer. However, the presence of lattice matching and the different growth temperatures of different 2DMs limit the generation of specific heterostructures. The Ar^+^‐ion bombardment‐assisted method is extremely well suited for the fabrication of transverse heterostructures, where photolithography and Ar^+^‐ion bombardment produce extremely regular junction interfaces, allowing for the fabrication of high‐quality heterostructures. However, this method is currently only applicable to the production of 2DMs/Q2DEG heterostructures.

When combining 2DMs of different substances, several issues may arise. Some of these issues include the electronic affinity between different materials and differences in bandgap structures, which can lead to charge transfer at the interface of heterostructures. This may impact the electronic transport properties and device performance. 2DMs of distinct substances may undergo chemical reactions upon contact, forming new compounds. This could result in interface instability and material degradation. Some 2DMs may be more prone to damage during mechanical exfoliation or transfer processes. This may lead to the presence of defects in the heterostructure, thereby affecting performance. Inconsistent growth directions of 2DMs may result in rotation or misalignment in heterostructures, potentially having adverse effects on electronic transport and device performance. To address these challenges, researchers typically employ the following strategies in scientific investigations: lattice matching, interface engineering, surface modification, optimization of exfoliation methods, and directed growth. Here are some possible examples.

When combining different 2DMs, bubbles and folds are often created in the heterostructure due to various reasons, and using the probe tip of an AFM to lightly scratch across the heterostructure surface proves to be an effective approach in enhancing the quality of the heterostructure. Nanoetching is created when the force of the AFM tip is increased, and this method can be used to finely cut 2DMs in order to remove defect locations present in the heterostructure of 2DMs. Similarly in dry transfer, the 2DMs can be partially contacted to the substrate and partially contacted to the PDMS, and then the triaxial rotating platform can be adjusted to flatten the 2DMs, a method derived from the process of making twisted angle Gr. The AIBA method is also suitable for solving problems that may arise when combining 2DMs. It can be used to produce more regular heterostructures without introducing additional chemicals.

## 3 Photoelectric Properties

Recently, 2DMs vdW heterostructures have attracted extensive interest due to their distinctive photoelectric properties. Because there is no need to consider lattice matching, any 2DMs can be combined by stacking to achieve excellent optical properties.^[^ ^21^, ^22^, ^24^, ^97^, ^98^, ^99^, ^100^ ^]^ Extensive literatures show that the photoelectric properties of vdW heterostructures are far superior to those of single 2DMs, such as high carrier mobility,^[^ ^101^ ^]^ fast photoelectric response,^[^ ^102^ ^]^ excellent light absorption,^[^ ^103^ ^]^ persistent photoconductivity,^[^ ^104^ ^]^ and exceptional PV properties.^[^ ^105^ ^]^

Through the construction of vdW heterostructure devices, advantages over single‐material systems can be achieved in the field of photoelectronic. VdW heterostructures enable the combination of different 2DMs, each possessing distinct characteristics. This tunability allows for the optimization of devices to meet specific application requirements, conferring multifunctionality in adjusting photoelectronic responses. The use of various materials in heterostructures may lead to improved device performance, encompassing enhanced charge transport, reduced recombination losses, and overall efficiency improvements compared to single‐material systems.

VdW heterostructures typically consist of 2DMs, exhibiting unique low‐dimensional effects such as quantum confinement. These effects can be harnessed to enhance light response and introduce novel functionalities in photoelectronic devices. The flexibility in designing vdW heterostructures enables the creation of intricate device architectures. This flexibility facilitates the customization of devices based on specific requirements, such as sensitivity to particular wavelengths or response times, showcasing the potential for tailored applications.

### 3.1 Photoconductive Effects

Photoelectric effects tend to arise when vdW heterostructures interact with light and electricity.^[^ ^50^, ^106^, ^107^ ^]^ With a material irradiated by light, electron–hole pairs will be created, and their conductivity will be enhanced. Based on this basic principle, a variety of heterostructures have been designed to fabricate advanced optical devices such as infrared detectors, photoelectric storage and memory devices, and solar cells. By applying a reverse bias voltage to the heterostructures, a simple structure for infrared detection is formed. The detection gain of this structure is positively correlated with the minority carrier lifetime excited by the optical signal, but the long minority carrier lifetime is negatively correlated with the response speed, which requires a balance between speed and gain. For memory devices, it is necessary to prevent the regroup of electron–hole pairs to form persistent photoconductivity in vdW heterostructures, thereby enabling information storage. External quantum efficiency (EQE) is an important parameter for these devices. EQE is the proportion of collected electrons in the device relative to the total incident photons. The formula of EQE = *I*_ph_ */qφ*_in_, where, *q* denotes the electron charge, *I*_ph_ signifies the measured photocurrent, and *φ*_in_ is the incoming photon flux.

#### 3.1.1 Infrared Photodetectors

Infrared photodetectors (IRPDs) is a device that converts the input infrared radiation signal into other types of signal outputs. In 1800, William Herschel discovered infrared radiation using a thermometer, one of the first heat‐sensitive infrared detectors.^[^ ^108^ ^]^ Around the 1940s, photon‐type detectors made using the photoelectric effect of semiconductors had a huge impact on infrared technology.^[^ ^109^ ^]^ Therefore, infrared detectors with different response wavelengths are realized. Now, infrared detectors become the important equipment in key fields such as military, medicine, industry, and agriculture. For higher standard requirements, IRPDs are developing toward miniaturization, high sensitivity, and low cost.

Due to the unique structure and properties of 2DMs, they can be flexibly stacked into heterostructures through vdW forces without considering the problem of lattice matching, thus showing great potential in infrared detection applications. Traditional materials for IRPDs, such as HgCdTe,^[^ ^110^ ^]^ InSb,^[^ ^111^ ^]^ InGaAs,^[^ ^112^ ^]^ etc., often require low‐temperature environment.^[^ ^113^ ^]^ However, a significant number of IRPDs based on traditional materials are costly and unsafe, causing their use in military rather than civilian applications. In contrast with traditional materials, the unique tunable bandgap and flexible material stacking properties of 2D vdW heterostructures exhibit advantages over conventional materials. To respond to infrared radiation, the materials should have a narrow bandgap, such as PdSe_2,_^[^ ^114^ ^]^ PtSe_2_,^[^ ^115^ ^]^ MoS_2,_^[^ ^21^ ^]^ Gr,^[^ ^22^ ^]^ etc. When irradiated by infrared light, photons are absorbed by these materials, resulting in electron–hole pairs. Some meaningful 2D or mixed‐dimensional heterostructures have been developed and exhibit excellent performance.

**Figure 11:**
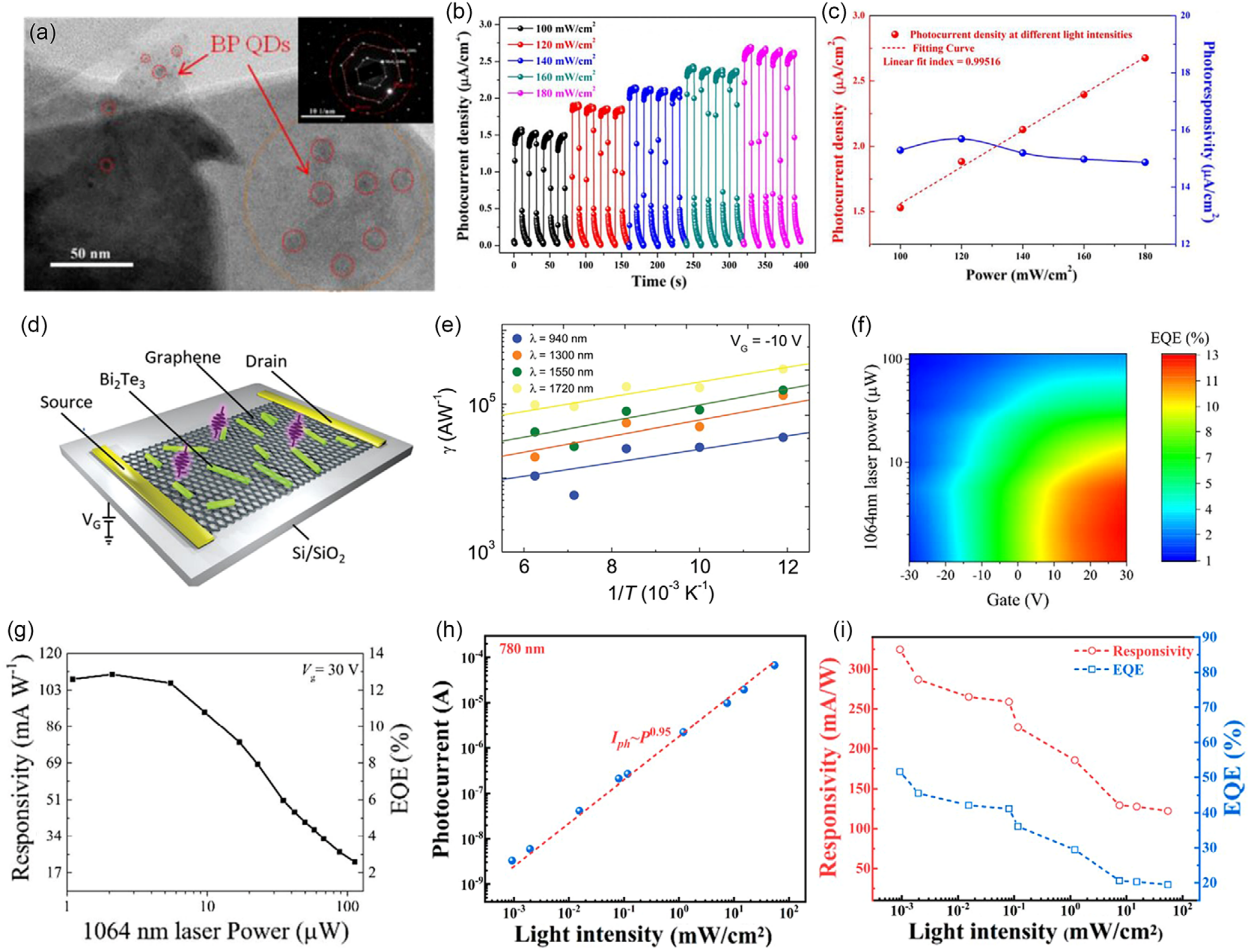
a) TEM image depicting the heterojunction between BP QDs and MoS_2_. The inset in (a) displays the selected‐area electron diffraction pattern within the circled region. b) Photocurrent density performance of the photo‐electrochemical‐type (PEC‐type) photodetector based on BP QDs/MoS_2_ composites in 1 m KOH electrolyte, measured at different light intensities (100, 120, 140, 160, and 180 mW cm^−2^) and 0 V bias. c) Fitting curve of the photocurrent and calculated responsivity of the PEC‐type photodetector using BP QDs/MoS_2_ composites. Reproduced with permission.^[^ ^116^ ^]^ Copyright 2020, Elsevier Ltd. d) Graphical representation of the Gr/Bi_2_Te_3_ NW heterostructures. e) Temperature‐dependent responsivity observed for various wavelengths (940, 1300, 1550, and 1720 nm) in the near‐infrared spectrum. The responsivity decreases as the temperature increases. Dashed lines indicate fits based on Arrhenius‐type activated behavior. Reproduced with permission.^[^ ^25^ ^]^ Copyright 2019, Royal Society of Chemistry. f) Back gate voltage and incident laser power influence EQE. g) The dependence of photoresponsivity and EQE on incident light power, maintaining the back gate voltage at 30 V. Photoresponsivity and EQE peak at 110 mA W^−1^ and 12.9%, respectively, for incident power below 5 μW. Reproduced with permission.^[^ ^117^ ^]^ Copyright 2017, American Chemical Society. h) Dependence of photocurrent on light intensity. i) Device's computed responsivity (R) and EQE at different light intensities. Reproduced with permission.^[^ ^118^ ^]^ Copyright 2021, American Chemical Society.

QDs have excellent weak light absorption capabilities due to their surface effects and quantum confinement. By forming a heterostructure between QDs and 2DMs, the electron transfer speed can be increased and the recombination of electron–hole pairs can be suppressed. Qiao et al.^[^ ^116^ ^]^ reported BPQDs/MoS_2_ heterostructures (**Figure** **11**a–c). Preparation of BPQDs by liquid‐phase exfoliation method combined with probe sonication and water bath sonication and then a photoelectrochemical photodetector based on BPQDs‐MoS_2_ heterostructures was constructed. The photoresponsivity of the BPQDs/MoS_2_ device can reach 15.7 μA W^−1^, and the specific detection rate is 3.7 × 10^−7^ Jones under 0 bias voltage and the irradiation intensity of 120 mW cm^−2^.

**Figure 12:**
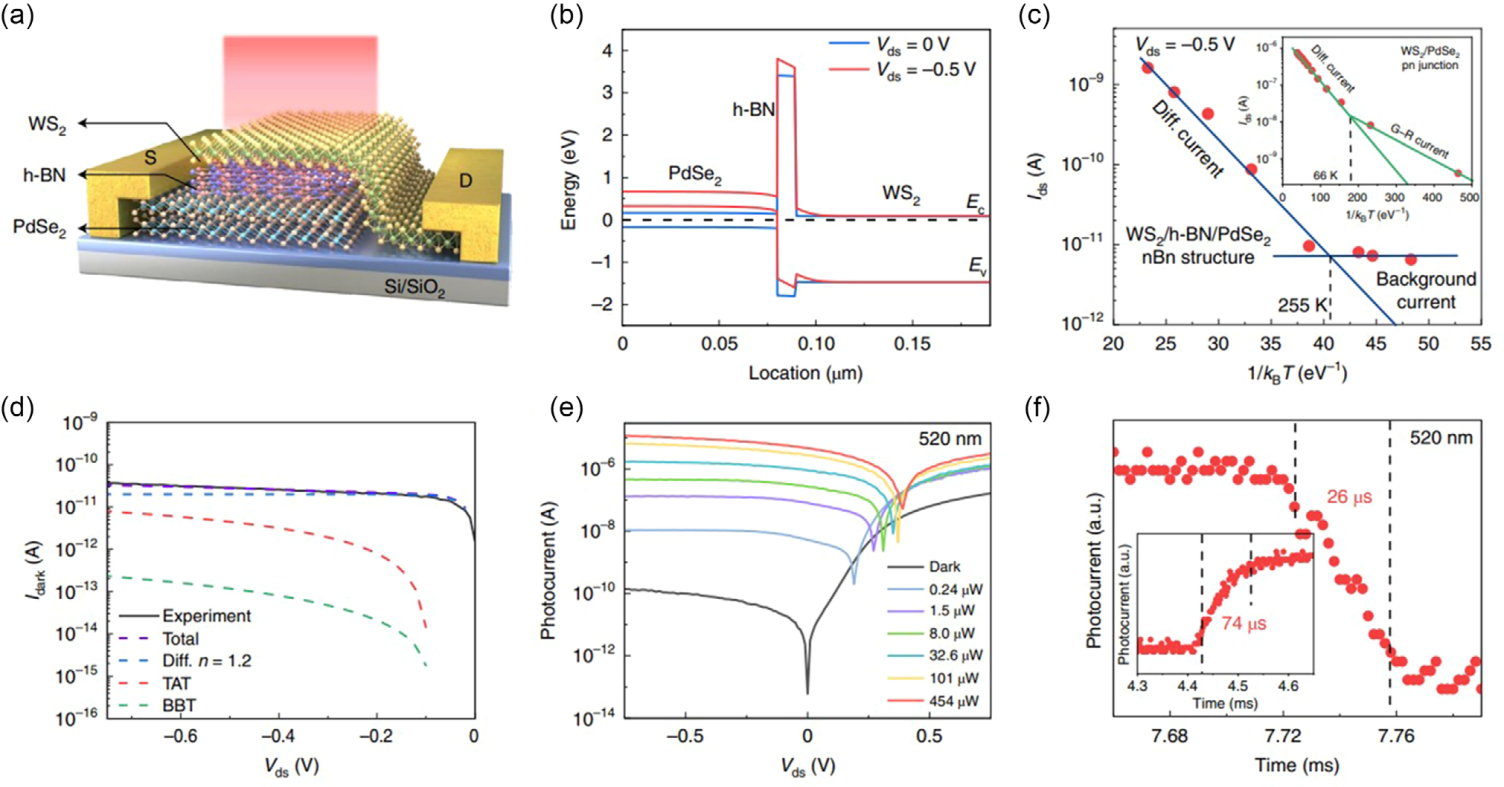
Attributes of room‐temperature nBn vdW unipolar barrier photodetectors in optoelectronics. a) Schematic depiction of the WS_2_/h‐BN/PdSe_2_ nBn vdW unipolar barrier photodetector. S denotes source; D indicates drain. b) Simulated band diagrams of the device under various source–drain bias (*V*_ds_) conditions. WS_2_, h‐BN, and PdSe_2_ flakes function as the absorber, barrier, and contact layer, respectively. c) Arrhenius graph illustrating the current behavior of the nBn vdW unipolar barrier device, showcasing a high‐operating‐temperature feature. Inset: Arrhenius graph displaying the dark current of the PdSe_2_/WS_2_ junction at a bias of −0.5 V. d) Components of the dark current in the nBn unipolar barrier photodetector. e) Output characteristic curves of the nBn vdW unipolar barrier device under 520 nm laser illumination with increasing powers. f) Response time of the device under 520 nm laser illumination at *V*_ds_ = −0.5 V. The corresponding *τ*_rising_ and *τ*_falling_ are 74 and 26 μs, respectively. a.u. represents arbitrary units. Reproduced with permission.^[^ ^20^ ^]^ Copyright 2021, Springer Nature Limited.

2D/1D heterostructures for infrared detection are still less studied; since the diameter of the NWs is much smaller than the carrier diffusion length, the internal quantum efficiency (IQE) can be close to 100%,^[^ ^32^ ^]^ and fast response speed and high quantum efficiency can be simultaneously achieved in IRPDs. Therefore, the use of NWs integrated with 2DMs can significantly improve the sensitivity to infrared radiation. This was also demonstrated by the study of Saurav et al.,^[^ ^25^ ^]^ who constructed Gr/Bi_2_Te_3_ NWs heterostructures by sensitizing Gr with Bi_2_Te_3_ NWs (Figure 11d, e). These devices show ultrahigh sensitivity of ≈10^6 ^A W^−1^ under light power from 940 to 1720 nm. The noise equivalent power of these devices can be up to ≈10^−18^ W Hz^−1/2^ and specific detectivity of these devices can be up to ≈10^11^ Jones. Due to the unique properties of 2D/2D heterostructures, materials with different properties can be flexibly stacked together through vdW interaction according to the requirements of different application scenarios, thereby generating a new combination of properties and functions that meet people's expectations. At present, 2D/2D vdW heterostructures can be prepared by CVD, mechanical transfer, and other methods. Zhang et al.^[^ ^117^ ^]^ fabricated Gr/MoTe_2_/Gr vertical vdW heterostructures on SiO_2_/*p*+‐Si substrates by a site‐controllable transfer method (Figure 11f, g), the device exhibits superior performance, photoresponsivity (≈110 mA W^−1^ at 1064 nm and 205 mA W^−1^ at 1473 nm), and EQE (≈12.9% at 1064 nm and ≈53.8% at 1473 nm). The device consists of a 140 nm‐thick MoTe_2_ flake sandwiched between two sheets of Gr, the vdW heterostructures are found to have a uniform photoresponse region by scanning photocurrent microscopy, and two Schottky barriers between MoTe_2_ and Gr lead to photocurrent generation. The photoresponsivity and EQE of IRPDs can be modulated by changing the thickness of MoTe_2_ flakes. Wu et al.^[^ ^118^ ^]^ reported PdSe_2_ (2D)/CdTe (3D) MVDWHs that operate at room temperature (Figure 11h). The photodetector has a response speed of 70 ns, a specific detectivity of 3.3 × 10^12^ Jones, a responsivity of 324.7 mA W^−1^, and a polarization sensitivity of 4.4 to polarized infrared light signals. Furthermore, 2D/3D heterostructures have improved light absorption and have been extensively studied due to their fast charge transfer and simple fabrication method. Unipolar barrier structures can suppress dark current in photodetectors to improve detector performance.^[^ ^119^ ^]^ However, due to the existence of lattice matching, using traditional materials to design unipolar barrier structures is difficult, and the advent of 2DMs makes it easy to construct. Chen et al.^[^ ^20^ ^]^ demonstrated band‐engineered vdW heterostructures that can be used to build unipolar barrier photodetectors for visible light and midwavelength infrared. They fabricated nBn‐type heterostructures based on WS_2_/h‐BN/PdSe_2_ and pBp‐type heterostructures based on BP/MoS_2_/Gr; barrier layer allows photocurrent to flow unimpeded and blocks dark current. NBn WS_2_/h‐BN/PdSe_2_ photodetector showed a dark current of 15 pA and a larger photocurrent of 20 μA. Under 520 nm laser irradiation, the light on–off ratio is 10^6^, and the room‐temperature specific detectivity is 2.7 × 10^12 ^cm Hz^1/2^ W^−1^. The room‐temperature peak specific detectivity of pBp BP/MoS_2_/Gr MWIR photodetector under blackbody radiation is 2.3 × 10^10 ^cm Hz^1/2 ^W^−1^, due to the natural sensitivity of the BP layer to blackbody source polarization; the dichroic ratio exceeds 4.9. **Figure** **12** shows the photoelectric properties of the nBn unipolar barrier infrared detector and its comparison with other IRPDs.

Although great progress has been made in IRPDs based on vdW heterostructures; there are still some problems to be solved. First, it is difficult to keep balance between fast response speed and high photoresponsivity. High photoresponsivity requires a long carrier lifetime, and a long carrier lifetime means long relaxation time, which reduces the response speed. Second, the synthesis efficiency of vdW heterostructures is low and can only be applied to laboratory research at present.

#### 3.1.2 Photoelectric Storage and Memory

Information storage has always been a key technique in modern times. In recent years, the processing speed of the CPU has increased exponentially, limited by the inherent properties of materials. The memory based on traditional materials has approached the theoretical limit, and the development of storage technology has gradually failed to match the development of information systems. Therefore, novel photoelectric storage devices based on 2DMs vdW heterostructures have attracted great interest.

**Figure 13:**
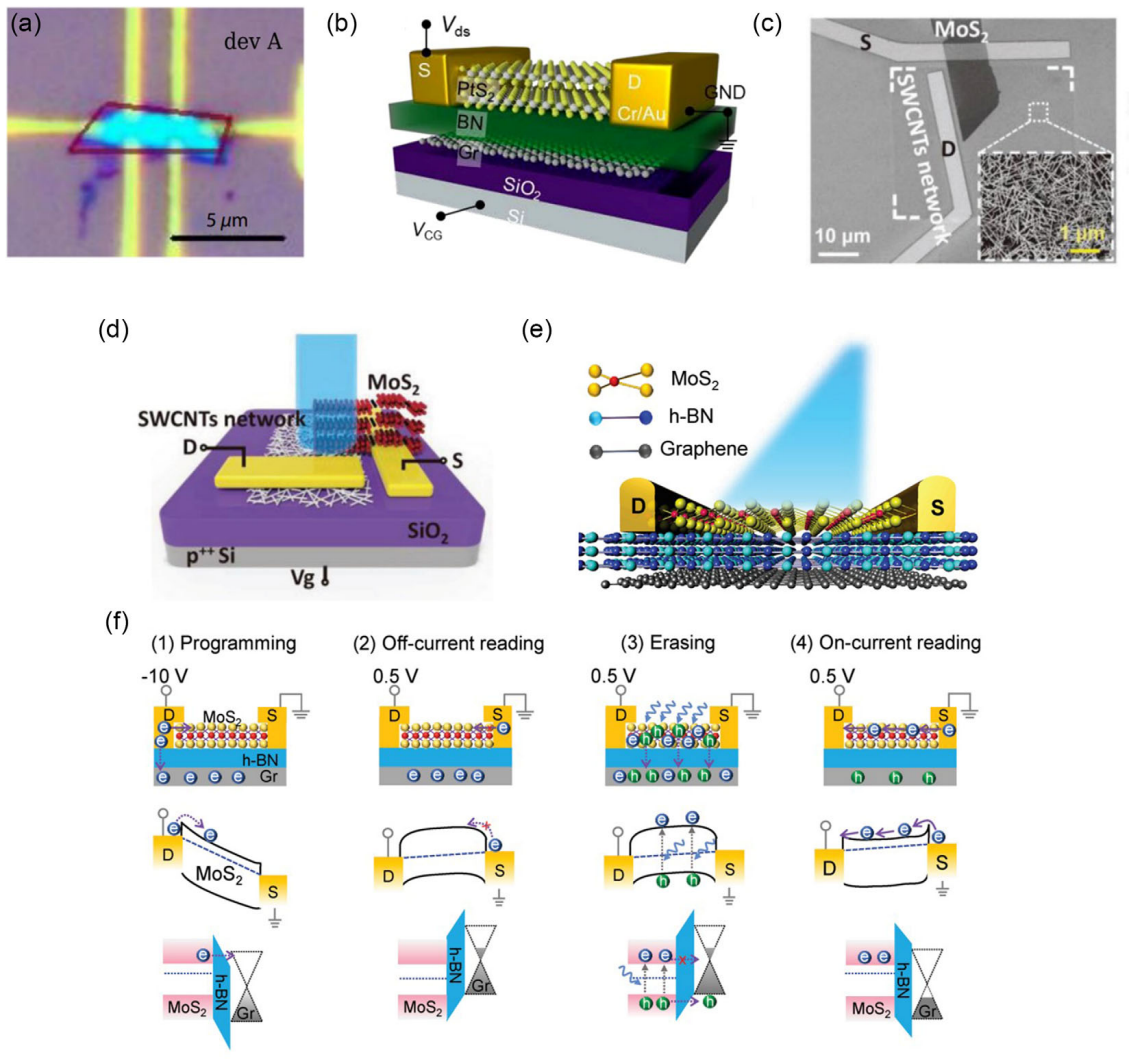
a) Optical picture of the Gr/WS_2_ device (dev A). The Gr area, acting as the measurement channel, is outlined in red. The scale is set at 5 μm. Reprinted figure with permission.^[^ ^120^ ^]^ Copyright 2020, American Physical Society. b) Diagram of the device setup on SiO_2_/Si substrate, with the back Si functioning as a control gate for memory. Reproduced with permission.^[^ ^121^ ^]^ Copyright 2020, Royal Society of Chemistry. c) SEM image and d) schematic representation of the MoS_2_/SWCNT network heterostructure on a 300 nm SiO_2_/Si substrate. The inset in (c) illustrates the morphology and density of the SWCNT network. Scales are set at 10 and 1 μm, respectively. Reproduced with permission.^[^ ^98^ ^]^ Copyright 2018, John Wiley & Sons; Wiley‐VCH Verlag GmbH & Co. KGaA. e) Schematic representation of an optical memory device utilizing the floating gate FET within the MoS_2_/h‐BN/Gr heterostructure. f) Illustration of the optical memory device operation (upper panels) and the corresponding energy band diagrams in the lateral (middle) and vertical (bottom) directions for: 1) programming, 2) off‐current reading, 3) erasing, and 4) on‐current reading. Reproduced with permission.^[^ ^122^ ^]^ John Wiley & Sons. Copyright 2018, Wiley‐VCH Verlag GmbH & Co. KGaA.

VdW heterostructures exhibit some excellent properties in developing photoelectric memory devices, such as high program/erase speed, fast response, multibit storage, etc. Sreemanta Mitra et al.^[^ ^120^ ^]^ reported Gr/WS_2_ vdW hybrid heterostructures for photoelectric storage device applications (**Figure** **13**a). As a result of the photogating mechanism, excitons under photoexcitation dissociate and holes are subsequently trapped in the WS_2_ surface, followed by electron transfer to Gr, resulting in gate‐tunable persistent photoconductivity. The recombination of electron–hole pairs has always been a key mechanism affecting the information storage performance of photoelectric memories. Chen et al.^[^ ^121^ ^]^ designed a photoelectric memory based on asymmetric hot‐carrier tunneling vdW heterostructures with PtS_2_/h‐BN/Gr (Figure 13b). PtS_2_ has a large electron affinity of about 4.8 eV, so when integrated with Gr, a highly asymmetric hot‐carrier tunneling barrier is created for the carriers of the h‐BN layer, enabling their efficient separation. These results are more than 1000 s of stable storage and a reliable optical program of the memory states up to 74 states.

Fast program/erase operation is another key feature of photoelectric storage devices. Yang et al.^[^ ^98^ ^]^ proposed MoS_2_/single‐walled carbon nanotubes (SWCNTs) network hybrid dimensional vdW heterostructures (Figure 13c, d). This structure has the potential to be applied to high‐performance optical memory, recording program/erase speed reaches ≈32/0.4 ms, high program/erase ratio (≈10^6^), and simple program/erase operation. When receiving an optical signal, MoS_2_ generates a good deal of electron–hole pairs by absorbing photon energy, and then the photogenerated holes in MoS_2_ are transported into the SWCNT network under the action of negative electric field force. Photogenerated holes cannot get away from the SWCNT network due to the steady charge traps in the SWCNT network. This prevents the regroup of photogenerated electron–hole pairs, thereby accumulating a good deal of photogenerated electrons in the MoS_2_ layer. Photoinduced memory devices remain conductive in the dark, resulting in states that store optical signals. When a forward gate voltage pulse of 50 V is applied across the device, both MoS_2_ and SWCNTs exhibit electron‐type conduction and can release previously trapped holes through recombination with excess electrons. Therefore, after the forward gate voltage pulse ends, MoS_2_ will become nonconductive at reverse gate voltage and the device reverts to the nonconductive state. This is the principle of program/erase optical signals in this vdW heterostructure memory device.

Photoelectric memories that rely on a gate, source, and drain triterminal structure often suffer from high program voltages, high additional power consumption, and circuit complexity during integration. Tran et al.^[^ ^122^ ^]^ described multilevel nonvolatile optical memory devices of MoS_2_/h‐BN/Gr heterostructures with a two‐terminal floating‐gate field‐effect transistor (Figure 13e,f). The heterostructure possesses low programming voltage (−10 V), very low off‐current (≈10^−14^ A), and high current on/off ratio (≈10^6^). The device was produced by transfer and stacking of h‐BN flakes, single‐layer MoS_2_ in sequence, and Gr, followed by electron beam evaporation and electron beam lithography to form electrodes on MoS_2_. Using *V*_DS_ voltage and light illumination, the produced photoelectric memory device can control the tunneling of electrons to Gr to adjust the resistance of the MoS_2_ channel, enabling it to realize the programmable function of optical memory; optical storage operations originate from light‐generated charge carriers in MoS_2_.

**Figure 14:**
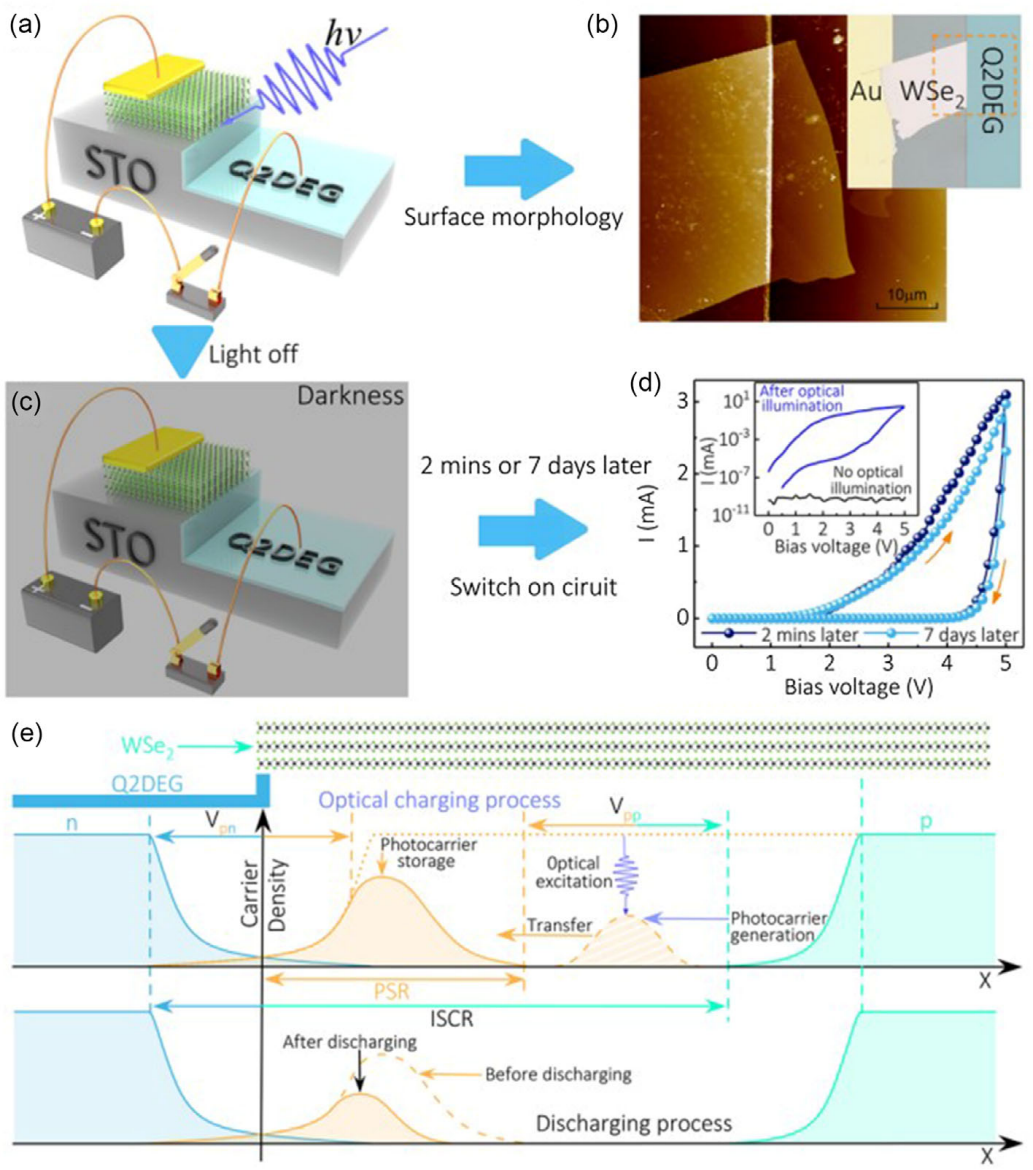
a) Schematic representation of the WSe_2_/Q2DEG heterostructure illuminated with 405 nm light at an intensity of 16 mW cm^−2^. The exposure time is 6 s, and the circuit is disconnected. b) AFM image depicting the surface morphology, with an inset showing an image of the device. c) Device subjected to darkness for either 2 min or 7 days at 30 K. d) *I*–*V* loops recorded in darkness 2 min and 7 days later, respectively, with an inset showing the *I*–*V* curves before and after optical illumination. e) Schematic illustrating the generation, storage, and discharge of photocarriers stored in the intrinsic space charge region (ISCR) during the optical charging and discharging process. Reproduced with permission.^[^ ^23^ ^]^ Copyright 2021, American Physical Society.

Jiang's group designed a vdW heterostructure composed of WSe_2_ and 2DEG.^[^ ^23^ ^]^ In this structure, they used an ingenious AIBA method^[^ ^94^ ^]^ to generate a 2DEG on the surface of STO. Experiments show that the heterostructure can store photogenerated carriers for up to 7 days after optical illumination; this is completely different from any photoelectric phenomenon observed before. In general photoconductivity, the regroup of electron–hole pairs follows their generation. At the same time, the device can maintain the same level of photocurrent after more than 1000 charge–discharge cycles. Based on the above phenomenon, the generation of photocarriers with an infinite lifetime in the heterostructures is a reasonable explanation. Photocarriers generated under the excitation of high‐energy photons are tightly locked in the heterostructures. This phenomenon discovered for the first time can be easily used in the design of memory devices, and the long‐term memory and multiple cycle characteristics exhibited are in perfect agreement with the current research direction of optical memory. In addition, another surprising phenomenon was also discovered^[^ ^31^ ^]^ in WSe_2_/2DEG heterostructures. Under the illumination condition, it shows the coexistence of giant positive and negative magnetoresistance, namely, GBU‐PhMR. By adjusting the thickness of WSe_2_, the device can obtain up to 4 900 000% positive magnetoresistance and −99.8% negative magnetoresistance. **Figure** **14** shows the structure and photoelectric properties of the device.

In some recent studies, perovskite materials and organic materials have also been put into the study of photoelectric memory,^[^ ^123^, ^124^, ^125^ ^]^ and memory devices based on these materials could be applied to artificial synapses for neuromorphic applications.^[^ ^126^, ^127^ ^]^ Although memory devices based on vdW heterostructures have achieved some results, they are not sufficient to replace current commercial storage devices, commercially available memory devices can be reused up to 100 000 times, and the advantages of vdW heterostructures make it have great development prospects in the manufacture of miniaturized, large‐capacity, foldable, highly transparent, and low‐power memory devices.

### 3.2 Solar Cells

Solar cells are devices that convert solar energy into electrical energy using the PV or photoelectrochemical effect. When the appropriate wavelength of sunlight irradiates the surface of the heterostructures, the heterostructures will absorb photons to generate electron–hole pairs, generate photocurrent, and realize photoelectric conversion.

The PV process occurs mainly in the junction, when irradiated by light of appropriate wavelength (energy equal to or greater than the bandgap of the semiconductor). The semiconductor absorbs the photon energy and electrons transition from the conduction band to the valence band, producing electron–hole pairs. In the presence of a self‐built electric field inside the junction, electron–hole pairs are separated and collected by the electrodes to produce electrical energy.

**Figure 15:**
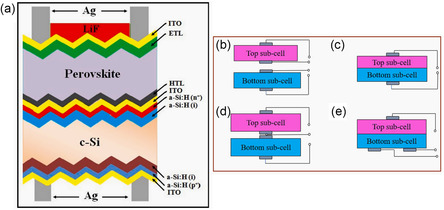
a) Graphical representation of a typical two‐terminal two‐junction bifacial perovskite/Si tandem solar cell device. Typical illustration of electrical connections for b) four‐terminal (4 T), c) two‐terminal (2 T), d) three‐terminal (3 T) middle, and e) three‐terminal (3 T) interdigitated back contact (IBC) tandem devices. Reproduced with permission.^[^ ^251^ ^]^ Copyright 2022, Elsevier Ltd.

**Figure 16:**
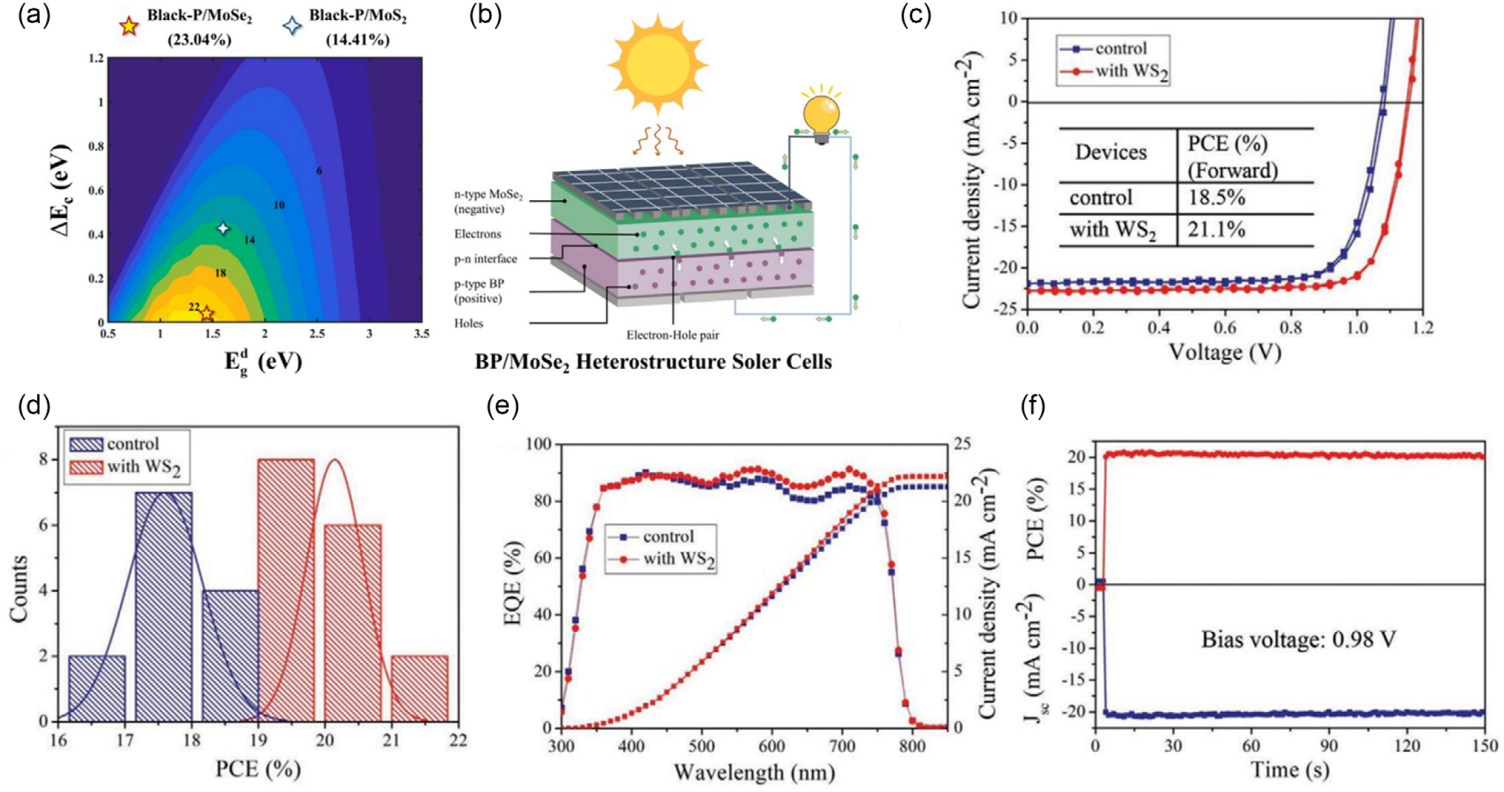
a) Computed PCE for the BP/MoSe_2_ (MoS_2_) heterostructure. b) Functioning mechanism of the BP/MoSe_2_ heterostructure in thin‐film solar cells. Reproduced with permission.^[^ ^133^ ^]^ Copyright 2022, Elsevier. c) *J–V* curves and e) EQE of the top‐performing devices with varying HTLs. d) PCE statistics of perovskite solar cells based (PSCs‐based) poly[bis(4‐phenyl)(2,4,6‐trimethylpheny)amine] (PTAA) and WS_2_‐modified PTAA. f) Consistent PCE and photocurrent density for the most efficient WS_2_‐based device. Reproduced with permission.^[^ ^134^ ^]^ Copyright 2020, WILEY‐VCH Verlag GmbH & Co. KGaA.

A complete solar cell should encompass the following components: light absorption layer, electron transport layer (ETL), electrolyte layer, charge isolation layer, anode, and cathode. Together, they achieve light absorption and conversion, as well as the collection and transmission of energy. Taking the example of a perovskite solar cell, **Figure** **15** shows a schematic diagram illustrating the classical structure of a perovskite solar cell. Also, **Figure** **16** shows the schematic diagram of 2DM heterostructures solar cell.

Anode (positive electrode), the anode of a perovskite solar cell, is typically composed of conductive glass, tin oxide, or other suitable conductive materials. This layer receives sunlight, allowing photons to enter the cell. Electrolyte, the electrolyte layer in a perovskite cell, is one of its distinguishing features. Typically, it is an organic substance, such as organic sodium salt. The electrolyte aids in the transport of electrons and holes, facilitating their separation. ETL: the ETL assists electrons in transferring from the perovskite layer to the cell's electrode. Commonly used materials include titanium oxide or other materials with excellent electron transport properties. Perovskite absorption layer: this is a critical part of the cell, typically composed of perovskite material, such as gas‐phase transition metal perovskite. The perovskite layer absorbs photons from sunlight and generates electron–hole pairs through the PV effect. Hole transport layer (HTL): the HTL facilitates the transfer of holes from the perovskite layer to the cell's electrode. This may involve polymer materials or other suitable substances. Cathode (negative electrode): the cathode of a perovskite cell is usually made of conductive materials, such as carbon or conductive polymers. It collects and exports the electron flow.^[^ ^128^, ^129^ ^]^ This configuration collectively achieves the efficient conversion of solar energy into electrical power. The schematic diagram illustrates the interplay of these components in a perovskite solar cell, highlighting their roles in the absorption, transport, and collection of solar energy.

Under operating conditions, the device's open‐circuit voltage (*V*_OC_) is defined as the maximum output voltage generated in the open‐circuit state, and the device's short‐circuit current (*I*_SC_) is defined as the maximum output current when the external circuit load is zero. Therefore, the product of *V*_OC_ and *I*_SC_ is the theoretical power of the PV device. In order to be able to quantitatively evaluate the performance of the device, the ratio of the maximum power output of the device to the theoretical power is defined as the fill factor (FF). The formula is expressed as

(1) $$\text{FF }=\;\frac{P_{\text{max}}}{V_{\text{OC}}I_{\text{SC}}}$$

where *P*_max_ is the maximum output power.

Power conversion efficiency (PCE), which is the ratio of the power output from the device to the input optical power received, can also be used as a performance criterion for the device. PCE can be calculated using the following formula.

(2) $$\text{PCE}=\frac{P_{\text{max}}}{P_{\text{in}}}-\frac{V_{\text{OC}}I_{\text{SC}}\text{FF}}{P_{\text{in}}}$$

where *P*_in_ is the input power.

In order to improve the competitiveness of solar cells with other clean energy sources and traditional energy sources, the efficiency and cost of solar cells should be the main points of consideration. If low cost and high efficiency can be concentrated in the same material, it will change the market pattern of solar cells. The addition of 2DMs is expected to achieve this goal. Currently, many 2DMs, such as Gr,^[^ ^130^ ^]^ TMDCs^[^ ^131^ ^]^ etc., have been used to design PV effect solar cells. Due to their unique electronic, photonic and structural properties, they are expected to be excellent transparent electrodes and ultrathin absorber layers for solar energy conversion. In this section, we will discuss the advantages and advances of 2DMs in PV devices and the application of 2D/2D and 2D/3D heterostructures.

When constructing solar cells using 2DMs, different properties of these materials can be applied to different layers and functional structures to optimize the performance of the solar cell. The exceptional conductivity of 2DMs proves advantageous for the conductive layer in solar cells. Serving as electrode materials, these 2D conductive materials offer high electron mobility, facilitating the efficient transport of electrons between electrodes. Moreover, the flexibility and robust mechanical strength inherent in 2DMs make them suitable for the support layer of solar cells. Leveraging the mechanical properties of 2DMs enhances the flexibility and stability of the cells. Additionally, the chemical stability exhibited by 2DMs becomes instrumental in the protective layer. Utilizing 2DMs with outstanding chemical stability safeguards other cell components from environmental chemical corrosion.

Gr's high carrier mobility, high transmittance, and excellent electrical transport properties make it promising for application as a transparent electrode and carrier transport layer in PV solar cells. However, Gr's zero‐bandgap properties limit its application as an absorber layer. In a recent study, researchers grew Gr in situ on both sides of a Cu–Ni alloy as the rear electrode of a chalcogenide solar cell, and the device with a Gr/Cu‐Ni/Gr electrode (CNG) exhibited stable operation for 5000 h with a stable PCE of 24.34%.^[^ ^132^ ^]^ Normal metal electrodes such as Ag and Al tend to react with halide anions in chalcogenide. Although Au is not susceptible to reaction, diffusion of Au atoms forms deep energy‐level defects. These affect the overall device lifetime. However, Gr in CNG electrodes can act as an excellent barrier, with the external Gr preventing oxygen and moisture penetration and corrosion and the internal Gr preventing direct contact between the electrode and the chalcogenide components. Compared to the Au electrode, the CNG electrode device still maintains 95% of the initial PCE after 5000 h, while the Au electrode device drops to 30% of the initial PCE after 1250 h. This study suggests that environmentally stable 2DMs (e.g., Gr) that are not prone to reactivity of the device components offer more options for electrodes in PV devices.

TMDCs are excellent candidates for fabricating high‐performance solar cells, due to their high optical absorption coefficient, suitable bandgap, and self‐passivating surface. However, the low carrier mobility of TMDCs compared to conventional materials such as Si limits their development. VdW heterostructures have emerged to provide a viable solution. Wang et al.^[^ ^133^ ^]^ proposed a 2D vdW heterostructure based on a BP single layer and MoSe_2_ single layer; they designed a novel high‐efficiency BP/MoSe_2_ and BP/MoS_2_ vdW heterostructure solar cell and systematically investigated the photoelectric properties of BP/MoSe_2_ and BP/MoS_2_ solar cells (Figure 16a,b). The bandgap of the BP/MoS_2_ heterostructure is 0.05 eV and that of the BP/MoSe_2_ heterostructure is 0.85 eV, which is closer to the ideal bandgap value (1.34 eV) showing more beneficial possible applications compared to the BP/MoS_2_ heterostructure. Moreover, the BP/MoSe_2_ heterostructure exhibits increased light absorption intensity and a broader absorption spectrum, with the dielectric function represented by the imaginary part *ε*_2_, indicating the energy needed for electric dipole formation. Compared with BP/MoS_2_, the ε_2_ energy level of BP/MoSe_2_ is lower and the peak is higher, indicating that the heterostructure of BP/MoSe_2_ is more prone to electronic leaps. Theoretical calculations show that the BP/MoSe_2_ heterostructure exhibits superior light absorption over the optical wavelength range of 0–6 eV, which covers the energy range of the solar spectrum. After calculation, it is found that the high energy conversion efficiency can reach 23.04%. This work shows that the choice of different 2DMs has a profound impact on solar cell applications. By combining different 2DMs, it is possible to obtain devices with ideal properties.

**Figure 17:**
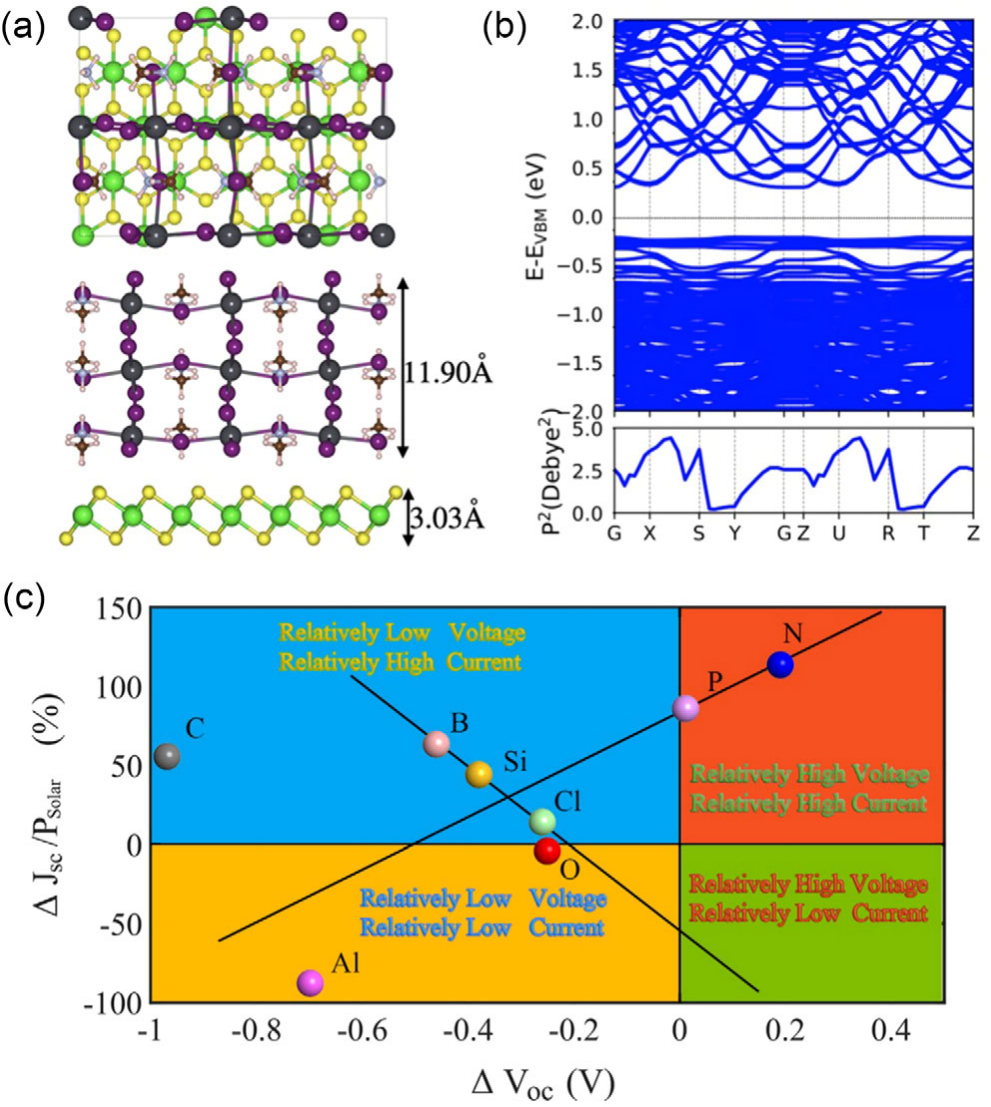
a) The fully optimized structure with overhead and lateral views illustrates the thickness of the CH_3_NH_3_PbI_3_/HfS_2_ vdW heterostructure. b) The transition probability (p2) and electronic band structure of the CH_3_NH_3_PbI_3_/HfS_2_ vdW heterostructure. Reproduced under terms of the CC‐BY 4.0 license.^[^ ^135^ ^]^ Copyright 2022, The Authors. Published by American Chemical Society. c) Relative *V*_oc_ and *J*_sc_ (compared to the *V*_oc_ and *J*_sc_ of pure GaS/SnS_2_ heterostructures) of GaS/SnS_2_ heterostructures doped with various elements. Reproduced with permission.^[^ ^138^ ^]^ Copyright 2019, WILEY‐VCH Verlag GmbH & Co. KGaA.

Not only 2D/2D heterostructures, but also 2D/3D heterostructures are used in PV devices. The perovskite structure is currently a popular choice for PV applications, and a variety of perovskite‐based MVDWHs are applied in solar cells.^[^ ^129^ ^]^ In solar cell applications, the quality of the material is closely related to the performance of the device. 2DMs with defect‐free surfaces offer opportunities. Cao et al.^[^ ^134^ ^]^ used layered WS_2_ with defect‐free surfaces as templates for vdW epitaxy growth of hybrid perovskite thin films, the hybrid perovskite films grow well along the (001) direction on the WS_2_ surface, forming a perfect perovskite/WS_2_ interface (Figure 16c–f). Meanwhile, the perovskite/WS_2_ interface can enhance the charge extraction at the interface and inhibit the interfacial charge complexation in perovskite solar cells. The final chalcogenide solar cell with up to 21.1% PCE was obtained. The perovskite/WS_2_ device maintained 90% of the initial PCE after 1 month compared to the device without WS_2_, while the comparison device had only 70%. The introduction of WS_2_ improved the quality of the perovskite film and reduced the interfacial defects, leading to an increase in device stability. This suggests that the use of 2DMs as an interfacial layer for chalcogenide solar cells to improve device performance is a viable strategy. Finally, perovskite heterostructure solar cells with a PCE as high as 21.1% were fabricated. Deobrat Singh et al.^[^ ^135^ ^]^ proposed the combination of perovskites and TMDCs, using CH_3_NH_3_PbI_3_/HfS_2_ vdW heterostructures (**Figure** **17**a,b) as an example of PV applications. Using first‐principles calculations of the electronic and optical properties of the structure, high visible light absorbance is obtained at the CH_3_NH_3_PbI_3_/HfS_2_ heterostructure interface due to the establishment of an internal electric field separating the electron–hole pairs. The structure is considered to achieve up to 28.45% PCE. 2D/3D heterostructures in PV cells can increase photoelectron yield, optimize charge separation and transport, enhance mechanical strength, and expand spectral response range.

In addition, a number of designs have been proposed to improve the performance of solar cells, Hazem Abdelsalam et al.^[^ ^136^ ^]^ considered the use of arsenene, silicene, and Gr 2DQDs for the design of high‐efficiency heterostructures solar cells and calculated by density functional theory that the PCE of silicene/Gr solar cells can reach 23.34%.

M. Furchi et al.^[^ ^137^ ^]^ explained the electro generation behavior through a systematic experimental study of MoS_2_/WSe_2_ vdW heterostructures and proposed to improve the PCE of solar cells by increasing the electrical conductivity. The conductivity of the device can be effectively improved by the heteroatom doping strategy. The results of Wu et al.^[^ ^138^ ^]^ showed that using heteroatom doping can significantly improve PCE. Their N‐atom doping strategy effectively enhances the PCE of 2D GaS/SnS_2_ and GaSe_2_/SnS_2_ heterostructures by 16%, an improvement of 162% over the undoped heterostructures (Figure 17c). Higher PCE can also be achieved by passivation of the interface of the heterostructures by intercalation layers. Park et al.^[^ ^139^ ^]^ used ZnS nanolayers as intercalation between heterostructures to passivate the interface of the heterostructures. They inserted ZnS nanolayers in MoS_2_/Cu(In_1−*x*_, Ga_*x*_)Se_2_ (CIGSe) heterostructures to enhance the photoelectric behavior of the device; compared with the heterostructures without intercalated ZnS, the PCE is more than doubled. In summary, the performance of heterostructure solar cells can be effectively enhanced by optimizing the strategies of material selection, heteroatom doping, interface passivation, and modulation of heterostructure interface.

At present, the PCE of commercial Si solar cells is about 25%,^[^ ^140^ ^]^ In comparison, the efficiency of solar cells composed entirely of 2DMs is not satisfactory. Although theoretical calculations show that 2DMs heterostructure cells have vast prospects, it is still necessary to find a suitable strategy to realize them. The application of 2DMs as the absorbing layer in solar cells faces certain limitations. Most 2DMs have relatively limited light absorption within the visible spectrum. This may result in insufficient absorption of photons in the solar spectrum, thereby reducing the efficiency of PV conversion. Due to the inherent properties of 2DMs, their thickness is typically very thin, only a few atomic or molecular layers thick. This thinness may restrict the number of absorbed photons, limiting the generation of electron–hole pairs. According to the prediction, more than 4000 types of 2DMs may exist, and even a simple permutation of the vdW heterostructure still has infinite possibilities. Combining 2D and 3D materials is a useful idea at present, and various methods including chemical doping, antireflective coatings, and interfacial layer insertion have been used to improve the performance of devices. The introduction of 2DMs has facilitated device design and significant performance enhancement can be obtained by modifying or replacing some components by 2DMs, bandgap engineering increases light absorption, and charge separation and transfer at the heterostructure interface increase efficiency. Interlayer excitons, formed between adjacent layers, exhibit a prolonged lifetime compared to individual layers. The binding energy of interlayer excitons is influenced by the thickness of the inserted insulating interlayer. These characteristics offer opportunities for the development of novel solar cells with enhanced light absorption and conversion efficiencies. VdW solar cells are anticipated to find applications in cost‐effective and atomically thin solar cell technologies. With the emergence of materials such as perovskites, TMDCs, MXenes^[^ ^141^ ^]^ etc., vdW heterostructures solar cells still have great potential to be exploited.

### 3.3 Electroluminescent Device

Light‐emitting devices usually mean the functional devices that emit electromagnetic waves in different wavelength bands such as visible light, infrared, and ultraviolet. The earliest ones used are light‐emitting devices based on thermal radiation, which are inefficient and have poor cyclability. After the discovery of self‐excited radiation, it quickly replaced thermal radiation devices. At present, electroluminescent‐junction semiconductor light‐emitting devices have been reported by exploring the electroluminescence (EL) phenomenon^[^ ^142^, ^143^ ^]^ in vdW heterostructures.

**Figure 18:**
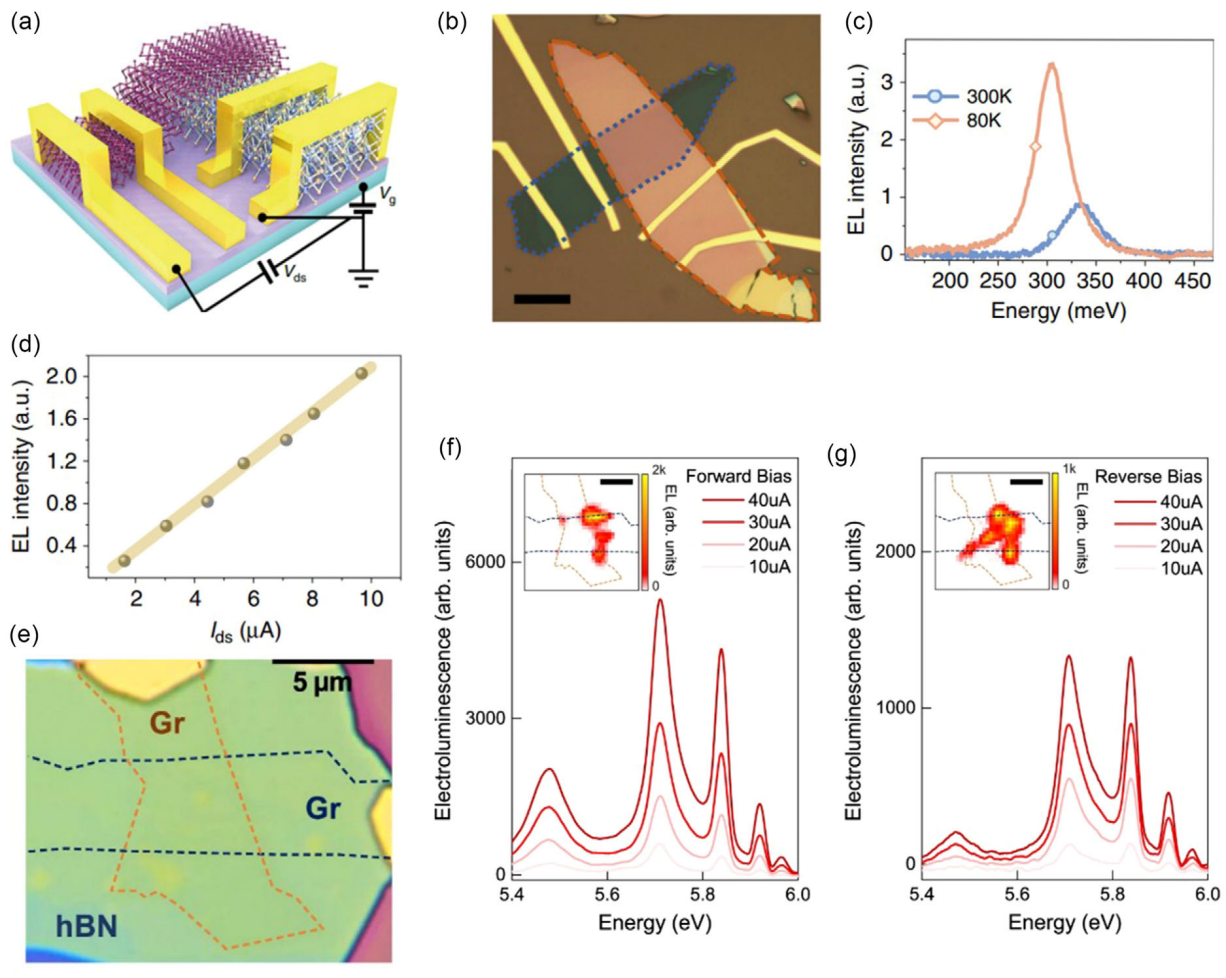
a) Diagrammatic representation and b) optical pictures of the BP/MoS_2_ heterostructure diode. The scale bar denotes 10 μm. BP and MoS_2_ thin films are demarcated by orange and blue dotted lines, respectively. c) EL at 80 K with an *I*_ds_ of 8.05 μA (depicted by the orange line) and at 300 K with an *I*_ds_ of 8.50 μA (illustrated by the blue line). d) EL intensity in relation to source–drain current *I*_ds_ when *V*_ds_ > 0. The yellow solid line acts as a guide line. Reproduced under terms of the CC‐BY 4.0 license.^[^ ^144^ ^]^. Copyright 2020, The Authors. Published by Springer Nature Limited. e) Optical microscope image of the selected device for EL assessment (scale bar: 5 μm). The upper and lower Gr electrodes are indicated by orange and blue dashed lines, respectively. DUV EL spectra are provided for current values ranging from 10 to 40 μA under both forward and reverse biases in panels f,g), respectively. Reproduced under terms of the CC‐BY 4.0 license.^[^ ^97^ ^]^ Copyright 2021, The Authors. Published by Springer Nature Limited.

Zong et al.^[^ ^144^ ^]^ used thin‐film BP and TMDCs to construct a vdW heterostructure with BP as the midinfrared (MIR) light‐emitting layer. The BP/MoS_2_ heterostructures form the ii‐type band alignment, in addition, due to the existence of defects, thin films BP and MoS_2_ exhibit *p*‐type and n‐type semiconductor properties, respectively, so a p–n heterostructure is naturally formed at the BP–MoS_2_ interface. By adjusting the source–drain voltage, electrons can be transferred from the MoS_2_ conduction band to the BP conduction band, resulting in MIR EL in BP. **Figure** **18**a,b shows the schematic and optical photos of the BP/MoS_2_ heterostructures diode, respectively. Figure 18c,d shows the EL characteristics of this device.

Song et al.^[^ ^97^ ^]^ reported the phenomenon of deep‐ultraviolet (DUV) EL in Gr/h‐BN/Gr heterostructures. In this study, injecting charge carriers of Gr electrodes into the band edges of h‐BN can produce remarkable EL phenomena at DUV frequencies. In the vdW stack of two h‐BN crystals, the luminescence spectrum can be significantly changed by the superposition angle. Figure 18e–g indicates the DUV emission region of the device.

Sun et al.^[^ ^145^ ^]^ successfully realized a femtosecond solid‐state laser by Gr/MoS_2_ heterostructures; through the study of Gr/MoS_2_ interlayer coupling, a variety of controllable superimposed vdW heterostructures can be fabricated. This study demonstrates theoretically and experimentally the effect of MoS_2_ thickness on the nonlinear optical response of Gr/MoS_2_, showing the promising application of vdW heterostructures in laser devices.

In addition, some studies investigated the mechanism and application of EL. Hou et al.^[^ ^146^ ^]^ demonstrated that Gr/h‐BN/WS_2_/h‐BN/Gr devices fabricated using the CVD growth method could continuously operate for more than 2 h under constant atmospheric conditions, producing strong red EL. The operational limit and failure mechanism of 2D vdW heterostructures devices with long‐life sustained EL properties are also investigated. Li et al.^[^ ^147^ ^]^ studied the MoS_2_/InSe vdW heterostructures and their application in 2D photoelectric devices, which exhibited strong luminescence at a wavelength of 1020 nm with a turn‐on voltage of 0.8 V. Hu et al.^[^ ^148^ ^]^ fabricated flexible transparent NIR photodetector arrays with Ti_3_C_2_T_*x*_ MXene‐RAN vdW heterostructures. They created a 1024‐pixel image sensor through the integration of flexible photodetectors into a 32 × 32 array, showcasing distinct and intricate "deer" patterns. VdW heterostructures electroluminescent devices can operate at low voltage and low temperature and have good transparency, which can be well used in future smart devices.

## 4 VdW heterostructures for Memory Devices

With the rapid development of neural network applications, large amounts of data need to be processed, requiring next‐generation nonvolatile memory (NVM) technologies with high speed and large storage.^[^ ^149^ ^]^ Memory performance becomes a key factor to limit the efficiency of the overall system. VdW heterostructures consist of layered materials^[^ ^150^ ^]^ that have been demonstrated to enhance memory device performance in terms of energy storage, both by theoretical calculations and by experimental studies.^[^ ^151^, ^152^ ^]^ The versatility of the vdW stacking technique allows engineering structures that are not constrained by lattice parameters.^[^ ^153^ ^]^ By selecting suitable vdW heterostructure materials, we can construct FTJs, ferroelectric field‐effect tubes, and memory resistors to realize functional devices with memory properties.

### 4.1 Ferroelectric Tunnel Junction

**Figure 19:**
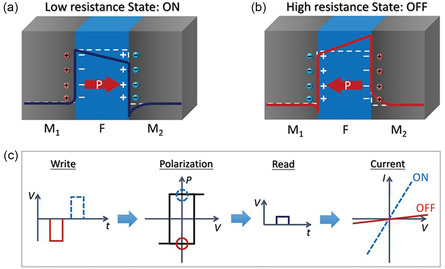
The TER effect in an MFM‐type FTJ device is depicted through barrier profiles, showcasing the a) ON and b) OFF states aligned with the direction of polarization indicated by red arrows. (a, b) Adapted with permission.^[^ ^163^ ^]^ Copyright 2005, American Physical Society. c) The operational procedures of an FTJ device involved using a write pulse to alter the polarization, followed by reading the junction current with a minor pulse to ensure the polarization remains unchanged. Reproduced with permission^[^ ^160^ ^]^ Copyright 2019, WILEY‐VCH Verlag GmbH & Co. KGaA.

FTJs, consisting of two metallic electrodes and a thin ferroelectric barrier, have attracted significant interest in technical applications as NVM devices.^[^ ^154^, ^155^, ^156^, ^157^, ^158^, ^159^ ^]^ In conventional 3D FTJs, there is a limit to the spontaneous polarization observed in the critical thickness of 3D ferroelectric materials due to the buildup of charges on the surface, which contradicts the requirement for device miniaturization.^[^ ^159^ ^]^ Therefore, how to reduce the critical thickness of ferroelectric films is a crucial problem for future development of high‐performance FTJs.^[^ ^155^ ^]^ The growing interest in 2D FTJs is driven by their atomic thinness and their potential impact on downsizing FTJ devices.^[^ ^155^ ^]^ When exploring FTJs, the electric potential distribution can be altered through polarization reversal, inducing a nonvolatile switch in the junction conductance between high (ON)‐ and low (OFF)‐resistance states, commonly referred to as tunneling resistance (TER), as depicted in **Figure** **19**.^[^ ^160^ ^]^

TER ratio $\left(=\frac{J_{\text{ON}}-J_{\text{OFF}}}{J_{\text{OFF}}}\times 100\%\right)$ is a very important parameter to characterize data storage performance.^[^ ^157^, ^158^ ^]^ FTJs seek higher TER values and better switching performance,^[^ ^161^ ^]^ TER ratios have improved by more than 6 orders of magnitude over the past decade.^[^ ^160^, ^162^ ^]^

**Figure 20:**
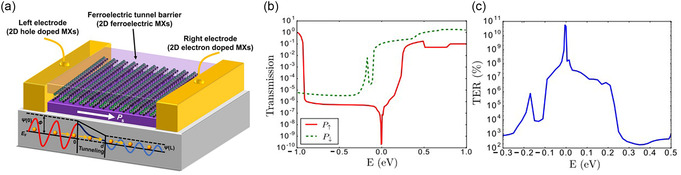
a) Schematic illustration of a 2D FTJ device constructed on a homostructure. The tunneling barrier consists of a pure 2D monochalcogenide (MX) semiconductor exhibiting in‐plane ferroelectricity. Both the left and right electrodes are formed through *p*‐ and n‐type doping of the identical MX material. The inset highlights the wave–particle duality inherent in quantum tunneling, where electrons traverse the barrier as an evanescent state, exhibiting exponential decay in amplitude as they penetrate through the barrier. Reproduced with permission.^[^ ^164^ ^]^ Copyright 2021, American Chemical Society. b) The transmission function for both directions of polarization, with the Fermi level fixed to 0 eV; c) The TER ratio in relation to electron energy. Reproduced with permission.^[^ ^155^ ^]^ Copyright 2020, American Physical Society.

The 2D vdW ferroelectric layer can shrink the FTJs while maintaining a high TER value. Kang et al.^[^ ^159^ ^]^ designed a 2D vdW FTJ using a Gr/BiP vdW vertical heterostructure as the lead and a monolayer BiP as the center of ferroelectric barrier. The left and right leads’ symmetry was broken by adding B/N atoms to the left and right Gr layers. This resulted in asymmetry shielding lengths for the two leads, which produced various potential barriers. As soon as the giant resistance effect in FTJs was conceptualized,^[^ ^163^ ^]^ asymmetric leads with various shield lengths were needed for the mechanism. The ferroelectric tunneling barrier works because the average tunneling average barrier height changes when the polarization is reversed. The variation of the tunnel barrier leads to different transmissions. A huge TER ratio of about 623%^[^ ^106^ ^]^ is obtained in this FTJ. The significant TER ratio is caused by the asymmetric shielding effect of the B/N‐doped vertical vdW, according to the analysis of the photoelectric properties.^[^ ^159^ ^]^ Shen et al. proposed doping engineering in 2D in‐plane ferroelectric semiconductors (FeS‐FET) as a successful method for designing 2D FTJs composed of homostructured *p*‐type semiconductors/n‐type semiconductors, as shown in **Figure** **20**a.^[^ ^164^ ^]^ The monolayer ferroelectric potential barrier retains an in‐plane polarization, enabling the creation of a 2D FTJ with ultrathin vertical thickness. Utilizing density general function theory and nonequilibrium Green's function form, the TER effect was investigated in a specially designed 2D‐FTJ In:SnSe/SnSe/Sb:SnSe heterostructure, revealing a substantial TER effect of 1460%. This effect stems from the tunable tunneling barrier width, a unique feature not commonly found in all‐oxide FTJs, along with the tunneling barrier height, which governs the amplified TER effect.^[^ ^164^ ^]^

The polar properties of 2D ferroelectrics can induce metal–insulator transitions by building heterostructures rather than carefully selecting asymmetric lead materials. One possible approach is to use ferroelectrodization to alter charge transfer and the energy band bending close to the interface. For instance, Kang et al. developed 2D vdW FTJs, at the interface of such heterostructures, where charge transfer may or may not occur, which would result in the 2D polar materials to either conducting or remain insulating, leading to two different conducting states (“on” and “off”). A huge TER ratio of about 1 × 10^8^% was obtained using a Gr/*α*‐In_2_Se_3_ vertical heterostructure as two leads and an *α*‐In_2_Se_3_ monolayer as a transport channel. The energy band structure of 2D Gr and 2D In_2_Se_3_ hardly changes in the event of upward polarization, which implies an insulating state for 2D In_2_Se_3_. However, the bottom of conduction band of 2D In_2_Se_3_ crosses the Fermi energy level owing to the charge transfer between 2D Gr and 2D In_2_Se_3_, suggesting a metallic state of 2D In_2_Se_3_. Also, considering the ferroelectric and antiferroelectric states in the multilayer and bilayer *α*‐In_2_Se_3_, it is conceivable that there exists an anti‐FTJ with two states with significant TERs. A promising pathway for the application of out‐of‐plane polarized 2D ferroelectric materials to ferroelectric memory devices is provided.^[^ ^155^ ^]^

### 4.2 Ferroelectric Field‐Effect Transistor

**Figure 21:**
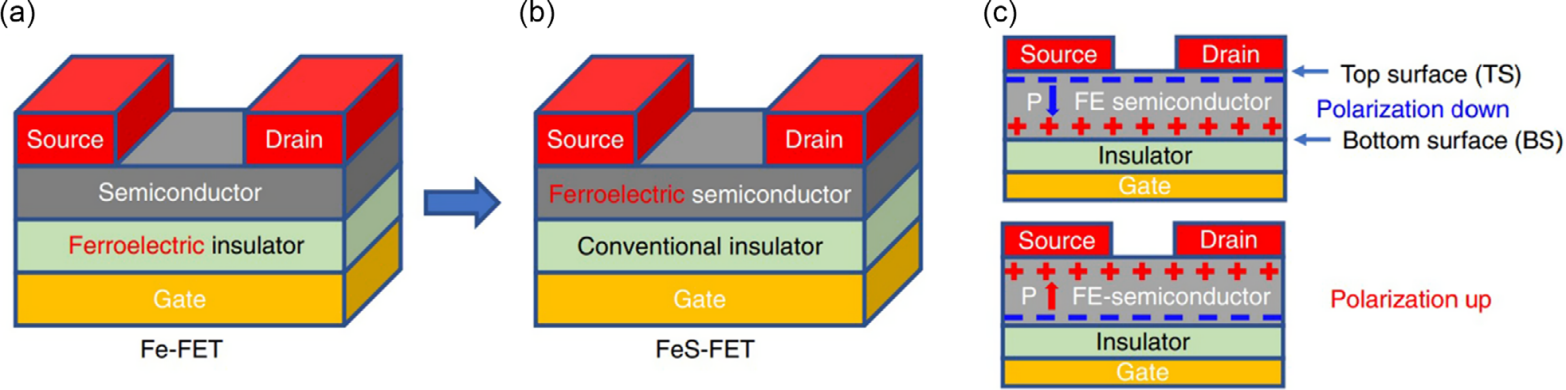
a) Illustration of FeFET. b) Representation of FeS‐FET. In the FeS‐FET, the conventional semiconductor channel is replaced with a FeS‐FET, while the gate insulator remains a standard dielectric. c) Distribution of polarization‐bound charges in a FeS‐FET, showcasing polarization down (resulting from negative gate bias) and polarization up (resulting from positive gate bias) states. Reproduced with permission.^[^ ^165^ ^]^ Copyright 2019, Springer Nature Limited.

2D FeFETs, a device formed by replacing the oxide insulating layer in a metal‐oxide‐semiconductor field‐effect transistor (MOSFET) structure with a ferroelectric material,^[^ ^165^ ^]^ are shown in **Figure** **21**a. In this device, we used channel conductance to detect the polarization state of the gate, enabling lossless data read operations in FeFETs. In this structure, the polarization of the ferroelectric material is used to influence the resistive state of the semiconductor material. The most significant properties of ferroelectric materials are spontaneous polarization due to the asymmetry of their own structure and the fact that this polarization can be altered by an external electric field and retained, called residual polarization. Different polarization states of the material correspond to different resistive states, a feature that makes it suitable for storage applications. In a typical FET, the magnitude of *I*_ds_ depends on *V*_g_, and in this respect, the mechanism of FeFETs operates under the same mechanism as conventional MOSFETs. However, the residual polarization of the ferroelectric material leads to high and low resistance states, and this property affects the channel conductance, which can be measured to detect the polarization state of the ferroelectric material.^[^ ^165^ ^]^ However, severe carrier charge trapping and depolarization effects greatly limit the performance of the memory, thus hindering the commercialization of FeFETs for NVM.^[^ ^166^, ^167^ ^]^ The recent emergence of 2D vdW ferroelectrics^[^ ^168^, ^169^ ^]^ combined with various 2DMs ranging from metals and semiconductors^[^ ^165^ ^]^ to insulators^[^ ^170^ ^]^ offers the possibility to realize persistent storage FeFETs memories via vdW heterostructures. VdW engineering is a possible direction for enhancing the stability and performance of next‐generation ferroelectric memories.^[^ ^167^ ^]^

The absence of surface dangling bonds is one of the main advantages of 2DMs, ideal for flexible design of memory devices seeking superior memory performance.^[^ ^171^ ^]^ Possibility of square ferromagnetic hysteresis loops presents in single‐crystal vdW ferroelectric layers, which help to eliminate retention losses due to depolarization fields. Wang et al. demonstrated a time‐proof FeFET memory cell with a metal–ferroelectric–metal–insulator–semiconductor structure fabricated from all‐2D materials. It shows 17 mV dec^−1^ operation with a memory window greater than 3.8 V, a maximum drain current of 671 μA μm^−1^, and a program/erase ratio up to 10^7^, more than 10^4^ cycles of endurance, 10 years of memory storage, and program/erase speed of less than 5 μs. A pulse of 100 ns is sufficient to reverse the polarization, while a 100 ns pulse train demonstrates 4‐bit cell^−1^ operation of a vdW ferroelectric transistor.^[^ ^167^ ^]^

**Figure 22:**
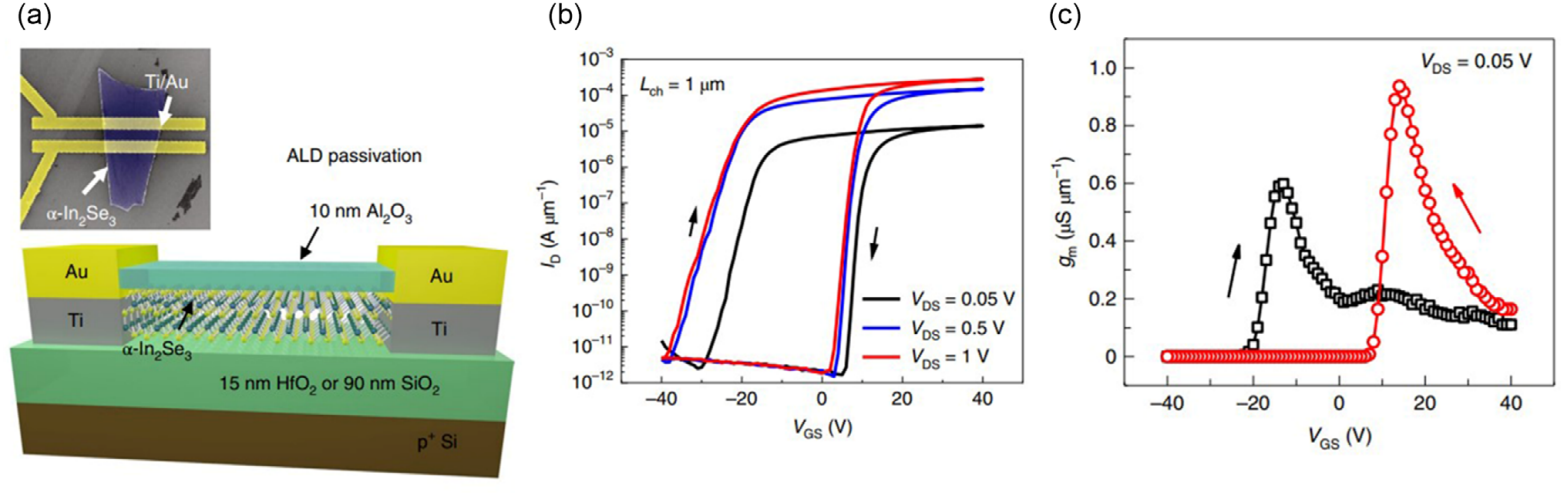
a) Schematic of the *α*‐In_2_Se_3_ FeS‐FET subjected to atomic layer deposition (ALD) passivation, accompanied by a false‐color top‐view SEM representation of a fabricated *α*‐In_2_Se_3_ FeS‐FET. b) Room‐temperature characteristics, including *I*_D_–*V*_GS_ and c) *g*_m_–*V*_GS_ for a representative *α*‐In_2_Se_3_ FeS‐FET with a 90 nm SiO_2_ gate dielectric and ALD passivation. The device, featuring a 1 μm channel length and a 52.2 nm channel thickness, exhibits a substantial memory window, a peak drain current of 671 μA μm^−1^, an on/off ratio surpassing 10^8^, and notable electron mobility (μFE) reaching 312 cm^2^ V^−1^ s^−1^ in forward sweep and 488 cm^2^ V^−1^ s^−1^ in reverse sweep. Reproduced with permission.^[^ ^165^ ^]^ Copyright 2019, Springer Nature Limited.

Si et al. reported on FeFETs made of FeS‐FETs, as Figure 21b shows. This structure is a conventional MOSFET in which the semiconductor layer is replaced by a FeS‐FET layer, and its operation mechanism is similar to that of FeFETs, which also utilize the polarization‐altering property of the ferroelectric material. Unlike FeFETs, the polarization of the FeS‐FET itself changes its own resistive state. FeS‐FETs detect the polarization state of the FeS‐FET directly instead of indirectly obtaining the polarization state of the ferroelectric material using a channel, which reduces the error in the information transmission process. Meanwhile, mobile charges in a semiconductor have the ability to shield the depolarization field of the entire semiconductor. This is critical to eliminate charge trapping and leakage currents through ferroelectric insulators in traditional FETs. In summary, the FeS‐FET achieves resistive switching (RS) through self‐polarization, whereas the FeFET utilizes a ferroelectric layer for RS. In this structure, they used *α*‐In_2_Se_3_ as the channel material of the device, see **Figure** **22**. *α*‐In_2_Se_3_ was chosen because of its room‐temperature ferroelectricity, suitable bandgap, potential for large‐area growth, and the ability to maintain ferroelectricity down to several atomic layers.^[^ ^165^ ^]^ They protect and optimize the performance of the device by depositing an atomic layer of Al_2_O_3_ to passivate the material. The fabricated FeS‐FETs feature high on/off ratios of over 10^8^, large storage windows, low supply voltages, and maximum currents of 862 μA μm^−1^. FeS‐FETs may be a better choice than existing Fe‐FETs in NVM applications.^[^ ^165^ ^]^

FeFETs show promise for high‐performance devices, as hafnium zirconia (HZO) can be obtained through complementary MOS‐compatible processes.^[^ ^172^ ^]^ Zhang et al. fabricated HZO (6 nm)/Al_2_O_3_ (2 nm) 2D vdW structured memory devices using the representative material MoS_2_ with a switching ratio of about 10^7^ and high endurance and retention.^[^ ^173^ ^]^ Various studies on the structure of FeFETs and the memory window altered by HZO‐FeFETs are actively being conducted in the memory field.^[^ ^174^, ^175^ ^]^

### 4.3 Memristors

Resistive devices with inherent memory effects (memory + resistor) are called memristors.^[^ ^176^ ^]^ Memristors have easy‐to‐fabricate, simple metal–insulator–metal (MIM) structures^[^ ^177^ ^]^ that use electrical signals to induce reversible conversions between two states of low resistance (LRS, ON) and high resistance (HRS, OFF) in the intermediate.^[^ ^178^ ^]^ A memristor for memory applications can be scaled down to less than nanometer size^[^ ^179^ ^]^ and maintain a long storage state while providing ideal characteristics like subnanosecond switching speed,^[^ ^180^, ^181^ ^]^ long write erase persistence,^[^ ^182^ ^]^ and low programming energy (e.g., nanoampere^[^ ^183^ ^]^).^[^ ^176^ ^]^ Amnesic resistors have been proposed as candidates for next‐generation NVMs.^[^ ^176^, ^184^, ^185^, ^186^ ^]^ Notably, while many of these favorable characteristics have been tautologically implemented, realizing them in a single‐material system at the same time remains a challenge.^[^ ^176^ ^]^ By stacking 2D layered structures together,^[^ ^187^ ^]^ the excellent characteristics of each 2D compositions can concentrate in vdW heterostructures. By this way, heterostructures are used to challenge existing difficulties in electronic device development.^[^ ^185^ ^]^

**Figure 23:**
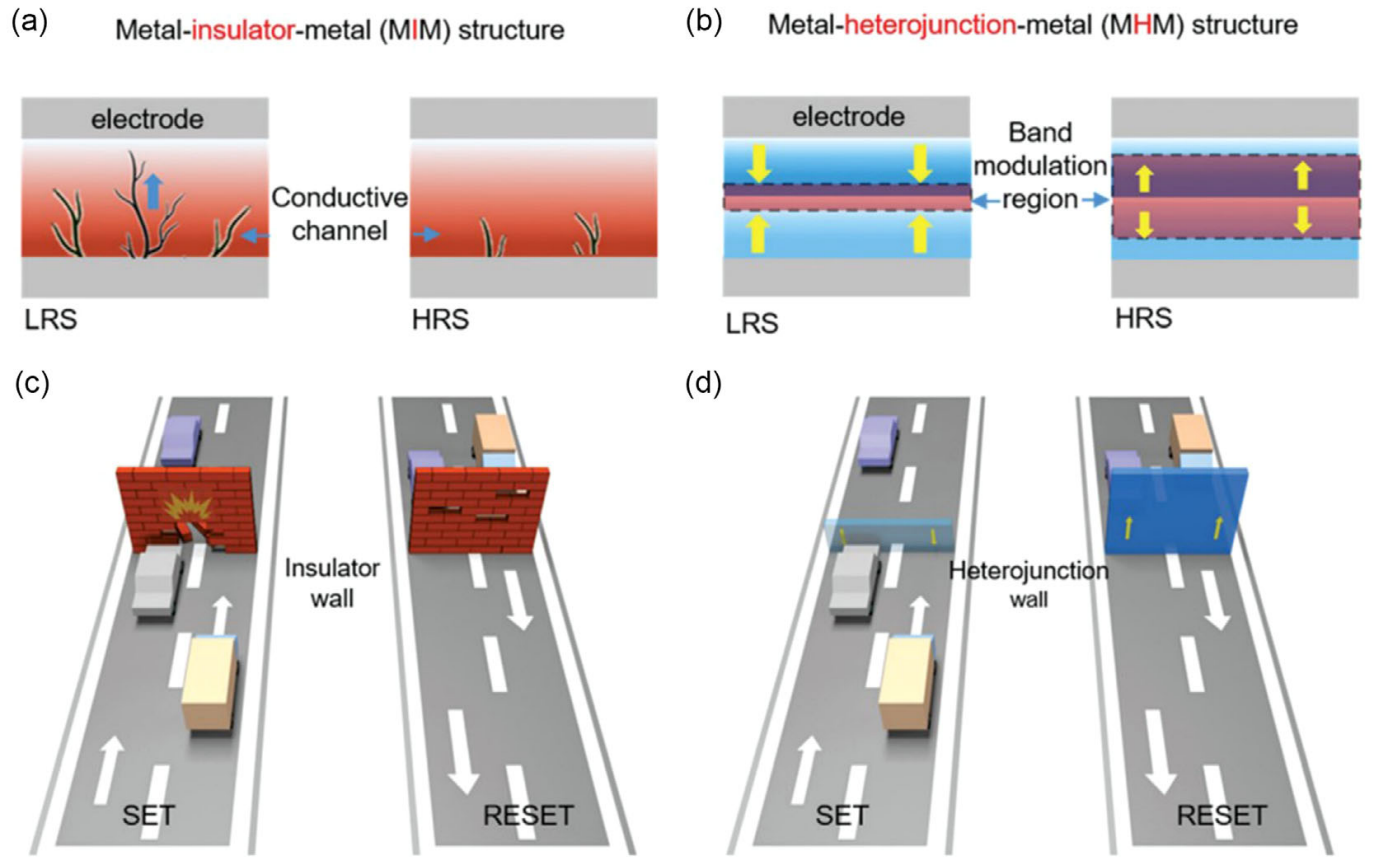
The metaphorical analogy of the memristive mechanism in MIM and MHM memristor structures. a,c) The conventional MIM structure, where the insulating material serves as the functional layer can be likened to a “brick wall” on a highway. The conductive channel formed under applied voltage inflicts irreversible “damage” on the device, similar to a strike on the “brick wall,” leading to rapid failure at the nanoscale; b,d) in contrast, the MHM structure with a heterojunction resembles a “lift gate” on the highway. Resistance is modulated by the band structure of the heterogeneous junction, preventing direct “damage” to the memristor layer and resulting in more stable performance. Reproduced with permission.^[^ ^188^ ^]^ Copyright 2021, Royal Society of Chemistry.

Zhang et al. proposed a metal/heterostructure/metal (MHM) memristor structure based on WS_2_/MoS_2_ heterostructure and showed holistic memristor performance of this device prepared utilizing a low‐cost transfer‐free 2DMs synthesis technology. The traditional MIM structure obtained memory properties through the formation and destruction of metallic filament. In contrast, the mechanism of the MHM structure is the modulation of the energy band. So, the 2D memristors based on MHM structure can remove the severe structural damage to the 2DMs due to filament formation in the setup process and it makes the memristors more stable (**Figure** **23**). The devices based on WS_2_/MoS_2_ heterostructure can realize a switching ratio of 10^4^ and can retain a set cycling process of more than 120 times under a steady storage window. The MHM‐based 2D memristors can simultaneously improve the stability and realize high‐density integration of the 2D material‐based memristors, and it's expected to lay the route for future low‐power memory implementation.^[^ ^188^ ^]^

The development of memristors is hampered by uncontrolled defects at the interface after filament formation and structural damage of materials, which reduce memory performance (stability and variability) and even device failure, especially for devices with vertical configuration.^[^ ^189^, ^190^ ^]^ Mao et al. showed an atraumatic memristor by vdW metal transfer method through lamination on laminar h‐BN. With this technique, the 2D h‐BN laminate was well protected with minimal damage and residuals. The memristor based on Au/h‐BN/Au structure showed powerful nonvolatile nonpolar RS with a high switching ratio, long time of duration, fast switching speed, and fast operation cycle with volatile threshold RS. Cross‐sectional scanning transmission electron microscopy (STEM) helped to verify the importance and feasibility of this approach in reducing damage and the decisive effect of the atomically sharp Au/h‐BN interface in optimizing RS behavior.^[^ ^184^ ^]^

**Figure 24:**
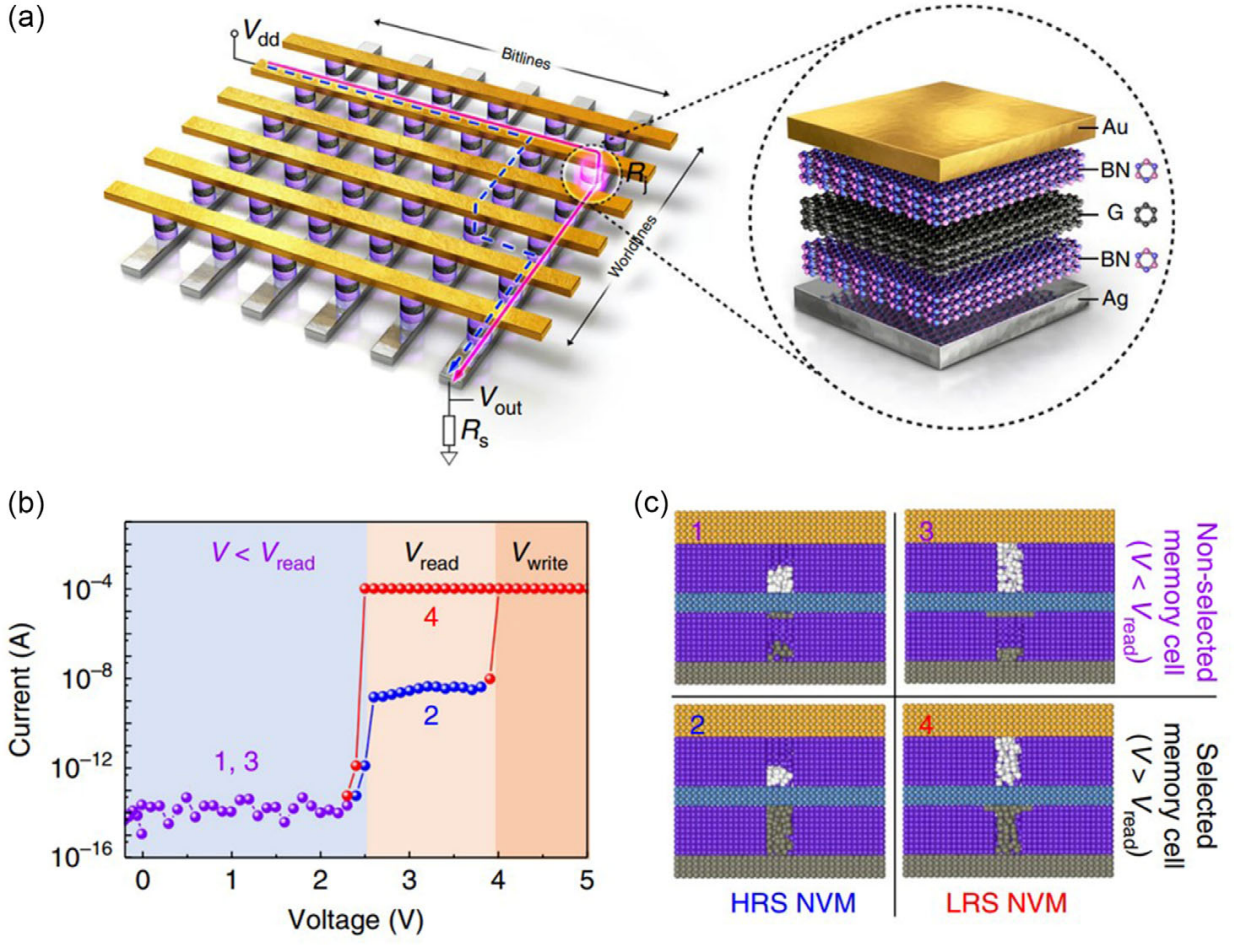
Operational mechanism of a crossbar memory array using a self‐selective vdW heterostructure. a) Schematic representation of the vdW heterostructure within the crossbar memory array structure, deviating from the conventional one‐selector one‐resistor and complementary RS (refer to main text). b) *I–V* characteristics of a single memory cell with four distinct states. The four current and voltage ranges signify different states of the memory cell, demonstrating bipolar behavior. The self‐selective cell achieves a selectivity of 10^10^, accompanied by a substantial memory window (10^3^). c) Schematic depiction of the h‐BN/Gr/h‐BN layers for the four states in ‘b’. Ranges “1” and “3” denote the high‐resistance state and low‐resistance state of unselected cells, respectively. Ranges “2” and “4” represent the high‐resistance state and low‐resistance state of a selected memory cell, respectively. Conductive silver filaments form at a voltage of 2.6 V, enabling the reading of the high‐resistance state (range “2”) and low‐resistance state (range “4”) within a voltage window from 2.6–4.0 V. The gray, purple, blue, and yellow spheres symbolize silver, h‐BN, Gr, and gold layer, respectively. The white spheres in the top h‐BN layer represent boron vacancies in h‐BN. Reproduced under terms of the CC‐BY 4.0 license.^[^ ^192^ ^]^ Copyright 2019, The Authors. Published by Springer Nature Limited.

Double‐ended vertically stacked memristors have shown great potential in meeting the great need for building high‐density, large‐scale neuromorphic chips. However, RRAMs in crossbar configurations often suffer from inaccurate reads and programming due to currents through unselected cells (“hidden path currents”).^[^ ^191^ ^]^ Self‐rectifying memristors effectively solve the problem of steganography arrays. Sun et al. fabricated a self‐selective memory cell made of the h‐BN/Gr/h‐BN vdW device.^[^ ^192^ ^]^ The h‐BN/Gr/h‐BN heterostructure was introduced at each crossover point under the crossbar array structure of Ag and Au electrodes (**Figure** **24**a). The Au/h‐BN/Gr structure formed nonvolatile boron vacancy filaments and the Gr/h‐BN/Ag formed volatile silver filaments. The nonvolatile and volatile structures can simultaneously exist in the cell; the Gr layer plays an important role in preventing the diffusion of volatile silver filaments (Figure 24c), and the cell possesses many superior properties such as a superior on/off resistance ratio and a highly nonlinear resistive switch with a self‐selectivity of 10^10^ (Figure 24b). Based on these cells of memory, a 12 × 12 horizontal bar array was shown, and because of the high self‐selectivity of 10^10^, the “SKKU” code was successfully written with the use of 144 binary four letters (SKKU). This sets the stage for the development of energy‐efficient and large‐scale memory integration.^[^ ^192^ ^]^

The advantages of memristors based on 2DMs accomplished by vdW junctions hold great promise for the future devices.^[^ ^149^ ^]^ VdW heterostructure memories are a great solution to realize the high‐energy efficiency needed to develop high‐performance on‐chip memories for next‐generation computing to meet the data access needs of computing cells.^[^ ^152^ ^]^ VdW heterostructures’ memristors offer several advantages over single‐material system memristors such as tunability and flexibility; enhanced performance; tailored properties; multifunctionality; and reduced degradation.

VdW heterostructures allow researchers to combine different 2DMs with distinct properties. This tunability enables the optimization of memristor characteristics for specific applications. In contrast, single‐material‐system memristors are limited to the properties inherent in the chosen material. The use of diverse materials in vdW heterostructures allows for the exploitation of unique electronic properties from each layer. This can result in enhanced memristor performance, such as improved switching speed, lower energy consumption, and better retention characteristics compared to single‐material systems. With vdW heterostructures, it is possible to engineer the properties of the memristor by selecting and combining materials with specific electronic and optical characteristics. This tailoring of properties is challenging in single‐material systems where you are constrained by the intrinsic properties of the chosen material. VdW heterostructures enable the creation of multifunctional devices. By integrating different materials, researchers can design memristors that not only exhibit memristive behavior but also possess other functionalities, leading to more versatile and adaptable devices. The use of multiple materials in vdW heterostructures can mitigate some of the degradation issues observed in single‐material systems. For example, by carefully selecting materials with improved stability and endurance, researchers can enhance the overall durability and reliability of memristors.

In summary, the advantages of vdW heterostructures in memristors lie in their tunability, enhanced performance, tailored properties, multifunctionality, reduced degradation, and compatibility with 2DMs. These features make them promising candidates for a wide range of applications in emerging technologies.

## 5 Applications in Neural Networks and Artificial Intelligence

Neural network is a model used for computing that is made up of abundant connected nodes.^[^ ^193^ ^]^ From the point of information processing, it simulates how the neuronal network of the brain processes and memorizes information, builds an artificial neural network (ANN) model, and forms different networks on the basis of different connections.^[^ ^194^ ^]^ The system consists of three types of processing nodes: the input node, the output node, and the hidden node.^[^ ^195^ ^]^ The input node contacts the outside world and receives the stimulus from the outside world. The output node outputs the computing results of the system. The hidden node connects the input node and output node and is not visible outside the system.^[^ ^196^, ^197^ ^]^ The weight of connectional neurons reflects the strength of connections between neurons.^[^ ^194^ ^]^ The processing and representation of information are reflected into the connection relation of the network processing unit. The neural network does not have the problem caused by the division of memory and processor in the traditional von Neumann architecture and has great potential in complex problem processing, abundant parallel computing, and event‐driven computing.^[^ ^198^, ^199^ ^]^

2DMs and vdW heterostructures based on them attracted much attention for attractive electronic, mechanical, and photoelectric properties.^[^ ^200^, ^201^ ^]^ The vdW heterostructure offers unprecedented opportunities for the development of new electronic, optoelectronic, and spintronic devices with desired functionality and performance without worry about lattice mismatch. The defect‐free interface floating gate structure can be formed, and the number of interface traps or dipoles can be modulated.^[^ ^202^, ^203^, ^204^ ^]^ The vdW heterostructures with peculiar photoelectric and mechanical properties have great employment potential in the territories of spintronics, logic, memory, neuromorphic computing, and optoelectronics.^[^ ^204^, ^205^ ^]^

### 5.1 Principle

Between the end of the axon and the dendrite are gaps 20–40 nm wide called synapses, which can chemically or electrically transmit signals to make the membrane potential of the postsynaptic neuron more positive, reaching a threshold that triggers an action potential.^[^ ^206^ ^]^ As a result of neuron activity, synapses facilitate computation by changing the strength of their connections, called synaptic plasticity. Synaptic plasticity is considered the fundamental mechanism of biological brain learning and memory.^[^ ^206^, ^207^ ^]^

**Figure 25:**
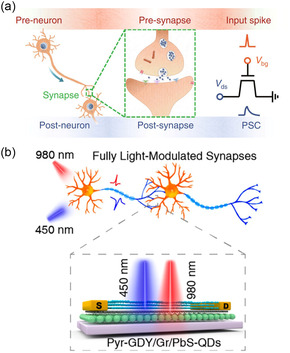
a) Diagram illustrating a biological synapse alongside the InSe artificial synaptic device. b) Schematic representation of the optical synapse and a diagram depicting a biological neural network and synapse. (Panel a) Reproduced under terms of the CC‐BY 4.0 license.^[^ ^213^ ^]^ Copyright 2020, The Authors. Published by Springer Nature Limited. (Panel b) is Reproduced with permission.^[^ ^229^ ^]^ Copyright 2021, American Chemical Society.

Currently, memristors and field‐effect transistors are often used for simulating synaptic function. Biological synaptic weights can be precisely adjusted by ion type (such as Ca^2+^, Na^+^, K^+^) and concentration.^[^ ^208^, ^209^ ^]^ Changes in electrical resistance of these devices are very similar to changes in biological synaptic weights (synaptic connection strength) and have been successfully used to simulate synaptic function.^[^ ^210^ ^]^ For hardware neuromorphic computer systems, it is extremely important to implement synaptic devices.^[^ ^211^ ^]^ The memristor is a kind of two‐terminal resistance device with a MIM structure. The formation and destruction of metallic filament caused by cation (such as Ag^+^) or hole movement in the functional layer by an electric field change the resistance of the memristor, which better simulate the gradual strengthening or weakening of biological synaptic weights under external stimuli.^[^ ^212^ ^]^ FETs are a kind of three‐terminal resistance device, the conductance of which is controlled by the gate voltage, as illustrated in **Figure** **25**. In FET, gate voltage input can be deemed as postsynaptic current triggered by the previous synapse, and its channel conductance can be regarded as synaptic weight.^[^ ^213^ ^]^ The synaptic properties of the device can be optimized by adjusting the material and structure of the device. 2DMs have an atomic‐level thickness that can observably decrease energy consumption, resulting from the SCE. Moreover, bandgap engineering can be realized by changing the layer number of 2DMs, so that different resistance or conductance states can be realized in the case of low power consumption, to reduce the device size and further increase the integration.^[^ ^207^, ^214^, ^215^ ^]^

### 5.2 Vision Applications

Despite the great advances in processing optical and electrical signals based on neural networks, transforming optical images into electrical signals remains an obstacle, especially in time‐constrained tasks.^[^ ^216^, ^217^, ^218^, ^219^ ^]^ For vision applications, the essential features of vdW heterostructures contribute to enhanced image processing and recognition. This will include considerations such as efficient charge transport, tunable bandgaps, and integration capabilities to optimize neural network architectures for visual tasks.^[^ ^220^ ^]^ Traditional vision chips cannot preprocess visual data, which limits temporal resolution and leads to redundancy of recorded image data.^[^ ^221^ ^]^ The use of new physics phenomena of emerging materials on an atomic scale and the structures can provide opportunities to achieve neuromorphic vision sensors.^[^ ^222^ ^]^ It has been shown that the image sensors themselves can form a neural network based on a photodiode array of 2DMs, simultaneously detecting and processing optical images without delay. The synaptic weights of the network can be consecutively tuned by a photoresponsivity matrix.^[^ ^218^ ^]^

**Figure 26:**
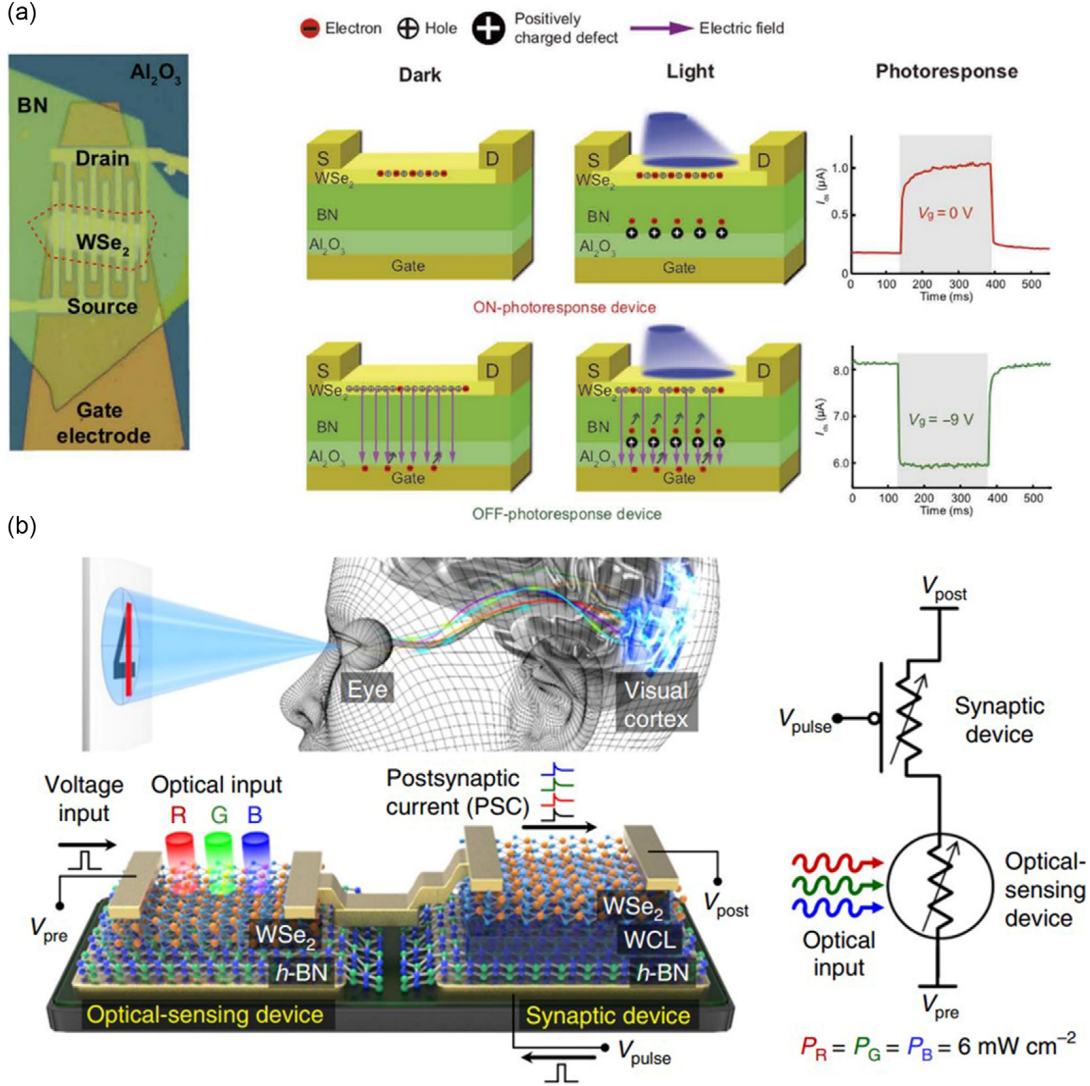
a) Optical image displaying a retinomorphic device fabricated on a vdW vertical heterostructure. The operational mechanism and photoresponse of the ON‐ and OFF‐photoresponse devices under zero‐and negative‐gate voltages, respectively. Positive (negative) Δ*I*_ds_ corresponds to ON‐photoresponse (OFF‐photoresponse). The shaded areas indicate the duration of light exposure. Reproduced under terms of the CC‐BY 4.0 license.^[^ ^222^ ^]^ Copyright 2020, The Authors. Published by American Association for the Advancement of Science. b) Schematic representation of the human optic nerve system, the h‐BN/WSe_2_ synaptic device integrated with the h‐BN/WSe_2_ photodetector, and the simplified electrical circuit for the ONS device. In this instance, dot lasers with wavelengths of 655, 532, and 405 nm were employed, each with a fixed power density (P) of 6 mW cm^−2^. Reproduced under terms of the CC‐BY 4.0 license.^[^ ^225^ ^]^ Copyright 2018, The Authors. Published by Springer Nature Limited.

VdW vertical heterostructures with abundant electronic and photoelectronic properties can be used to simulate the hierarchical structure and function of retinal neurons and then realize neural network vision sensors in a natural way.^[^ ^222^ ^]^ Wang et al. produced the WSe_2_/h‐BN/Al_2_O_3_ vdW vertical heterostructure device (**Figure** **26**), which can realize the transformation of light and electrical signals and show negative photoresponse and positive photoresponse according to the gate voltage, similar to the biological characteristics of the bipolar cells (ON cells and OFF cells).^[^ ^222^, ^223^, ^224^ ^]^ Former studies showed that photoinduced reduction of carrier mobility in low‐dimensional materials can suppress device currents, but the response time cannot compare with the on‐photoresponse device.^[^ ^200^ ^]^ But using the sharp atomic interface of vdW heterostructure and nanometer thickness, Al_2_O_3_ layer can achieve fast photoresponse speed (the off‐photoresponse time is less than 8 ms) and the devices have low energy consumption and fast operating speed.^[^ ^222^ ^]^ Further improvements are expected by decreasing the contact resistance of WSe_2_ to metal electrodes, creating high‐quality interfaces between h‐BN and WSe_2_ and distributing or concentrating defects in h‐BN.^[^ ^222^ ^]^ Seo et al. combined an optical sensor device with a synaptic device on the h‐BN/WSe_2_ vdW heterostructure to construct an optical synaptic device, realizing the functions of both the optical sensor and synapse.^[^ ^225^ ^]^ The vdW heterostructure of this device has no interfacial defects and thus allows the modulation of interface traps. The optic–neural network (ONN) based on this device can be used for emulating the human visual nervous system to recognize colored and color‐mixed patterns. The devices modulate the conductivity of the WSe_2_ channel (weight of synapse) by controlling the number of electrons from the weight control layer located in h‐BN.^[^ ^225^ ^]^

Zhang et al. provided a simple and reliable method to produce 6 cm × 6 cm‐size graphdiyne (GDY)/Gr vertical heterostructures and constructed photoelectron synapse device arrays based on this structure.^[^ ^226^ ^]^ It can realize some typical synaptic behaviors such as associative learning, inhibitory postsynaptic current and excitatory postsynaptic current, and so on.

A convolutional neural network (CNN) was constructed by extracting parameters from this photoelectric synaptic device to recognize handwritten numbers of 28 × 28 pixels in the Modified National Institute of Standards and Technology (MNIST) dataset. After 40 epochs of training, the recognition accuracy (the ratio of predicted classes to correct classes) of the noise‐free test data set reached 94.3%, slightly lower than the maximum recognition accuracy (97.5%) of the same network with ideal floating‐point numerical accuracy.^[^ ^226^ ^]^ Jeon utilized a self‐assembled monolayer material to passivate the interface traps on FeFETs, which improves the performance of a FeFET, successfully achieving excellent optic‐neural synapse features, including symmetric and linear weight updates, and so on. They measured the value of subthreshold swing, which reflected a decrease in the density of interface traps.^[^ ^227^ ^]^

Now MVDWHs also demonstrate great potential in the construction of visual neural networks. Goossens et al. successfully produced a 388 × 288 pixel‐scale image sensor array based on monolithic integration of a CMOS integrated circuit with Gr, in which the Gr/PbS QD MVDWHs exhibit wideband light response in ultraviolet to infrared (300–2000 nm) light. The spectral range is controlled by the size and material of the QD, and this method can be extended to other types of sensitized materials that can adjust the spectral range of the sensor element.^[^ ^228^ ^]^ Hou et al. also produced a synapse device based on graphdiyne/Gr/PbS QD MVDWHs, which can exhibit excitatory and inhibitory synaptic behavior through controlled positive/negative photoresponse to different wavelengths of light stimulation in the optical pathway, which is critical for complex neuromorphic computation.^[^ ^229^ ^]^

### 5.3 Logic Applications

**Figure 27:**
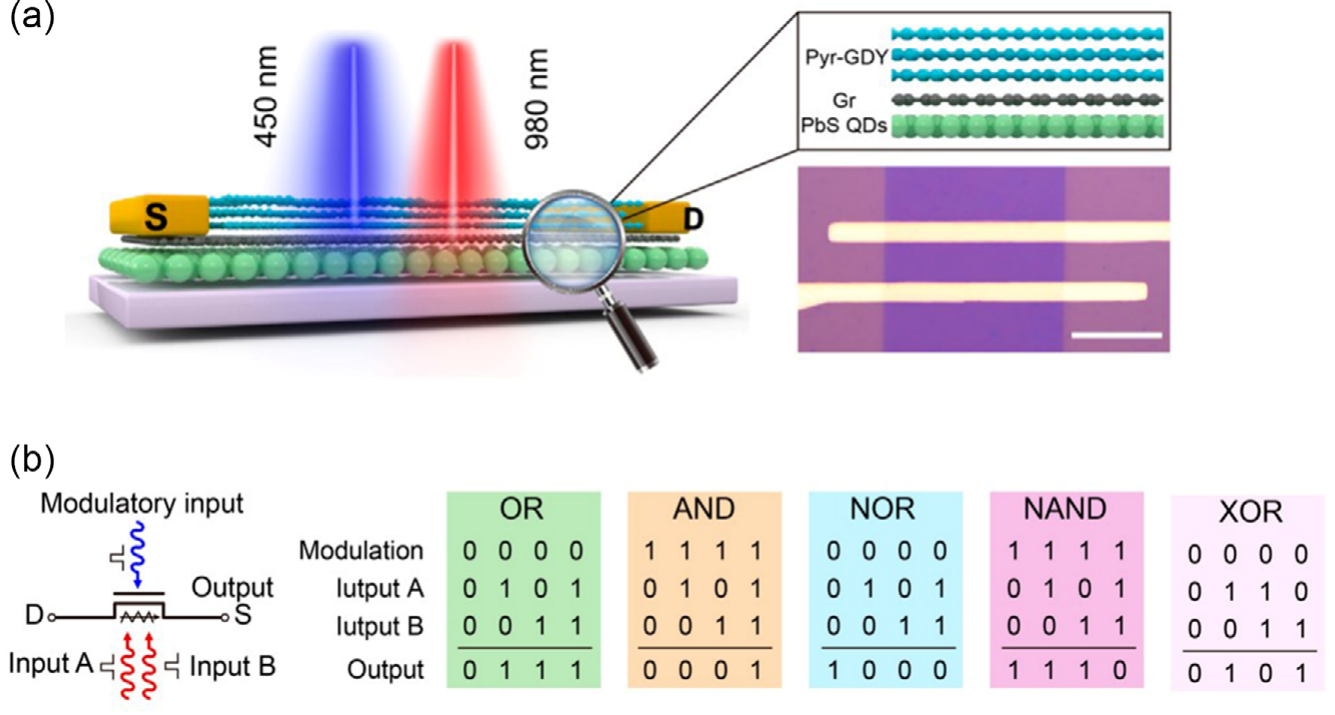
a) Assessments and optoelectronic characteristics of the optical synapse built on the Pyr‐GDY/Gr/PbS QD heterostructure. Depiction of the optical synapse and its optical microscope image. The scale bar denotes 25 μm. b) Schematic representation of the operational logic functions within the optical synapse. The truth tables showcasing the optical functions of “OR,” “AND,” “NOR,” “NAND,” and “XOR” are presented on the right panel. Reproduced with permission.^[^ ^229^ ^]^ Copyright 2021, American Chemical Society.

Artificial devices that can realize both synaptic and logical functions are very important for neuromorphic computing, which can observably improve the information processing capability of neuromorphic computing.^[^ ^230^ ^]^ In the context of logic applications, the specific characteristics of vdW heterostructures are paramount for logic operations. Emphasis will be placed on factors such as low power consumption, high carrier mobility, and the potential for achieving multiple logic states, illustrating their relevance in advancing computational logic frameworks. Recently, many researchers have realized representative logic functions like “OR”, “NOR”, “AND”, and “NAND” in artificial synaptic devices.^[^ ^230^ ^]^ The optical synapse device made by Hou et al. mentioned above also integrates logic functions.^[^ ^229^ ^]^ Two light inputs A and B produce optical signals of 980 nm or 450 nm wavelength respectively and changing the modulatory input (no signal, 980 nm optical signal, or 450 nm optical signal) can change the signal at the output, allowing it to perform these typical logic functions respectively. The different value between the output value and the threshold value determines the performance of logic function devices. So, these logic function devices need to have a characteristic response to multiple inputs and modulators (**Figure** **27**).^[^ ^229^ ^]^

**Figure 28:**
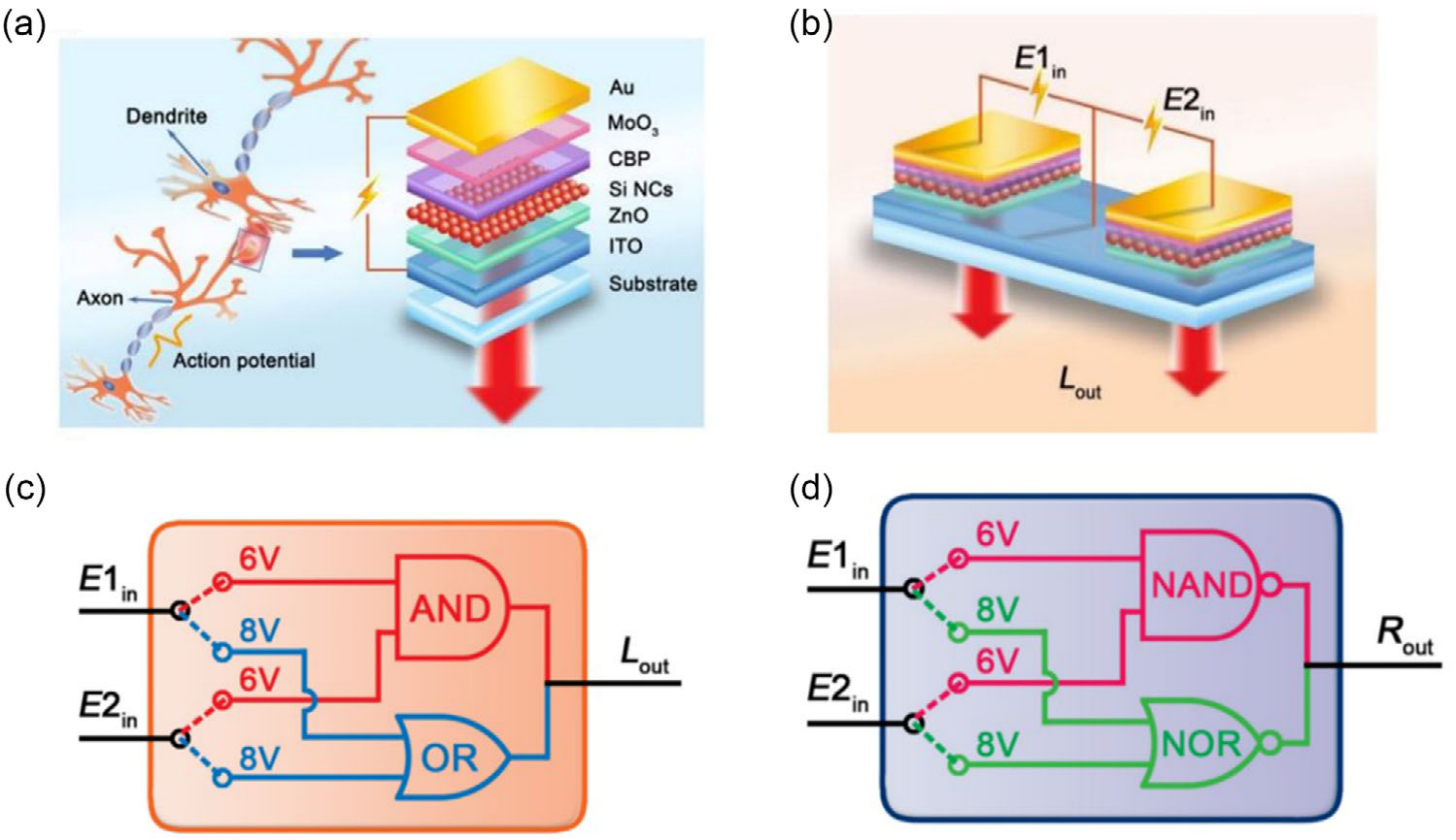
a) Left: An illustration of biological neurons linked through a synapse. Right: A schematic of an EL synaptic device featuring a multilayer structure composed of ITO/ZnO/Si NCs/CBP/MoO_3_/Au. b) Illustration of a pair of interconnected synaptic devices configured symmetrically, demonstrating optical power activation in response to 8 V electrical spikes. c) Operational schematic for the “AND” and “OR” gates. d) Operational schematic for the “NAND” and “NOR” gates. Reproduced with permission.^[^ ^231^ ^]^ Copyright 2020, Elsevier Ltd.

Pi et al. explored the application of optoelectronic integration in neuromorphic logic computing using optoelectronic synaptic devices made of silicon nanocrystals.^[^ ^231^ ^]^ **Figure** **28** shows two connected light outputs electrically stimulated synaptic devices based on silicon nanocrystal. Electrical spikes act on two synaptic fronts (E1_in_ and E2_in_) and induce nonvolatile light output (*L*_out_), which is defined as “1” when the value is greater than the threshold power of 1 μW, otherwise as “0”.^[^ ^231^ ^]^

**Figure 29:**
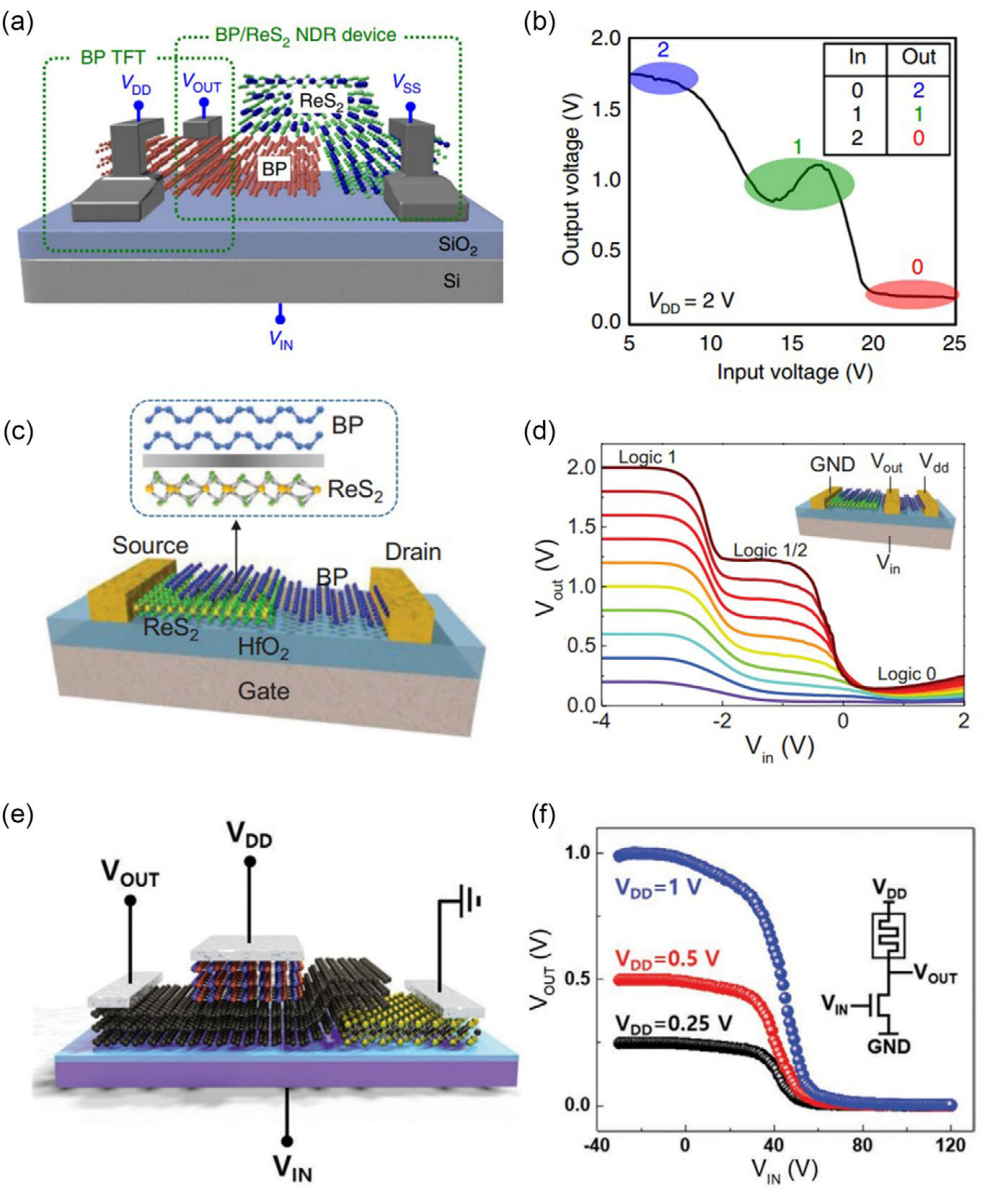
a) Illustration of the ternary inverter. b) Characteristic plot of *V*_IN_ versus *V*_OUT_ for the ternary inverter, accompanied by an inset featuring the input–output table of the ternary inverter. Reproduced under terms of the CC‐BY 4.0 license.^[^ ^232^ ^]^ Copyright 2016, The Authors. Published by Springer Nature Limited. c) Diagram illustrating BP/ReS_2_ heterostructure devices and a schematic cross‐sectional view of the junction interface. The drain electrode is located on the BP layer, and the source is on the ReS_2_ layer. d) Plot of *V*_OUT_ versus *V*_IN_ for ternary inverters utilizing the BP/ReS_2_ heterostructure with in‐series BP FETs and *V*_DD_ ranging from 0.1 to 2 V. Inset: Circuit diagram of the ternary inverter. Reproduced with permission.^[^ ^233^ ^]^ Copyright 2020 WILEY‐VCH Verlag GmbH & Co. e) Diagram illustrating the memtransistor's operation as a logic inverter. The h‐BN memristor serves as the resistor load, and the MoS_2_ transistor operates as the switching transistor. f) Output characteristics of the device at *V*_DD_ = +0.25, +0.5, and +1 V, respectively. The inset shows the circuit diagram of the logic inverter. Reprinted with permission.^[^ ^234^ ^]^ Copyright 2020, WILEY‐VCH Verlag GmbH & Co. KGaA.

Shim et al. showed a ternary inverter for multivalued logic applications by making a negative differential resistor based on BP/ReS_2_ heterostructure.^[^ ^232^ ^]^ The power voltage (*V*_DD_), the input voltage (*V*_in_), and the output voltage (*V*_out_) are respectively applied to the source electrode, the back gate, and the shared electrode. The relationship between the *V*_in_ and *V*_out_ is shown in **Figure** **29**, proving that the device can be used as a ternary logic inverter.^[^ ^232^ ^]^ Xiong et al. also used a 2D BP/ReS_2_ vdW heterostructure to demonstrate that the structure can be used for nonvolatile ternary logic operations.^[^ ^233^ ^]^ The ultrathin oxide layer located at the BP can be used for trapping charges and tunneling between the bands. Meanwhile, they used the structure to make an artificial synaptic device, which could simulate the trilingual synaptic response by modulating the input signals. The three‐level input voltage pulse producing the output characteristics is shown in Figure 29, and the intermediate logic is also clearly visible. Moreover, the artificial neural network (ANN) made by the array of electronic synapses shows that the recognition accuracy of handwritten digital data sets is up to 91.3%.^[^ ^233^ ^]^ Park et al. produced a programmable memtransistor device with high computational capability by integrating a memristor with a transistor.^[^ ^234^ ^]^ The memristor part of the device is made of h‐BN stacked with Gr, while the other transistor part is made of Gr stacked with MoS_2_, with the Gr connecting the two parts. This device can also be used for logical applications, as shown in Figure 29; the back gate acts as the input signal (*V*_in_), the shared part made by Gr acts as the output signal (*V*_out_), and changing the top electrode voltage of the memristor (*V*_DD_) can change the output signal *V*_out_. The figures show the characteristic curve of this memtransistor device at *V*_DD _= +0.25, +0.5, and +1 V, showing the high‐output and low‐output states.^[^ ^234^ ^]^

Cheng et al. produced a vertical CsPbBr_3_ QDs/MoS_2_ vdW heterostructure optoelectronic device.^[^ ^198^ ^]^ They assembled the CsPbBr_3_ QDs on the 2D MoS_2_ layer to develop a heterostructure interface that is used for transporting photocarriers with high efficiency. Experiments showed that applying spikes on both drain and gate terminals can modulate drain–source current and applying external light stimulus can also tune conductance. Optical and electrical synergy can successfully simulate the electro‐optical modulation logic function by inputting a light stimulus on the heterostructure channel and two electrical stimuli on the drain electrode and the gate electrode.^[^ ^198^ ^]^

### 5.4 Computing Applications

Neuromorphic computing architecture has been developed using mature analog, digital technologies and CMOS devices.^[^ ^235^, ^236^, ^237^ ^]^ Concerning computing applications, the distinctive attributes of vdW heterostructures are pivotal for accelerating computational processes. This will encompass characteristics such as high carrier mobility, reduced heat dissipation, and scalability, outlining their potential to significantly impact the field of artificial intelligence computing. For example, IBM produced a chip called TrueNorth which contains 2.56 million synapses made up of 5 billion transistors. It can input 30 frames of 400 × 240 pixels of video per second and consume 63 mW of power.^[^ ^238^ ^]^ But these architectures are difficult to reduce energy consumption and do not mimic biological synapses, which cannot compare with the biological systems.^[^ ^239^ ^]^

**Figure 30:**
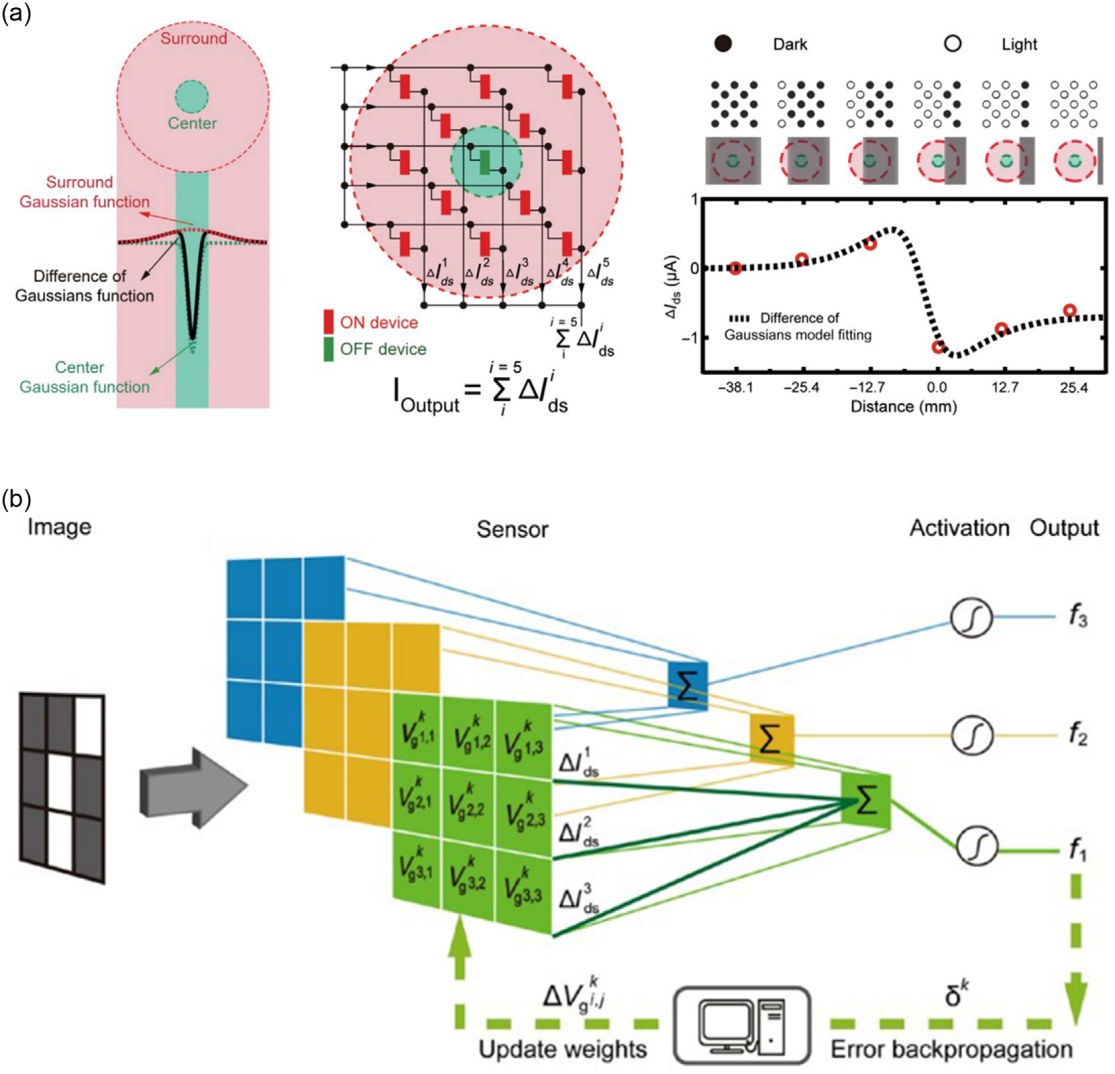
a) RF with a green center and pink surrounding regions. In the left panel, the DGM of the RF depicts the distribution of responsivity. The central panel showcases the vision sensor and its corresponding output, where an OFF‐photoresponse device is centrally positioned and surrounded by ON‐photoresponse devices. The vision sensor's output represents the cumulative current across all devices. The right panel displays the outputs of the artificial RF with a contrast‐reversing edge transitioning from the left to the right side. The upper circle array symbolizes light sources, with solid circles indicating light‐on and empty circles representing light‐off. b) Training process of the vision sensor at each epoch, with varying color maps representing different convolution kernels. ‘k’ denotes the count of training, while ‘i’ and ‘j’ indicate the position of each pixel in the sensor. Reproduced under terms of the CC‐BY 4.0 license.^[^ ^222^ ^]^ Copyright 2020, The Authors. Published by American Association for the Advancement of Science.

As mentioned above, Wang et al. produced WSe_2_/h‐BN/Al_2_O_3_ heterostructure devices to mimic some characteristics of bipolar cells.^[^ ^222^ ^]^ They produced an array (the 12 on‐photoresponse devices encompass the central off‐photoresponse device) that can simulate a biological receptive field. The change in current is most pronounced when light is swept across a central device. The real‐time change of the output current from the array represents the dynamic response to the change of light patterns, which can be used for edge detection of objects. The CNN can also be formed by the retinal morphologic vision sensor to classify the target image, in which the gate voltage of each pixel can be adjusted to update the weights. By taking the dot product of synaptic weights and the perceived information they calculate the output current. Using backpropagation, the gate voltage of each pixel can be adjusted and the weight updated after each epoch (**Figure** **30**).^[^ ^222^ ^]^

Seo et al. fabricated two devices based on WSe_2_/h‐BN and MoS_2_/h‐BN vdW heterostructures and connected them together by two shared electrodes to form a synaptic device.^[^ ^210^ ^]^ WSe_2_ and MoS_2_ on the two devices were for electron and hole transport, respectively. The signaling pathways formed by the two heterostructures showed potentiation and depression operations, respectively. The two shared electrodes of the two channels are regarded as the presynaptic and postsynaptic terminals, and each channel has a gate electrode to control the weight of the synaptic device. They formed an ANN made up of 400 input nodes, 5 output nodes, and 400 × 5 nodes in the hidden layer that connects these two types of nodes and the ANN can be used for acoustic pattern recognition. They converted the speech signal into an acoustic image. Each pixel of the acoustic image corresponds to a voltage that acted as the input signal of the ANN. The ANN multiplied the synaptic weight with the input voltage and then summed it up to get the output current. Finally, they used the backpropagation algorithm to update the synapse weights. As a result of training and inference tasks, hybrid synaptic device array recognition rate reached 93.8% in acoustic pattern recognition tasks (close to 95.3% for software‐based neural networks).^[^ ^210^ ^]^

**Figure 31:**
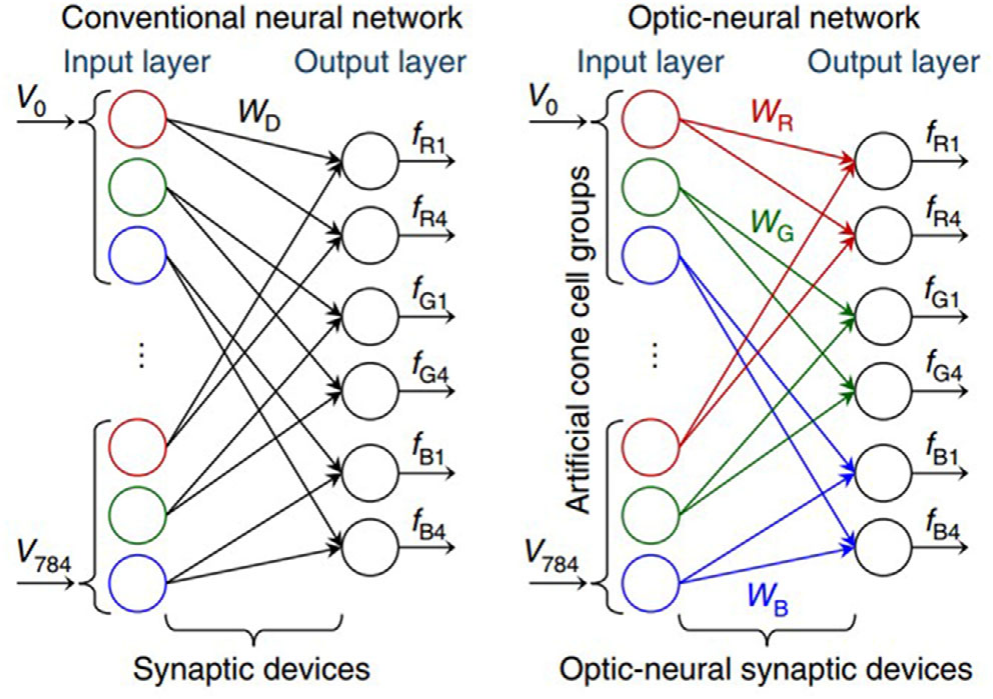
The left panel shows a conventional neural network in which the input layer consists of arrays of neurons with colour‐filtering capabilities to mimic the biological cone cells of the human visual system, but the synaptic connections do not have a light‐sensing function, and the right panel shows an optical neural network in which each group of artificial cone cells consists of three cells sensing red, green and blue light, respectively. Reproduced under terms of the CC‐BY 4.0 license.^[^ ^225^ ^]^ Copyright 2018, The Authors. Published by Springer Nature Limited.

Seo et al. also used the extracted device parameters to design an ANN and applied the ANN to mixed color pattern recognition tasks.^[^ ^225^ ^]^ The number of target colors are identified from a complex mix of numbers. In visual neural networks, optical sensing is added to synaptic connections. The artificial pyramidal cell group consisted of three neurons, each comprising a 28 × 28 array, in which each cell responded to different color light. They used MNIST datasets for pattern recognition tasks (identifying objective patterns from complex images). The ONN recognition rate exceeded 90% after the 50th generation and then stabilized. In contrast, neural networks composed of synaptic elements with no optical detection had a recognition rate of less than 40% (**Figure** **31**).^[^ ^225^ ^]^

When designing vdW heterostructures for neural networks, a holistic consideration of key factors is imperative, encompassing material selection, layer thickness, and interlayer coupling strength. This comprehensive guide will delve into these aspects to assist researchers in optimizing heterostructures for multifunctional neural networks.

The choice of materials is pivotal for the performance of heterostructures. For electron transport functionality, opt for materials with high conductivity, such as metals or highly conductive semiconductors like Gr. Simultaneously, for visual applications, semiconductor materials with excellent photoelectric response, including MoS_2_ and MoSe_2_, are outstanding choices to achieve photoelectric conversion and light control functions. For logic and computing applications, materials with RS characteristics, such as ferroelectric materials, are beneficial. Recent studies have demonstrated signal input, processing, and output within the same device, necessitating materials with both photoresponsive and resistive characteristics, achievable through the selection of materials like In_2_Se_3_. Combining materials with diverse characteristics proves advantageous for simulating synaptic functions. Given the multifunctionality of heterostructures, a profound understanding of each material's characteristics is crucial.

Layer thickness directly influences electron transport and optical performance. In terms of electron transport, optimizing layer thickness ensures appropriate electron transport paths and interlayer isolation. In natural neural networks, short‐term and long‐term effects exist. Adjusting layer thickness enables the simulation of short‐term and long‐term memory, with layer thickness strongly affecting the ferroelectric properties of some ferroelectric materials. For optical applications, adjusting layer thickness maximizes photon absorption while maintaining transparency when necessary. This involves a deep understanding of the balance between optical and electrical properties to meet specific application requirements.

Interlayer coupling strength is crucial for electron transfer rates and information transfer efficiency. By adjusting interlayer coupling strength, appropriate charge transfer rates can be achieved, ensuring effective information transmission and processing in neural networks. Moreover, the modulation of coupling strength can be employed to optimize connections between neurons in the neural network, enhancing network flexibility and adaptability.

By carefully considering material properties, layer thickness, and interlayer coupling strength, the optimal design of vdW heterostructures for neural networks can be achieved. This not only provides innovative solutions for optoelectronics and artificial intelligence but also establishes a solid foundation for efficient and flexible neural networks. This comprehensive guide aims to steer future research and design efforts, promoting the widespread application of vdW heterostructures in neural networks.

## 6 Conclusion and Outlook

The excellent optical, electronic, thermal, and magnetic properties of vdW heterostructures have a great impact on many research fields, including quantum theory, materials design, nanotechnology, and electronic engineering. In this article, we summarize the latest technological breakthroughs and advanced applications in vdW heterostructures and review the advantages and prospects of vdW heterostructures from the aspects of fabrication methods, device applications, and theoretical predictions. A large number of new physical effects are found, and many ingenious designs will be used for the next generation of high‐performance devices. First, vdW heterostructures provide an idea for the fabrication of high‐efficiency functional devices based on their excellent optoelectronic response. For example, infrared detectors of vdW heterostructure possess fast response speed and high sensitivity. Solar cells of vdW heterostructures also show high energy conversion efficiency. Second, the ferroelectrics, ferromagnets, and memristors of vdW heterostructures provide a potential application for the high‐integrity information storage. The as‐formed memory devices are expected to be used in fast writing/reading and long‐term storage. Third, we review the latest development of vdW heterostructures in neural networks. Some vdW heterostructures exhibit similar electrical behaviors to neural synapses. They show the potential applications for machine learning by simulating the human brain.

In addition, we review our recent works on the 2DEG‐based vdW heterostructures. They were prepared on KTO and STO surfaces using the AIBA method. Compared with other vdW heterostructures, the 2DEG‐based vdW heterostructures prepared by this method had a nanoscale junction contract at the interface. In WSe_2_/2DEG heterostructures, we discovered the unusual physical phenomena: chargeable photoconductivity and GBU‐PhMR. Our works open a new possibility for self‐driven energy harvest and light‐tunable spintronic devices.

In summary, the emergence of vdW heterostructures has presented significant opportunities and challenges for researchers. This innovative paradigm offers a promising avenue for exploring novel electronic devices and functionalities. The ability to combine diverse 2DMs with tailored properties opens up new possibilities for designing devices with enhanced performance and multifunctionality. VdW heterostructures offer the ability to combine different 2DMs, allowing for the tailoring of electronic, optical, and thermal properties for specific applications. The versatility of vdW heterostructures enables the creation of multifunctional devices by integrating diverse materials with complementary functionalities. VdW heterostructures can be integrated into existing semiconductor technologies, enhancing compatibility with established processing methods and device architectures. Optimizing material combinations in vdW heterostructures may lead to improved energy efficiency in electronic and optoelectronic devices.

Despite the rapid development of vdW heterostructures in the past decade, there still remain some issues that need to be solved for future applications. Achieving precise control and scalability in the fabrication of vdW heterostructures remains a challenge, impacting reproducibility and large‐scale applications. Controlling the interface between different layers to avoid defects and manage interlayer interactions is critical for optimizing electronic properties. Ensuring uniform thickness and minimizing defects across large‐area vdW heterostructures is challenging, affecting electronic and optical performance. VdW heterostructures can be sensitive to environmental conditions, requiring strategies to enhance stability and performance under varying circumstances. We raise the following future perspectives for the research on vdW heterostructures. The first one is to develop more reliable preparation methods. The method of mechanical transfer is the most widely used in the vdW heterostructure fabrication at present, while unavoidable interface distortion and material damage will greatly affect the device performance. Although high‐quality 2D heterostructures can be produced by the CVD method, the high cost of CVD prevents it from being a widespread method. Additionally, a practical 2DM physical model needs to be established to explain the carrier behavior in the vdW heterostructure. Single‐layer 2DMs are usually ultrathin and down to an atomic scale. Thus, both Coulomb interactions and quantum mechanical effects need to be considered. Although some studies attempt to explain the physical theoretical basis of 2DMs,^[^ ^240^, ^241^, ^242^, ^243^ ^]^ these theories still need to be proved by the fitting of experimental data and theoretical models. Appropriate semiconductor physical models are of great significance for understanding heterostructure behavior and optimizing device parameters. Finally, more interesting, complex structures need to be proposed, not just 2D, *n*D/*n*D(*n* = 0, 1, 2, 3) should be considered. Not just two materials, more stacks would have more interesting couplings. Not only single heterostructures but also the union between multiple heterostructures is a problem worth thinking about, but these complex systems are undoubtedly a great challenge. Currently, the vdW heterostructure is still limited to the experimental stage. In the future, vdW heterostructures may have different directions of development, one is to explore integration for practical applications. Although the integration of Gr and MoS_2_ has shown promising results, there is still a need to explore the integration of a wider variety of 2DMs, as well as one‐step direct growth and multistep growth processes for vdW heterostructures on this basis. This is critical to the process of getting vdW heterostructures from the lab to the factory. Second, there is a need for a more in‐depth study of heterostructures in 2DMs, including the principles and physical mechanisms. The discovery of Gr, a monolayer material once thought to be unstable and the discovery of Gr, a monolayer material once thought to be unstable and the resulting vdW heterostructure based on it has brought great benefits to the study of the physical properties of low‐dimensional and quantum systems, where a considerable number of undiscovered exotic phenomena have been found, and in‐depth studies of vdW heterostructures will contribute to the understanding of the underlying laws of physics.

## Conflict of Interest

The authors declare no conflict of interest.
